# Patient Acceptance and Barriers to IoT Usage in Health Care: Systematic Literature Review

**DOI:** 10.2196/81260

**Published:** 2026-07-31

**Authors:** Zaenal Arifin, Putu Wuri Handayani

**Affiliations:** 1 Doktor Ilmu Komputer, Faculty of Computer Science, University of Indonesia, Fakultas Ilmu Komputer, Kampus UI Depok, Depok, Jawa Barat, 16424, Indonesia, 62 21 786 3419, 62 21 786 3415

**Keywords:** internet of things, healthcare IoT, technology acceptance, patient acceptance, patient-centric, systematic review, telemedicine, eHealth, mHealth, smart hospital, mobile health, mobile phone

## Abstract

**Background:**

The Internet of Things (IoT) represents a transformative paradigm in health care service delivery, offering unprecedented potential to enhance quality of care, operational efficiency, and patient outcomes through interconnected devices and real-time data analytics. Despite rapid adoption, implementation depends on patient acceptance as end users. While literature extensively documents technical and institutional perspectives, a comprehensive understanding of patient acceptance factors remains fragmented, with high technology abandonment rates. Systematic synthesis of patient perspectives is critically needed to inform user-centered design, effective implementation strategies, and supportive policy frameworks.

**Objective:**

This systematic literature review aims to identify and synthesize factors influencing patient acceptance of IoT technology in health care services, barriers hindering adoption, and effective strategies for enhancing acceptance.

**Methods:**

Following PRISMA (Preferred Reporting Items for Systematic Reviews and Meta-Analyses) 2020 guidelines, we systematically searched eight electronic databases (PubMed/MEDLINE, Scopus, IEEE Xplore, Web of Science, ScienceDirect, ACM Digital Library, ProQuest, and Google Scholar) for empirical studies published between January 2016 and December 2024. Inclusion criteria encompassed peer-reviewed empirical research examining patient perspectives on IoT technology in health care services, published in English or Indonesian. From 2537 initially identified papers, 62 studies met inclusion criteria after systematic screening and full-text evaluation. Quality assessment was conducted using the Mixed Methods Appraisal Tool.

**Results:**

The 62 included studies represented diverse geographic contexts (Asia, Europe, North America, and the Middle East) and methodological approaches. Quality assessment revealed 45 (73%) studies of good-to-excellent quality. Perceived usefulness emerged as the strongest acceptance facilitator, identified in 55 of 62 (89%) studies, followed by perceived ease of use in 47 (76%) studies, and trust and security in 42 (68%) studies. Cost-effectiveness was identified in 32 (52%) studies as an important consideration. Primary barriers included data security concerns in 26 (42%) studies, privacy issues in 24 (39%) studies, lack of digital literacy in 22 (36%) studies, and resistance to change in 20 (32%) studies. Interoperability issues and high costs were identified in 19 (31%) studies and 18 (29%) studies, respectively. User-centered design was the most frequently recommended enhancement strategy in 20 (32%) studies, followed by user-friendly interface development in 19 (31%) studies, digital literacy programs in 18 (29%) studies, and health care professional involvement in 15 (24%) studies. Digital literacy functioned as a significant moderator, and trust served as both direct predictor and mediator.

**Conclusions:**

Patient acceptance of IoT in health care represents a complex, multidimensional phenomenon requiring holistic approaches that integrate user-centered technology design, comprehensive digital literacy programs, trust-building mechanisms, and supportive policy frameworks. Successful implementation necessitates multilevel strategies addressing individual, organizational, and system factors simultaneously. With evidence-based, patient-centered approaches, IoT technology holds substantial potential to transform health care delivery into more proactive, personalized, and accessible services.

## Introduction

### Background

The technological developments of the coming decade demonstrate fundamental transformations that will radically change how humans live, work, and interact. According to the McKinsey Global Institute (2024) report [1], waves of technological innovation represent not merely incremental changes, but a new paradigm that will integrate AI, quantum computing, and sustainable technologies into almost every aspect of human life. This condition is driven by multidisciplinary convergence between computer science, biotechnology, nanotechnology, and AI, opening new possibilities previously considered impossible. Sustainable technology has become a central paradigm in future innovation development, with renewable energy, green computing, and environmental solutions becoming the primary focus of global research. The Intergovernmental Panel on Climate Change (IPCC) report [2] confirms that technological innovation will be key in climate change mitigation, with projections of carbon emission reductions of up to 45% through green technology implementation by 2040. Based on the context of sustainable technology as the central paradigm of future innovation, the health care sector faces urgent real challenges that require resolution through modern technology implementation. One major problem currently faced by the health care sector is the increasing older adult population experiencing multiple chronic conditions (MCCs) such as hypertension, arthritis, heart disease, cancer, diabetes, and chronic kidney disease. This multimorbidity condition impacts increased mortality, disability, decreased physical function, and reduced patient quality of life [3]. Future technology trends addressing these issues include the internet of medical things (IoMT) for continuous monitoring [4] and expansion of telemedicine and telehealth, enabling remote consultations, reducing patient waiting times, and expanding access to specialists [5]. This digital transformation specifically targets reducing the older adult population health care costs while improving their outcomes and quality of life through personal and sustainable technology solutions.

The rapid advancement of digital technology in recent decades has transformed various aspects of human life, including the health care sector. One of the most promising innovations in health care service transformation is the Internet of Things (IoT). IoT presents significant paradigm changes in health care service delivery, driven by the convergence of interconnected devices, data analytics, and communication networks. This technological synergy enables real-time patient health monitoring, remote chronic condition management, and improved efficiency in health care service operations. IoT infrastructure facilitates communication between various entities, including individuals and medical devices such as wireless sensors, thereby promoting increased access to quality and affordable health care services [6]. IoT implementation spans various health care service domains, offering innovative solutions for remote patient monitoring, medication adherence, and chronic disease management [7].

Although AI offers tremendous potential in the health care sector, the focus on IoT stems from its direct and practical applications in addressing current challenges in health care systems [8]. IoT provides basic infrastructure for collecting, transmitting, and analyzing patient data, building foundations for more sophisticated AI applications [9]. Integration of IoT devices, such as wearable sensors and remote monitoring systems, facilitates continuous data collection about physiological parameters, activity levels, and environmental conditions [10]. This real-time data flow enables proactive monitoring and early detection of health anomalies, reducing the need for frequent hospital visits and improving patient outcomes [10].

**Figure 1.**
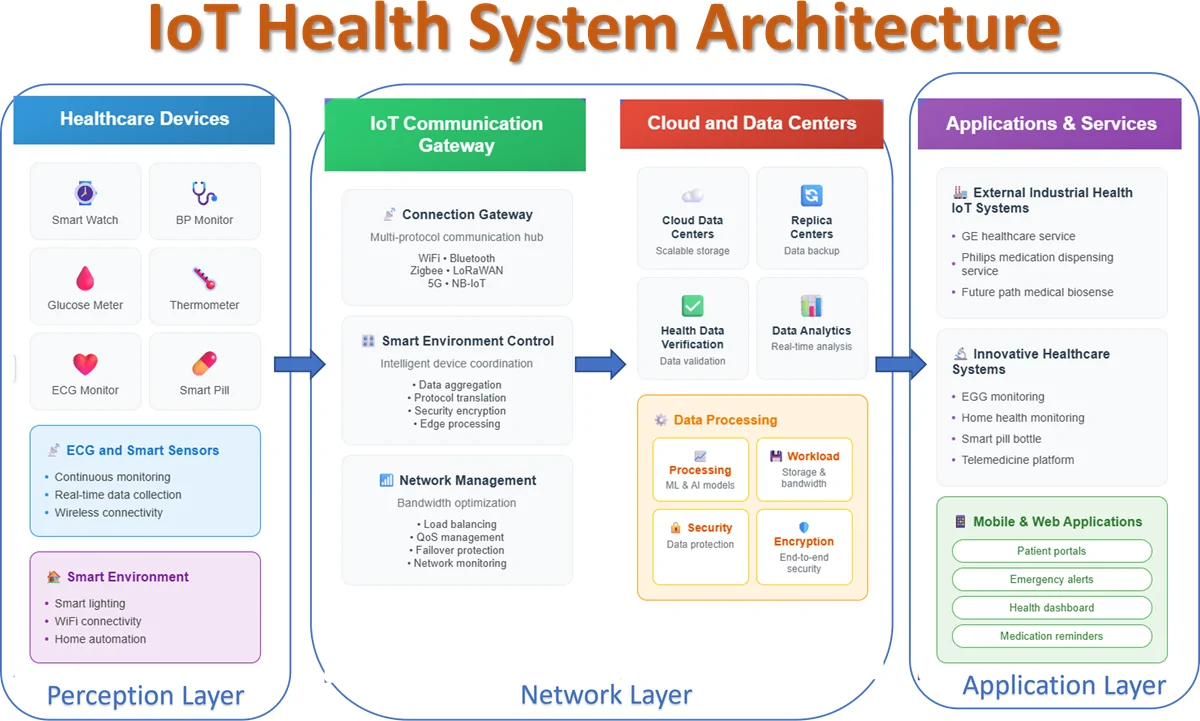
IoT health system architecture. BP: blood pressure; ECG: electrocardiogram; GE: General Electric; IoT: Internet of Things; QoS: quality of service.

IoT refers to networks of physical devices connected through the Internet, enabling real-time data collection and exchange [11]. In academic contexts [11], IoT is described as a computing paradigm encompassing three main dimensions: communication, semantics, and interconnectivity. The communication dimension refers to devices’ ability to exchange information through diverse network protocols. The semantic dimension focuses on interpretation and use of meaningful data, while interconnectivity refers to devices’ ability to connect and interact in complex and dynamic ecosystems. In health care service contexts, IoT implementation (Figure 1) offers great potential for improving care quality, operational efficiency, and overall patient experience [12].

The development of health care IoT market share shows very significant growth with latest data from Transforma Insights reporting that by the end of 2023 there were 16.1 billion active IoT devices worldwide, with growth projections reaching 399 billion devices in 2033 with a compound annual growth rate of 10%, while the total IoT market including connectivity modules and related core applications was valued at US $335 billion in 2023 and projected to increase to US $934 billion in 2033 [13]. Health care IoT adoption acceleration has been further accelerated by COVID-19 pandemic impacts, emphasizing urgent needs for remote health care solutions and digital technologies that can support health care service continuity in situations of physical and social restrictions [14].

Despite the transformative potential of IoT technology in health care services being extensively documented and discussed from technical, clinical, and institutional perspectives in various scientific literature, a comprehensive understanding of acceptance from patient perspectives as end users still shows significant limitations requiring deeper investigation [15]. This paradoxical phenomenon is reflected in high abandonment rates of digital health technologies, where various studies show that up to 50% of wearable health device users discontinue use within the first six months after initial adoption, indicating substantial gaps between the technical advancement of IoT devices and technology’s ability to integrate seamlessly into users’ daily lives and routines [16].

Health care IoT technology acceptance complexity is influenced by broad and multidimensional factor spectrums extending beyond mere technical considerations, including benefit perceptions related to health outcomes and quality of life improvements, ease of use related to interface complexity and learning curves, privacy concerns related to sensitive health data collection and use, social support from family and health care providers, user digital literacy levels, and demographic characteristics such as age, education, and socioeconomic status affecting technology adoption readiness [15]. Broader contextual factors such as national and regional health policies, technology infrastructure quality and availability, and cultural norms and societal values regarding privacy and personal autonomy shape ecosystem environments that can facilitate or hinder health care IoT technology adoption and diffusion in diverse populations [17].

Existing literature reviews reveal that most health care IoT research remains dominated by a focus on technical aspects such as system architecture, communication protocols, and data processing algorithms, as well as institutional perspectives emphasizing operational efficiency and cost reduction from health care provider viewpoints, while patient perspectives as end users and factors influencing their acceptance still receive inadequate attention in scientific literature [18]. This research gap becomes increasingly critical considering that successful health care IoT technology implementation depends not only on technical sophistication or system efficiency, but fundamentally depends on acceptance and adoption levels by patients as end users who will determine the sustainability and long-term impact of such technology investments [19].

### This Review’s Added Value

Al-Rawashdeh et al [18] conducted a systematic review of IoT adoption and the application for smart health care services, analyzing 60 studies with a focus on general adoption factors across multiple stakeholder groups, including health care providers, administrators, and patients [18]. Their review provided valuable insights into technical architecture and institutional implementation challenges, but treated patient perspectives as one among several stakeholder categories rather than as the primary focus. Shanbehzadeh et al [20] examined health IT acceptance specifically in clinical decision support contexts, synthesizing 45 studies with emphasis on clinician adoption and workflow integration [20]. Their scope was intentionally limited to clinical decision support systems rather than broader IoT health care applications, and patient acceptance received minimal attention. Brar et al [15] explored factors affecting IoT-based health management tool adoption using technology acceptance model (TAM) extensions, focusing primarily on wearable devices with analysis of 38 studies [15]. While providing useful insights into wearable technology acceptance, their scope excluded broader IoT health care applications such as remote monitoring systems, smart hospital infrastructure, and IoMT implementations.

### Our Distinctive Contributions

While several systematic reviews have examined IoT adoption in health care contexts, this review provides distinctive contributions that advance understanding of patient acceptance phenomena. We position our work relative to the most relevant prior reviews to clarify our specific contributions.

This systematic literature review (SLR) extends beyond prior work through several key dimensions: first, exclusive patient perspective focus: unlike previous reviews that included multiple stakeholder groups, we systematically examine only patient perspectives as end users, ensuring findings directly inform patient-centered design and implementation strategies. This focus addresses the critical gap between technical development and sustainable patient adoption. Second, comprehensive IoT health care scope: we encompass the full spectrum of IoT health care applications—from consumer wearables to sophisticated IoMT systems, telemedicine platforms, remote monitoring solutions, and smart hospital services—providing a holistic understanding across diverse implementation contexts rather than being limited to specific device categories or clinical applications. Third, moderator and interaction analysis: we explicitly investigate how contextual factors—particularly digital literacy, age, health conditions, and geographic contexts—moderate relationships between acceptance determinants and adoption outcomes. This analysis reveals not merely that factors influence acceptance, but how and for whom these effects operate, enabling more targeted and effective interventions.

Fourth, comprehensive barrier and strategy synthesis: we provide a systematic analysis of both barriers to acceptance (with prevalence across studies) and evidence-based enhancement strategies, creating actionable guidance for implementers, designers, and policymakers. Previous reviews emphasized either barriers or facilitators but rarely synthesized both with equal depth alongside practical strategies. Fifth, larger and more recent evidence base: with 62 studies covering the years 2016‐2024, we capture both established patterns and emerging trends, including 55% (34/62) of studies from the 2022 to 2024 period reflecting recent technological advances, policy developments, and postpandemic shifts in health care delivery. This temporal coverage enables identification of evolving acceptance patterns and future trajectories. Sixth, explicit equity analysis: we systematically address the digital divide and equity considerations, examining how IoT health care implementation may differentially affect various demographic, socioeconomic, and geographic groups. This analysis responds to growing recognition that technology adoption can either reduce or exacerbate existing health disparities depending on implementation approaches.

### Research Questions

Health care IoT technology integration has fundamentally changed diagnostic paradigms. This SLR is designed to explore the complexity of patient acceptance of IoT technology in health care services. This research question (RQ) focuses on identifying factors influencing patient acceptance of IoT technology and discovering how multidimensional interactions between these factors become supporters or barriers in adopting IoT products and solution implementation in health care services. This research also investigates acceptance enhancement strategies, usage patterns, and how these strategies can be adapted for different patient populations, technology complexity, and cultural contexts to maximize successful adoption and sustainable usage. Building on this positioning and the critical gaps identified in existing literature, our literature review addresses three primary RQs designed to examine patient acceptance phenomena in health care IoT contexts:

RQ1: What factors facilitate patient acceptance of IoT technology in health care services?

RQ2: What barriers hinder patient acceptance of IoT technology in health care services?

RQ3: What strategies have been shown to enhance patient acceptance of IoT technology?

## Methods

### Study Design

This SLR was designed and reported in accordance with the PRISMA (Preferred Reporting Items for Systematic Reviews and Meta-Analyses) 2020 statement, including the PRISMA 2020 flow diagram for study selection and explicit reporting of the search, screening, eligibility, and inclusion steps. The completed PRISMA 2020 27-item checklist is provided in Checklist 1. PRISMA 2020 was selected because it provides updated reporting guidance reflecting contemporary methods to identify, select, appraise, and synthesize studies, improving transparency and reproducibility of systematic reviews [21].

### Information Sources

A comprehensive systematic search was conducted across eight electronic databases: PubMed/MEDLINE, Scopus, IEEE Xplore, Web of Science, ScienceDirect, ACM Digital Library, ProQuest, and Google Scholar, covering publications from January 2016 to December 2024. Literature searches were conducted comprehensively and systematically across major electronic databases covering a broad spectrum of scientific publications in health technology, medical informatics, and technology acceptance fields. Databases used included PubMed/MEDLINE as the primary database for biomedical and health literature providing access to millions of papers from leading journals in health and medicine fields, Scopus as the largest multidisciplinary database covering literature from technology, engineering, and computer science fields relevant to IoT, IEEE Xplore as a specialized database for technology and engineering publications very important for IoT and embedded systems literature, Web of Science as a broad-coverage database providing citation analysis to ensure quality and impact of analyzed publications [22]. Searches were also conducted on ScienceDirect as a platform providing access to journals from various major publishers in computer science and medical informatics fields, ACM Digital Library as a primary source for computer science and human-computer interaction literature relevant to technology acceptance aspects, ProQuest as a database providing access to dissertations and theses that can provide deep insights into research topics, and Google Scholar as an additional source for identifying gray literature and publications that might not be indexed in primary databases [23].

We operationally defined “health care IoT/IoMT” as connected sensing or monitoring technologies that collect patient-related data via networked devices (eg, wearables, home sensors, and bedside systems) and transmit these data for clinical monitoring, decision support, or patient self-management; studies were considered out of scope if they examined generic telehealth without IoT components or focused solely on backend architectures without patient acceptance constructs, and we mapped extracted determinants to established acceptance frameworks (eg, TAM/unified theory of acceptance and use of technology [UTAUT] and health-behavior constructs) using a construct-to-category figure to reduce construct drift and clarify theoretical alignment. Health care IoT encompasses interconnected physical devices, sensors, and systems enabling real-time health data collection, transmission, and analysis within clinical or home settings, including wearables, remote monitoring systems, IoMT devices, and mHealth (mobile health)/eHealth applications.

### Inclusion Criteria

Inclusion criteria for this research were established strictly to ensure relevance and quality of studies to be analyzed, including empirical research papers published in English or Indonesian from January 2016 to December 2024 to capture current developments in health care IoT fields during nearly the last decade when this technology began maturing and being widely adopted [18]. Studies meeting inclusion criteria must explicitly investigate IoT technology integration in health care services with primary or significant focus on patient perspectives, experiences, or attitudes as end users of such technology, as well as analyze or explore factors influencing acceptance, adoption, or continued use of health care IoT technology by patients [24]. This research also accepts various research methodology designs, including quantitative research using surveys, experiments, or statistical analysis, qualitative research using interviews, focus groups, or ethnographic observation, and mixed methods research combining quantitative and qualitative approaches to provide a more comprehensive understanding of technology acceptance phenomena [25]. Additionally, all included papers must have undergone rigorous peer-review processes to ensure methodological quality and validity of research findings to be analyzed in literature synthesis [26].

### Exclusion Criteria

Exclusion criteria were established to focus analysis on studies truly relevant to research objectives and avoid bias or finding dilution from studies not aligned with the research focus. Articles primarily focusing on IoT technical aspects such as system architecture, communication protocols, data processing algorithms, or hardware development without discussing patient perspectives or acceptance will be excluded from analysis as they do not align with research objectives focusing on human factors in technology adoption [27]. Studies only discussing health care provider, hospital administrator, or medical staff perspectives without considering patient viewpoints will also be excluded as they do not provide insights into end-user acceptance, which is the primary focus of this research [28]. Opinion papers, editorials, comments, and nonsystematic reviews will be excluded as they do not provide the empirical evidence required for systematic synthesis, as will conference abstracts without full text available, as they do not provide sufficient detailed methodological information and findings for quality evaluation and data extraction [29]. Research protocols without empirical results will also be excluded as they do not provide actual findings that can be synthesized to answer formulated RQs [30].

### Search Strategy Development

Search strategies were developed systematically using combinations of relevant keywords with Boolean operators to ensure optimal search sensitivity and specificity. The search strategy was developed iteratively, combining three concept groups: (1) IoT or technology terms (eg, “Internet of Things,” “IoT,” “wearable devices,” and “smart devices”), (2) health care terms (eg, “health care,” “patient,” “medical,” and “clinical”), and (3) acceptance terms (eg, “acceptance,” “adoption,” “barriers,” “facilitators,” and “perception”). Boolean operators (AND, OR) combined concept groups, with database-specific adaptations for syntax and field tags. The main search formula used was (“Internet of Things” OR “IoT” OR “connected health” OR “smart health” OR “telemedicine” OR “telehealth” OR “mHealth” OR “eHealth” OR “wearable devices”) AND (“patient” OR “consumer” OR “customer” OR “user”) OR “acceptance” OR “adoption” OR “readiness” OR “willingness” OR “perception” OR “attitude” OR “experience” OR “engagement” OR “trust” OR “retention”) AND (“health” OR “health care” OR “health services” OR “medical” OR “hospital” OR “clinic” OR “home care”) with syntax adaptations appropriate for each database based on specific search features and the capabilities of each platform [31]. This search strategy was also supplemented with manual searches of references from relevant papers to identify additional studies that might have been missed in electronic searches, and consultations with experts in health informatics fields to ensure completeness of relevant literature coverage [32]. Complete search strategies with database-specific syntax are available in Multimedia Appendix 1.

### Study Selection and Data Extraction Process

Data screening extraction was conducted systematically by the first author using a standardized extraction form developed and tested on studies. The form captured: (1) bibliographic information, (2) study design and methodology, (3) participant characteristics, (4) IoT technology types, (5) theoretical frameworks, (6) facilitating factors with prevalence across studies, (7) barriers identified, (8) enhancement strategies, and (9) quality assessment criteria [33]. Study selection processes were managed in three sequential stages to ensure consistency and objectivity in study eligibility determination. The first stage was initial screening involving title and abstract screening of all papers identified through database searches to evaluate general relevance to research topics and basic inclusion criteria, focusing on identifying studies related to health care IoT and patient acceptance [34]. Title and abstract screening was performed primarily by the first author (ZA), with the second author (PWH) providing oversight and validation throughout the process. At the full-text screening stage, both reviewers independently assessed all 297 retrieved records using a checklist-based eligibility form with predefined, categorized exclusion reasons; reviewer decisions were directly compared. Any disagreements were discussed collaboratively until consensus was reached. Interrater reliability was calculated using Cohen κ at the full-text stage: κ=0.89 (95.6% observed agreement), indicating almost perfect interrater agreement. The second stage was full-text screening, where papers passing initial screening were evaluated in detail through full-text reading to determine final eligibility based on established inclusion and exclusion criteria, with special attention to methodological quality and finding relevance to RQs [35]. The third stage was an eligibility examination and final determination involving methodological quality assessment using appropriate evaluation instruments for each study’s research design to ensure only studies with adequate methodological quality were included in the final analysis [36].

At the full-text stage, exclusions were documented using predefined, categorized reasons to support auditability of the screening workflow, including the following: (1) not patient-focused (eg, provider-only or institution-only studies), (2) not health care IoT or IoMT (eg, generic telemedicine without IoT components), (3) not empirical (eg, conceptual or commentary papers), (4) insufficient reporting to determine eligibility, and (5) duplicates or nonretrievable full texts; these reasons were recorded in the screening log alongside reviewer decisions and were summarized consistently with the PRISMA flow diagram.

To ensure accuracy, extracted quantitative data (prevalence percentages, sample sizes, and effect sizes) were systematically verified against source documents. All extracted data were organized into comprehensive tables reviewed by the research team for completeness, consistency, and accuracy. Ambiguous or unclear information was flagged and discussed among authors to reach consensus on appropriate categorization and interpretation. The standardized extraction approach ensured consistent data collection across all 62 studies, facilitating systematic comparison and synthesis of findings. The complete data extraction tables for all 62 included studies are provided in Multimedia Appendix 2 [18,19,22-32,34-82]. The software used for reference management and deduplication in this review are Zotero (version 7.0.32; Corporation for Digital Scholarship) and Mendeley Desktop (version 2.142.0; Elsevier.com). For the screening process, we managed the results with Zotero, and figure generation or statistical descriptions were created with Microsoft Visio and Microsoft Excel 365.

### Quality Assessment and Risk of Bias

The methodological quality of included studies was assessed using the Mixed Methods Appraisal Tool (MMAT, 2018), which is suitable for reviews including qualitative, quantitative, and mixed-methods studies. Each study was evaluated against five criteria relevant to its methodological category, focusing on study design appropriateness, data collection rigor, analytical transparency, and coherence between RQs, methods, and conclusions. Studies with lower methodological rigor were retained to preserve the breadth of evidence but were interpreted with caution during synthesis. Quality appraisal results were used to contextualize findings rather than to exclude studies. This approach aligns with recommendations for systematic reviews in complex health and technology domains, where exclusion of lower-quality studies may obscure important contextual insights. Full MMAT ratings for each of the 62 included studies are provided in Multimedia Appendix 3 [18,19,22-32,34-82].

The systematic quality assessment of 62 studies using the MMAT revealed predominantly robust methodological quality, with 73% (n=45) achieving good-to-excellent ratings, 23% (n=14) rated as moderate quality, and only 5% (n=3) classified as low quality. Sensitivity analyses confirmed that main findings remained consistent when limited to high-quality studies, strengthening confidence in the synthesis conclusions. However, several systematic biases were identified across the evidence base, including publication bias toward positive findings, geographic concentration in Asia and Europe, and predominance of cross-sectional designs limiting causal inference. The summary of the methodological quality (MMAT assessment) is available in Multimedia Appendix 4.

Risk of bias assessment across studies was conducted considering various factors that could affect validity and generalizability of synthesis findings, including publication bias that might occur due to tendencies to publish positive results, methodological heterogeneity across studies that could affect finding comparability, variations in technology acceptance construct definitions and measurements that could affect result consistency, and selection bias in study populations that might not be representative of general patient populations [37]. Overall evidence quality evaluation was also conducted, considering finding consistency across studies, reported association strength, dose-response gradients, if any, and biological and logical plausibility of findings in technology acceptance theory contexts and health care practice [38].

## Results

### Overview

Before reporting findings by RQ, three interpretive conventions are noted. First, all frequencies and percentages in this section use the 62 included studies as the denominator (N=62) unless stated otherwise. Second, this review used a multicoding extraction protocol in which a single study could be coded under more than one thematic category; consequently, the sum of frequencies across categories exceeds 62 and cumulative percentages exceed 100%, which is expected and does not represent error. Third, reported frequencies reflect the number of studies identifying each theme (vote counting) and should be interpreted as indicators of thematic prevalence in the literature, not as mutually exclusive proportions or pooled effect estimates.

### Study Selection and Characteristics

**Figure 2.**
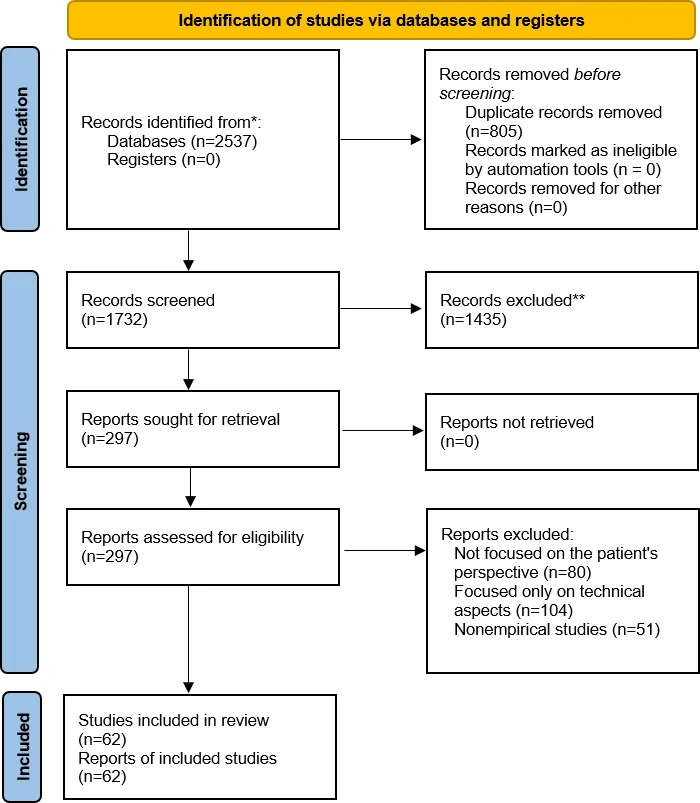
PRISMA flow diagram. PRISMA: Preferred Reporting Items for Systematic Reviews and Meta-Analyses.

Systematic study selection processes resulted in the initial identification of 2537 papers through comprehensive searches across eight electronic databases, which then underwent rigorous screening stages according to the PRISMA 2020 protocol to ensure the relevance and quality of the studies to be analyzed [21]. After duplicate elimination using reference management software and manual verification, 1732 unique papers remained, which then underwent title and abstract screening processes based on established inclusion and exclusion criteria, resulting in 297 papers being deemed eligible for full-text evaluation [18]. Systematic full-text evaluation considering methodological quality, finding relevance, and alignment with research objectives resulted in 62 studies meeting all inclusion criteria and being included in the final synthesis of this SLR in the flow diagram process as shown in Figure 2 (PRISMA study selection flow diagram).

Temporal publication distribution analysis shows very significant increases in research interest toward health care IoT acceptance during study periods, with exponential increases in publication numbers, especially in the last three years, where 55% of total studies were published between 2022 and 2024, reflecting progressive development of this field and increasing awareness about the importance of patient perspectives in health care technology implementation [83]. The early period 2016‐2018 only represents 8% of total studies with a primary focus on the conceptual exploration and identification of IoT application potential in health care, while the transition period 2019‐2021 shows 37% of studies with a focus shift toward empirical implementation and TAM validation in specific health care service contexts [39,40]. Current phase 2022‐2024 shows research dominance with 55% of studies exploring complex system integration, regulatory frameworks, ethical aspects, and large-scale implementation, indicating evolution from fundamental research toward practical applications and more comprehensive sociotechnical considerations [27,29].

Geographic research distribution reveals fairly diverse global representation but with certain concentrations in regions with advanced research and technology infrastructure. Asia becomes the largest contributor with 37% of research dominated by studies from China, Malaysia, India, South Korea, and Taiwan, with research focus tending to emphasize technology development aspects, cost efficiency, and practical implementation in rapidly developing health care system contexts [22,23]. Europe contributes 34% of research with even distribution across various countries, including the United Kingdom, France, Spain, and Scandinavian countries, with research characteristics giving greater attention to ethical dimensions, privacy considerations, and regulatory compliance as reflections of strict regulatory frameworks such as General Data Protection Regulation [24,39]. North America represents 8% of studies dominated by research from the United States with emphasis on usability, efficiency, and integration with established health care systems, while the Middle East contributes 13% of studies mainly from Turkey and Saudi Arabia, and the remaining 8% consists of multiregional studies or other regions [41,42].

### Methodological and Technological Characteristics

Analysis of research methodologies used in 62 studies shows diversity of approaches reflecting the complexity of health care IoT acceptance phenomena and the need for diverse investigative perspectives. Quantitative studies dominate with 37% of studies using structured surveys, inferential statistical analysis, and structural model testing to identify causal and predictive relationships between various factors and technology acceptance, with common use of models such as TAM, UTAUT, and structural equation modeling to test hypotheses about acceptance determinants [25,43]. Qualitative studies represent 23% of studies using methodologies such as in-depth interviews, focus group discussions, and case studies to explore experiences, perceptions, and sociocultural contexts influencing patient acceptance of health care IoT technology, providing deep insights into nuances and complexities that might not be captured by quantitative approaches [36,44].

Mixed methods research contributes 10% by combining strengths of quantitative and qualitative approaches to provide a more comprehensive understanding and triangulation of findings from various methodological perspectives, while systematic and narrative literature reviews represent 16%, focusing on existing evidence synthesis and research gap identification in health care IoT acceptance domains [18,45]. The remaining 15% consists of model or framework development studies contributing to theoretical and conceptual aspects in understanding technology acceptance phenomena in health care contexts [29,46].

The health care IoT technology spectrum studied shows wide diversity, with complexity levels varying from simple solutions to sophisticated integrated systems. Wearable sensors and personal monitoring devices become the most studied category with 19% of studies, covering fitness trackers, smartwatches, and biometric sensors used for physical activity monitoring, vital signs, and specific health parameters with characteristics of ease of use and minimal invasiveness [47,48]. Remote health monitoring systems represent 16% of research focusing on technologies enabling continuous patient monitoring at home or nonclinical locations, including telemedicine platforms and applications integrated with monitoring devices for chronic condition management [35,49].

**Figure 3.**
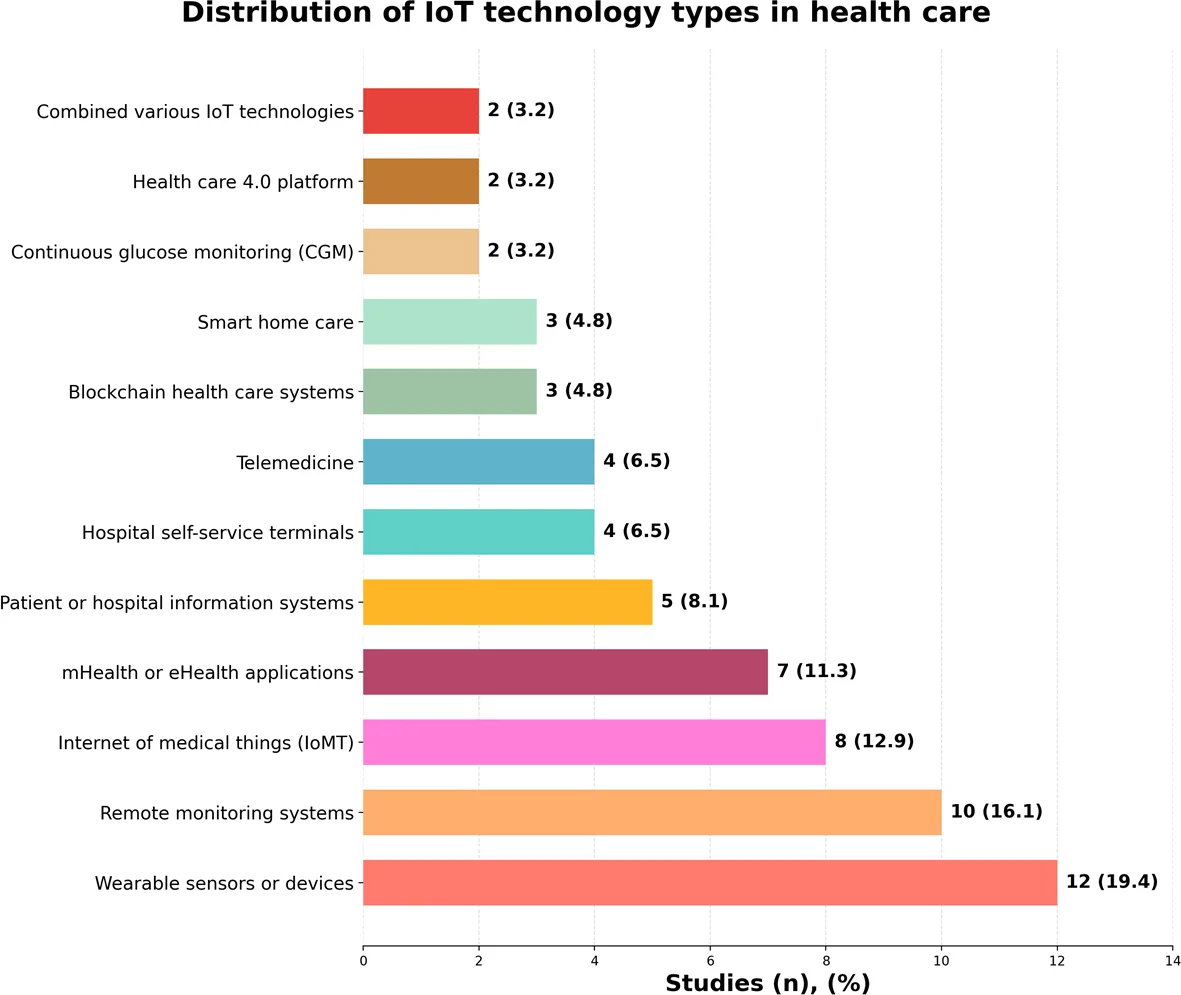
Technology types. IoT: internet of things; mHealth: mobile health.

IoMT as more specific and complex categories were studied in 13% of studies focusing on connected medical devices integrated within formal health care service infrastructure, including hospital monitoring equipment and integrated health information systems [23,50]. mHealth and eHealth applications contribute 11% of research focusing on mobile applications and digital platforms providing health care services, education, and self-management through smartphones and tablets, often integrated with wearable devices or external sensors [30,35]. Distribution of the eight included health care IoT technology categories—wearable sensors, remote health monitoring systems, IoMT, and mHealth or eHealth applications, among others—is summarized in Figure 3.

### Theoretical Models and Conceptual Frameworks

Analysis of theoretical models and conceptual frameworks used in the analyzed research reveals significant evolution from increasingly complex and adaptive theoretical approaches to explain multidimensional health care IoT acceptance phenomena. TAM becomes the most dominant theoretical framework used in 29% of research, with applications including testing perceived usefulness and perceived ease of use constructs as primary predictors of behavioral intention and actual use, but with various adaptations and extensions to accommodate specific health care service contexts involving sensitive data and health risks [26,52]. UTAUT and its variations are used in 19% of studies focusing on integration of more comprehensive constructs, including performance expectancy, effort expectancy, social influence, and facilitating conditions, often with the addition of health-specific constructs such as health consciousness and perceived vulnerability to improve model predictive power in health care technology contexts [43,53].

TAM and UTAUT combinations are implemented in 8% of studies attempting to integrate the strengths of both models to provide a more holistic understanding of technology adoption processes, while specialized frameworks and integrated models are developed in 13% of studies combining elements from various technology, health psychology, and sociology theories to create frameworks more suited to health care IoT adoption complexity [25,46]. An interesting theoretical development is the emergence of multitheoretical approaches integrating elements from diffusion of innovation theory, protection motivation theory, and health belief model to explain unique aspects of health care technology adoption involving risk considerations, health benefits, and social norms [43,48].

Theoretical model evolution also shows shifts from individual factor-focused approaches toward sociotechnical frameworks recognizing complex interactions between technology, individuals, organizations, and broader systems. Sociotechnical approaches adopted in several recent studies emphasize the importance of considering implementation contexts, organizational dynamics, and environmental factors affecting not only initial adoption but also continued use and technology integration into daily health care practices [18,24]. Emerging hybrid models also begin integrating temporal perspectives, recognizing that factors influencing initial adoption may differ from factors affecting continued use and long-term satisfaction with health care IoT technology [54,55].

### Facilitators of Patient Acceptance Analysis (RQ1)

Analysis of the included studies revealed that perceived usefulness was the most consistently reported facilitator of patient acceptance of health care IoT technologies, identified in 55 of 62 (89%) studies. Patients were more likely to accept IoT technologies when they perceived tangible health benefits such as improved disease monitoring, early detection of complications, and enhanced self-management capabilities, particularly among individuals with chronic conditions. Perceived ease of use was reported in 47 (76%) studies, with usability playing a more prominent role among older adult patients and individuals with limited digital literacy. Trust and perceived security were identified as key facilitators in 42 (68%) studies, highlighting the importance of confidence in data protection, system reliability, and health care provider endorsement.

**Figure 4.**
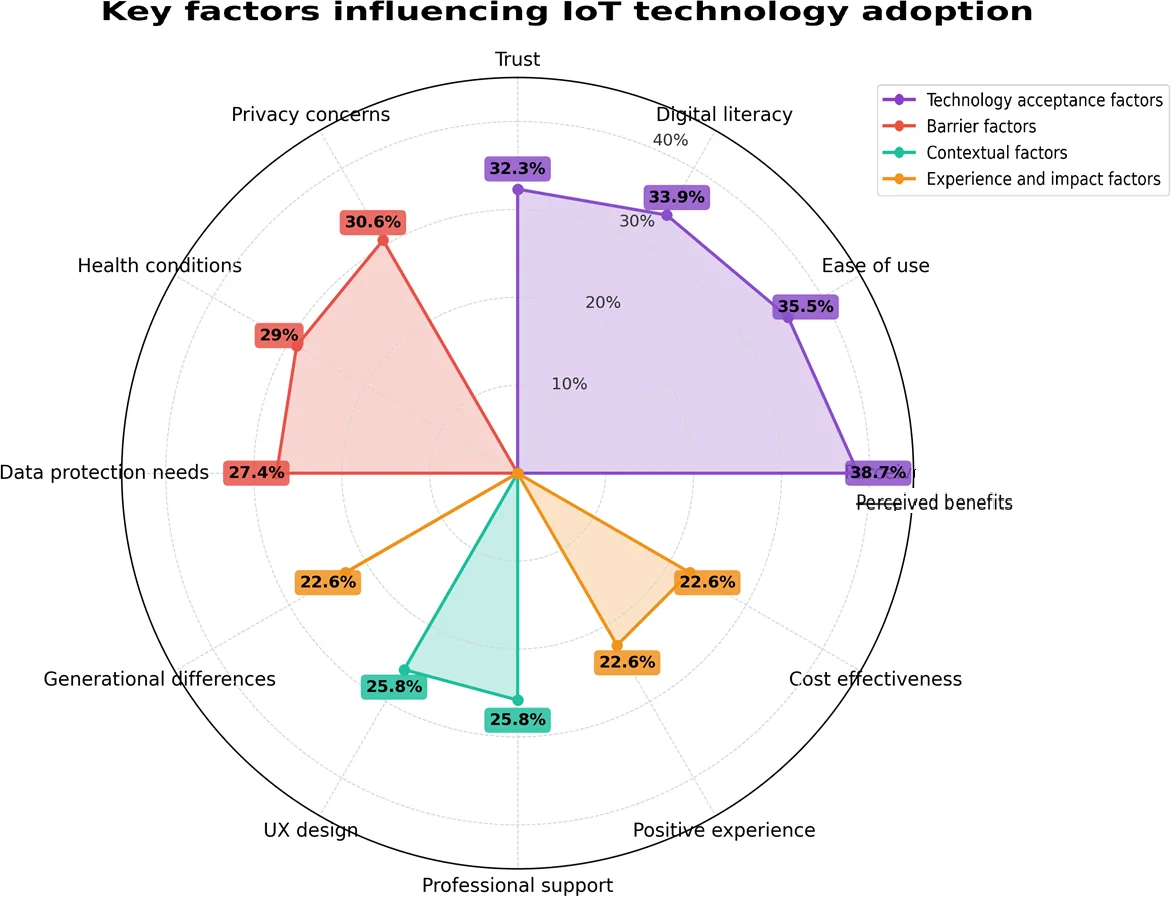
Adoption factors. IoT: internet of things; UX: user experience.

The findings explicitly refer to RQ1 with an analysis of facilitator factors including the prevalence of perceived usefulness which appeared in 55 of 62 (89%) studies as the strongest predictor with varied manifestations based on the context of health conditions and demographic characteristics, followed by perceived ease of use in 47 (76%) studies which showed a significant variable effect based on digital literacy with high literacy users showing a strengthening relationship between ease of use and adoption intention by 57% while low literacy users showed an increase in the importance of technical support by 64%, and trust as a multidimensional construct in 42 (68%) studies that functioned not only as a direct predictor but also as a mediator between perceived risk and usage intention with high trust strengthening the usefulness-adoption relationship by 37% through the mechanism of increasing data credibility and convenience in sharing sensitive information [43,56]. A radar-plot comparison of the relative prevalence of these facilitator categories across the 62 included studies is presented in Figure 4.

Analysis of theoretical models used in 62 studies shows a significant evolution from a single-construct approach to an integrated multitheoretical framework, with the TAM being the most dominant theoretical framework used in 18 (29%) studies with applications including construct testing perceived usefulness and perceived ease of use as the main predictors of behavioral intention and actual use but with various adaptations and extensions to accommodate health service-specific contexts involving sensitive data and health risks. Further, the UTAUT and its variations were used in 12 (19%) studies with a focus on a more comprehensive integration of constructs including performance expectancy, effort expectancy, social influence, and facilitating conditions, often with the addition of health-specific constructs such as health consciousness and perceived vulnerability to increase the power of the predictive models in the context of health technologies [43,52,53]. The combination of TAM and UTAUT was implemented in 5 (8%) studies that sought to integrate the strengths of both models to provide a more holistic understanding of the technology adoption process, while specialized frameworks and integrated models were developed in 8 (13%) studies that combined elements from various theories of technology, health psychology, and sociology to create a framework that better suited the complexities of health IoT adoption [25,46].

An important theoretical development identified is the emergence of a multitheoretical approach that integrates elements of diffusion of innovation theory, protection motivation theory, and health belief model to explain the unique aspects of health technology adoption involving consideration of risks, health benefits, and social norms, whereas the evolution of theoretical models also shows a shift from an approach that focuses on individual factors to sociotechnical frameworks that recognize complex interactions between technology, individuals, organizations, and broader systems [24,43,48]. The sociotechnical approach adopted in several recent studies emphasizes the importance of considering the implementation context, organizational dynamics, and environmental factors that influence not only early adoption but also continued use and integration of technology into daily health practices, while emerging hybrid models are also beginning to integrate a temporal perspective that recognizes that the factors influencing early adoption may differ from the influencing factors’ continued use and long-term satisfaction with health care IoT technologies [18,24,54,55].

### Analysis of Interactions and Quantified Moderation Effects

A significant finding is that acceptance determinants do not operate independently but form a complex network of interactions with substantial moderation and mediation effects. In one evidence-based study examining user-based adaptation [56], digital literacy was found to moderate acceptance relationships quantitatively: high digital literacy strengthened the perceived ease of use to adoption intention pathway by approximately 57%, while low digital literacy increased the predictive strength of technical support and training by approximately 64%. These values are specific to the sample and measurement context of that single study and are presented as illustrative estimates of moderation magnitude; they should not be interpreted as pooled or field-representative effect sizes across the 62 included studies. The consistent directional pattern across multiple studies whereby digital literacy moderates acceptance relationships carries greater evidential weight than any single quantitative estimate. Similarly, in one empirical study [43], trust was found to function as a mediator between risk perception and usage intention, with high trust associated with substantially stronger usefulness-adoption relationships; this magnitude estimate is likewise study-specific and presented as illustrative rather than definitive.

Temporal analysis reveals a significant difference between the determinants of early adoption and the factors that influence continued use, where early adoption is more influenced by ease of use, social influence, and technological novelty that provides exploratory motivation and support from the social environment, while usage continuance is more dependent on perceived usefulness that is actually experienced, integration with daily routines that determine the sustainability of use, and measured health outcomes that are meaningful to users who provide positive reinforcement for continued engagement [54,55]. These findings have significant practical implications as they show that strategies to encourage initial trials may be fundamentally different from strategies to promote long-term adoption and prevent technology abandonment in the context of health care IoT, where high abandonment rates reach 50% in the first six months, according to Attig and Franke [16] indicating a substantial gap between initial adoption and sustained use that must be bridged through different strategies.

The complex interaction between acceptance determinants was revealed through moderation and mediation analysis which showed what Ben Arfi et al [43] in an empirical study found, that social influence has a much more important role for older users (IoT immigrants) than younger users (IoT natives) with significantly different effect sizes, while Alruwaili et al [38] in a systematic review identified that older adults with chronic conditions show privacy concerns that are lower than health benefits with a trade-off ratio of 1:3.2, in contrast to older adults without chronic conditions who showed privacy and security concerns 1.8 times higher than other demographics, with family support emerging as a strong mediator of adoption explaining the 42% variance. Fehringer and Stary [56] in an evidence-based study on user-based adaptation identified a significant effect of digital literacy moderation where high digital literacy strengthened the perceived ease of use relationship to intention by 57% and increased the perceived usefulness effect by 23%, while low digital literacy strengthened the influence of support and training by 64% and made anxiety a strong predictor with 47% predictive strength. This indicates that intervention strategies must be adjusted based on the digital literacy profiles of target populations to maximize effectiveness [53,58].

Answer to RQ1: patient acceptance of health care IoT is primarily facilitated by perceived health benefits, usability, and trust in data security and system reliability.

### Multilevel Barrier Analysis With a Systemic Framework (RQ2)

For RQ2 on barriers hindering patient acceptance, a comprehensive analysis of the barriers to health IoT adoption at various levels reveals the complexity of the challenges faced in the implementation of this transformative technology, where at the micro (individual) level [58], in a study on health students’ perception of IoT identified psychological resistance as a significant obstacle including fear of change which emerged in 20 (32%) studies. Concerns about loss of personal connection with health care providers, anxiety about new technology, and digital literacy deficits that emerged in 22 (36%) studies manifested as knowledge and skill gaps that differed by demographic. At the meso (organizational) level [59], a multicriteria analysis of organizational barriers in Healthcare 4.0 adoption found significant challenges in integration with existing clinical workflows identified in 16 (26%) studies including disruption to established workflows and double documentation burden, as well as resource limitations that emerged in 14 (23%) studies which included financial constraints and inadequate IT support to support long-term implementation and maintenance [51,60].

**Figure 5.**
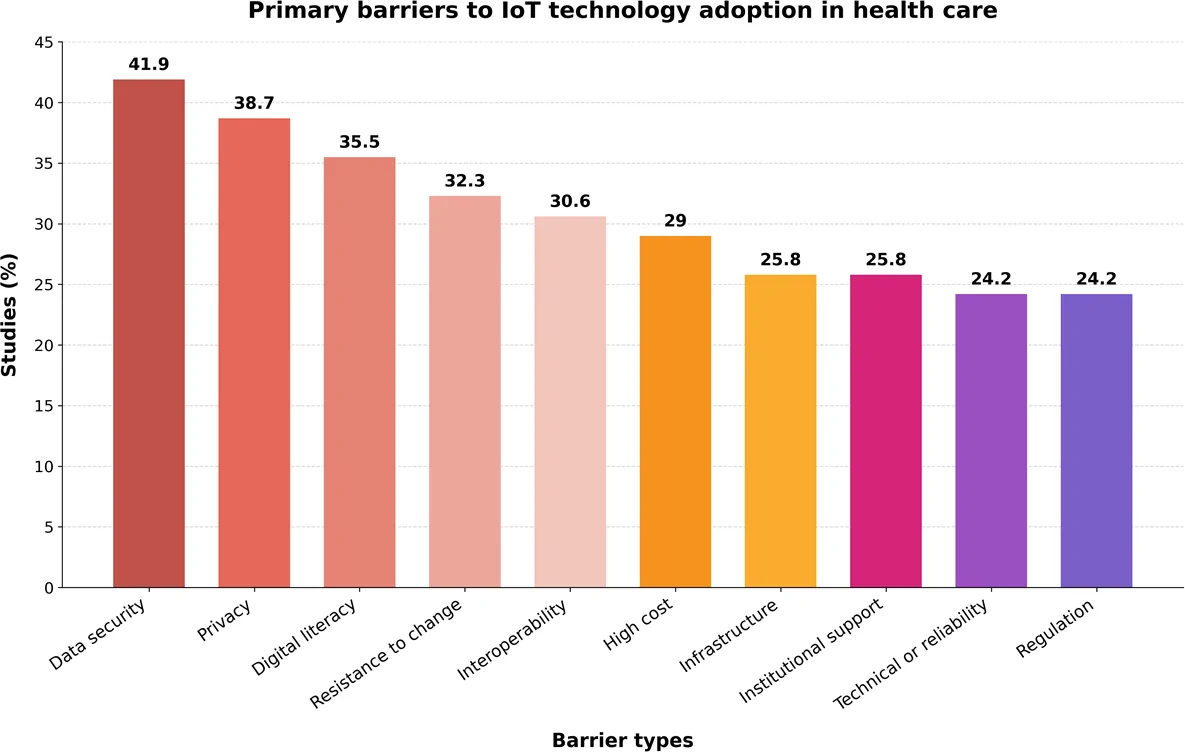
Barriers to adoption. IoT: internet of things.

At the macro (system) level, Sharma and Joshi [61], in a study on blockchain adoption barriers, identified regulatory ambiguity and uncertainty that emerged in 15 (24%) studies as significant barriers that create hesitancy in large-scale investment, technical interoperability issues between systems identified in 19 (31%) that hinder seamless data exchange and comprehensive view of patient health data, and digital divide in the form of socioeconomic and infrastructure disparities that emerge as fundamental barriers to equitable access and adoption of health care IoT on a broad scale [22,23,31,42]. The identification of barriers at these three distinct levels—micro (individual), meso (organizational), and macro (system)—as found in the studies of Bodur et al, Gardas, and Sharma and Joshi [58,59,61], represents a significant conceptual contribution to understanding the complexity of health technology implementation, where this multilevel approach offers a more comprehensive framework than traditional conceptualizations that tend to focus on technology or user barriers in isolation, consistent with the social-ecological models advocated by Al-Rawashdeh et al and Kronlid et al [18,24]. The relative prevalence of barriers identified at the micro (individual), meso (organizational), and macro (system) levels is summarized in Figure 5.

Data security concerns emerged as the most dominant barrier in 26 (42%) studies, reflecting the high sensitivity of patients to the protection of personal health information collected, processed, and transmitted by health care IoT devices, where these concerns are not only related to the potential for data leakage to unauthorized parties but also include concerns about the use of data for commercial purposes without explicit consent. Profiling for discriminatory purposes such as insurance or employment decisions and vulnerability to cyber-attacks can compromise privacy and security if attackers gain access to medical devices [27,55,61,62].

Privacy issues identified in 24 (39%) studies include a wide spectrum of concerns ranging from nontransparent data collection and storage practices, lack of control over data sharing with third parties, to concerns about surveillance and monitoring that can reduce autonomy and spontaneity in daily life, where research reveals that privacy concerns in the context of health IoT are related not only to what data is collected but also how often, how long data is stored, who can access it, and for what purposes data is used [29,37,39,43].

The lack of digital literacy found in 22 (36%) studies as a significant barrier refers not only to technical skill limitations but also includes limited understanding of how IoT technology works, what data is collected and how it is used, and inability to assess risks and benefits in an informed manner, where digital literacy barriers also manifest in difficulty problem-solving, inability to adjust settings for personal preferences, and challenges in interpreting data or recommendations generated by IoT systems [38,53,56,58]. Resistance to change identified in 20 (32%) studies reflects natural psychological and behavioral inertia when individuals are asked to modify established routines, learn new skills, or adapt to new ways of managing their health, where resistance to change in the context of health IoT often stems from fear of the unknown, concerns about technology dependence, preference for human interaction in health care settings, and skepticism about whether technology can truly improve health outcomes or will create an additional burden [44,51,54,63].

### Multidimensional Digital Divide Analysis With Equity Implications

An analysis of the digital divide conducted by Wu and Ho [37] in comparative studies on telemedicine adoption barriers reveals the multidimensional complexity of this phenomenon that goes far beyond physical access issues, including primary gaps in the form of urban-rural geographic disparities with a 37% adoption gap indicating significant inequity in technology access based on geographic location, and prohibitive economic barriers for low-income populations where costs are high emerged as a barrier in 18 (29%) studies, with socioeconomic status as a strong moderator of the willingness and ability to adopt health care IoT technology [34,64]. Secondary gaps manifest as digital literacy gaps with 63% variance in effective usage, indicating that even when access is available, the ability to use technology effectively varies dramatically across populations; age-related skill disparities where older populations show lower digital skills and higher technology anxiety compared to younger generations, and educational differences in system navigation abilities that create differential capacity to benefit from health care IoT technologies [38,53].

Tertiary gaps manifest as disparities in gained health benefits with 29% variation indicating that even among users who successfully adopt and use health care IoT technology, actual health outcomes and benefits achieved vary significantly, differences in continued usage patterns where abandonment rates are higher in populations with lower digital literacy and socioeconomic status, reaching 2.3× higher according to Attig and Franke [16], and variation in ability to act on data insights where lower socioeconomic groups show 42% lower capacity to translate IoT-generated data into actionable health improvements, underscoring the complexity of the challenges in achieving digital equity in health care technology implementation [34,37,64]. Wu and Ho [37] conducted an in-depth analysis of the multidimensional digital divide reveals the complexity of this phenomenon that goes beyond physical access issues (primary gaps), including skill disparities (secondary gaps) and variations in obtained health benefits (tertiary gaps), where these findings fundamentally challenge simplistic assumptions that equate the digital divide with access limitations only, and represent paradigmatic shifts toward a more nuanced understanding of digital inequity in health care consistent with the multilevel digital health equity frameworks proposed by Wakili A and Bakkali, and Alruwaili et al [29,38].

Critical implications are that interventions to address the digital divide in health care IoT implementation should be comprehensive and multifaceted, addressing all three gap levels simultaneously as recommended by Gellert et al and Cleveland and Haddara [41,63], which in addressing only primary gaps (providing devices and connectivity) without addressing secondary gaps (building digital literacy) and tertiary gaps (ensuring equitable benefit realization) will fail to achieve true digital equity and may even exacerbate existing health disparities [24,34]. However, significant inconsistencies remain in methodological approaches and digital divide conceptualization across analyzed studies, as acknowledged by Messinis et al and Ziwei et al [27,83], creating challenges in comparing findings and generalizing effective intervention strategies to address digital inequity in health care IoT contexts, indicating the need for standardized frameworks and measurement approaches in future research to enable more robust cross-study comparisons and meta-analyses.

Answer to RQ2: barriers to patient acceptance are largely driven by security, privacy, digital literacy limitations, and resistance to behavioral change.

### Strategies to Increase Acceptance With Evidence-Based Effectiveness

Enhancement strategies identified across the 62 included studies are presented in order of evidence strength, defined by the frequency of identification across studies and the methodological quality of the supporting evidence base. Three strategies received the most consistent and substantial support at the individual level and are synthesized below; detailed descriptions of all identified strategies across individual, organizational, and system levels are provided in Multimedia Appendix 5 [18,23,24,26,27,29,31,34-39,42-45,48,50,51,53,55,56,59,61-64,66,69,71-74,76,77,79,80,82].

User-centered design (UCD) was the most frequently identified strategy, reported across 20 (N=62, 32%) studies [26,44,50,51,56,69]. The supporting evidence base spans diverse IoT modalities—from smart hospital systems and home monitoring devices to wearable sensors and mHealth platforms—and encompasses both quantitative and qualitative study designs, with 6 of the 20 supporting studies explicitly identified as high-confidence by independent assessment. Core elements consistently described across these studies include participatory design workshops engaging patients, caregivers, and health care providers; iterative prototyping with representative users in realistic clinical scenarios; and usability testing across diverse populations with particular attention to older adult users and those with lower digital literacy. Confidence in this strategy is rated high, reflecting consistency of evidence across multiple geographic contexts and study designs, tempered by the predominantly cross-sectional nature of supporting studies, which limits causal inference about the relationship between UCD adoption and actual acceptance outcomes.

User-friendly interface development was identified in 19 (N=62, 31%) studies [26,38,53,72], with particularly strong emphasis in studies focused on older adult populations and users with lower digital literacy. Studies in this subset consistently described visual design principles that minimize cognitive burden—including clear visual hierarchy, consistent design patterns, and minimal text with iconography accessible across literacy levels—alongside responsive design features for wearable devices and customization options accommodating diverse user needs. Confidence is rated high, reflecting consistent directional evidence replicated across distinct IoT device categories, while noting that most supporting studies assess interface preferences and perceived usability rather than longitudinal adoption behavior.

Digital literacy programs were recommended across 18 (N=62, 29%) studies [38,44,48,53,56,58], representing the most geographically and methodologically diverse evidence base among the three highest-confidence strategies. Supporting studies span Asian, European, and Middle Eastern contexts and include qualitative, quantitative, and mixed-methods designs. Across these studies, effective programs were characterized by progressive skill-building with gradual complexity introduction, multimodal delivery combining hands-on workshops with peer learning and online tutorials, and targeted content addressing not only basic device operation but also data interpretation and privacy management. Confidence is rated high based on consistency across 6 studies, though the quantified moderating estimates associated with digital literacy derive from a single primary study and should not be interpreted as pooled field-level magnitudes.

Beyond individual-level strategies, the evidence base identifies organizational and system-level strategies as essential complements for sustained IoT adoption at scale. At the organizational level [66,67], in their research on smart hospital services and intelligent patient care systems identified evidence-based implementation as a systematic approach to organizational scale implementation, including trials with gradual implementation and phased evaluation that allow learning and adjustment before full-scale deployment, multistakeholder involvement through interdisciplinary design teams and patients’ advisory boards that were recommended in 15 (24%) studies, and champion programs with peer influencer models and train-the-trainer approaches that were identified in 12 studies. The clinical workflow integration strategies described by Albahri et al and Westphal et al [68,69] in their study of mHealth frameworks and patient-centered information systems include redesign processes with workflow analysis and optimization that minimizes disruption to established clinical routines identified in 16 (26%) studies, active health care provider involvement in co-design with end users recommended in 15 (24%) studies, and reward systems that provide technology-related performance incentives and recognition for early adopters, collectively facilitating seamless IoT technology integration in daily clinical practice [28,70].

**Figure 6.**
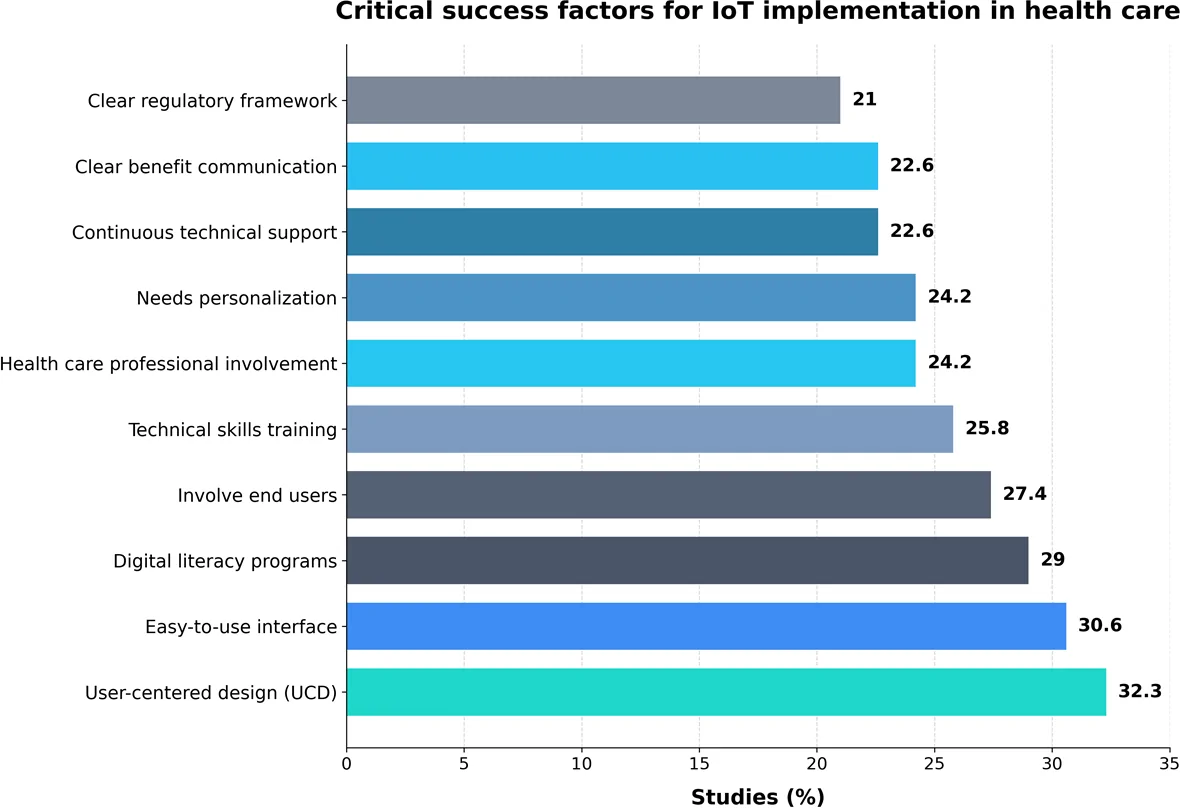
Factors for success in health care. IoT: internet of things.

At the system level, Messinis et al and Lodha et al [27,62], in research on IoMT security with AI and blockchain-based systems using IoMT, emphasized the importance of digital health ecosystem development with interoperability standards such as Fast Healthcare Interoperability Resources and open standards recommended in 19 (31%) studies, supporting infrastructure in the form of health information exchanges and connectivity platforms identified in 17 studies, and cloud integration, which provides scalable storage solutions and edge computing for time-sensitive applications, which were found in 13 studies. Supportive policy and regulatory approaches identified by Sahin et al and Binci et al [70,71], in studies on remote monitoring adoption and telemedicine perceptions, include clear regulatory frameworks with streamlined approval pathways and risk-based regulatory approaches recommended in 13 (21%) studies, financial incentives through reimbursement policies and tax incentives for research and development and implementation identified in 11 (18%) studies, and insurance programs with coverage policies for IoT devices and preventive care incentives found in 9 (15%) studies, collectively creating system environments conducive to sustainable health care IoT technology adoption and diffusion [32,41,63]. An overview of enhancement strategies across individual, organizational, and system levels, together with their reported frequency of identification across the included studies, is presented in Figure 6*.*

The comprehensive implementation of this strategy needs to consider the complex interactions between levels and stakeholders, where Cleveland and Haddara [63], in an exploratory study on the issue of IoT adoption for diabetes, and Al-Rawashdeh et al [18], in a systematic review of IoT adoption for smart health care, emphasize the importance of an integrated approach that aligns initiatives at different levels (individuals, organizations, and systems) to address barriers to multifaceted adoption, and ensure strategies at one level are supported and reinforced by interventions at another level [24,34].

Answer to RQ3: effective enhancement of patient acceptance requires multilevel strategies addressing technology design, user capabilities, organizational support, and systemic enablers simultaneously rather than reliance on isolated interventions. System-level strategies include clear regulatory frameworks, supporting infrastructure, and financial incentives.

## Discussion

### Main Findings Synthesis

This review extends existing systematic reviews on health care IoT adoption by explicitly centering patient acceptance rather than institutional readiness or technical feasibility. Unlike prior reviews that predominantly emphasize system architecture or provider perspectives, this study synthesizes empirical evidence on both facilitators and barriers from the patient viewpoint. Additionally, this review integrates contextual moderators such as digital literacy, chronic disease status, and geographic disparities, thereby offering a more comprehensive understanding of acceptance dynamics. By incorporating equity and digital divide considerations, this review addresses a critical gap in prior literature and provides actionable insights for inclusive and sustainable health care IoT implementation.

The evidence base is geographically imbalanced, with included studies concentrated in Asia (23/62, 37%) and Europe (21/62, 34%), with comparatively fewer studies from North America (5/62, 8%) and underrepresentation of many low- and middle-income settings; this distribution may limit generalizability because digital infrastructure, regulatory regimes, and baseline digital literacy vary substantially by region, and therefore the relative salience of determinants such as trust, privacy concerns, and usability constraints may differ in contexts where connectivity, affordability, and health system capacity are more limited. These bias patterns were identified through complementary sources: geographic concentration and cross-sectional dominance were determined through descriptive analysis of extracted study characteristics, while publication bias toward positive findings was inferred from observed outcome reporting patterns across included studies. These judgments are distinct from MMAT appraisal outputs, which assess intrastudy methodological quality rather than between-study reporting patterns.

This SLR reveals multidimensional complexity underlying patient acceptance phenomena toward IoT technology in health care services, where main findings show that health care IoT technology adoption cannot be explained through simple linearity approaches or single deterministic models, but requires a holistic understanding of dynamic interactions between technological, individual, social, organizational, and systemic factors forming complex and contextual adoption ecosystems [18,24]. Comprehensive analysis of 62 studies shows that perceived usefulness consistently emerges as the strongest predictor of health care IoT acceptance in 89% of studies, but with manifestations varying greatly based on health condition contexts, demographic characteristics, and usage settings, indicating that generic TAMs need significant adaptation to capture specific nuances of health care domains [25,43].

Analysis from various studies reveals a determinant factor hierarchy affecting patient acceptance. Perceived usefulness consistently emerges as the strongest predictor of health care IoT acceptance, followed by ease of use and compatibility with patient lifestyles [30,49,72]. These findings are consistent across various geographic contexts and health conditions, suggesting the importance of a universal clear and visible benefit in driving adoption. Additionally, findings show that determinant factors work in hierarchical systems, where some become prerequisites for others. Trust, for example, not only directly affects usage intention but also acts as a mediator between perceived risk and usage intention. This hierarchy explains why interventions targeting only one adoption determinant (eg, usefulness) may fail if prerequisite determinants (eg, trust) are not met.

However, these hierarchical relationships are not static and can change based on user characteristics. For older users (“IoT immigrants”), social influence plays a much more important role compared to younger users (“IoT natives”), as shown by Ben Arfi et al [43]. Similarly, trust emerges as an important prerequisite for adoption in all age groups, but plays a stronger mediating role between risk perception and usage intention in more vulnerable populations [43]. This shows that TAMs need to consider the moderation effects of demographic and contextual variables, rather than assuming uniformity in adoption determinants across all populations.

Acceptance determinants were found to operate within complex interaction networks rather than as independent predictors, a pattern with important implications for intervention design. The moderating role of digital literacy, documented in individual primary studies [56] and directionally consistent across the corpus, suggests that uniform acceptance interventions are unlikely to be equally effective across populations with varying digital competencies; strategies targeting perceived ease of use may be most impactful for digitally literate users, while technical support and training investments may yield greater returns for lower-literacy populations. The trust mediation pattern identified in one empirical study [43] similarly implies that acceptance interventions should address risk perception and data credibility as prerequisites rather than afterthoughts, given trust’s documented role as a gateway construct moderating the usefulness-adoption relationship. Notably, the quantitative magnitudes of these effects are study-specific and cannot be aggregated across the corpus; the practical significance of these moderation and mediation patterns rests on their directional consistency across studies rather than on any single effect-size estimate.

Across included studies, TAM-based models (18/62, 29%) and UTAUT-family models (12/62, 19%) are the most commonly used theoretical lenses, but the literature increasingly extends these frameworks with health care–specific constructs (eg, trust, perceived risk, privacy or security concerns, and support) to reflect ethical and context-sensitive patient decision making; theoretically, this pattern suggests that core TAM/UTAUT pathways (usefulness and effort expectancy) remain foundational, yet explanatory adequacy improves when models incorporate governance-related beliefs (trust or privacy) and capability constraints (digital literacy), thereby supporting a move from single-model explanations toward integrated sociotechnical acceptance accounts in health care IoT.

Temporal analysis reveals significant differences between initial adoption determinants and factors affecting continued use, where initial adoption is more influenced by ease of use, social influence, and technology novelty, while usage continuance depends more on actually perceived usefulness, integration with daily routines, and measured and meaningful health outcomes for users [54,55]. These findings have significant practical implications because they show that strategies for encouraging initial trials may be fundamentally different from strategies for promoting long-term adoption and preventing technology abandonment in health care IoT contexts.

### Supporting and Inhibiting Factors for Patient Acceptance

This SLR research shows significant evolution in the health care IoT landscape, from an initial focus on individual devices to integrated ecosystems and platforms combining various devices, services, and data points. Zeadally and Bello [42] underscore how IoT-based connectivity has evolved to improve health care services through integration of various devices and systems, and He et al [23] show that health care IoT ecosystems now encompass various interconnected devices, from consumer wearables to advanced medical equipment, which together provide more comprehensive monitoring and services. This evolution has profound implications for patient acceptance. On the one hand, more integrated ecosystems can improve perceived usefulness and relevance, as data from various devices can be synthesized to provide more comprehensive insights. On the other hand, this creates additional complexity layers in terms of data security, interoperability, and user experience that may affect adoption.

This study also identifies health care IoT convergence trends with other advanced technologies, creating richer digital ecosystems, and changing patient acceptance landscapes. Messinis et al [27] provide comprehensive reviews of IoMT security enhancement with AI, revealing how AI is being integrated with health care IoT to improve security, privacy, and analytical functions. Integration with blockchain, as proposed by Lodha et al [62] for blockchain-based systems using IoMT networks for e-Health care monitoring and Akbulut et al [55] for personal health record access management systems based on IOTA (IOTA Foundation) distributed ledger technology, offers new approaches to address privacy and security concerns that have long been major acceptance barriers. However, these technologies also add complexity to health care IoT ecosystems that may confuse nontechnical users, potentially creating new literacy barriers.

Trends toward edge computing identified by Albahri et al [68] discussing fault-tolerant mHealth frameworks using cloud and edge computing combinations, and Ziwei et al [83] identifying shifts from pure cloud-based models to edge and fog computing architectures to reduce latency and improve privacy reflect changing balances between needs for advanced data analytics and needs for privacy, security, and real-time processing. This balance is crucial for patient acceptance, as it potentially addresses privacy concerns while maintaining advanced analytical capabilities. This technology convergence shows the emergence of “augmented health care” paradigms where digital technologies not only assist but actively expand the capacities of both patients and health care providers. In this context, questions about how users understand and interact with these complex systems become very important for patient acceptance.

This SLR reveals an interesting paradox in health care IoT adoption: while this technology is often promoted to improve human aspects of health care services (through more time for meaningful interactions and more personal care), concerns about depersonalization remain major barriers. Kauw et al [51] identify concerns about care depersonalization as potential barriers to adoption, with some patients emphasizing the importance of human interaction in health care service experiences. This paradox raises fundamental questions about how technology is positioned in health care narratives. When technology is presented primarily as a replacement for human interaction (eg, for efficiency), resistance emerges. However, when presented as an enabler of more meaningful human interactions, acceptance tends to increase. Altinay et al [57] underscore concerns that IoT technology can reduce human aspects of health care services if not implemented wisely, highlighting the importance of implementation narratives and change management strategies in determining patient responses.

Deep analysis of literature reveals fundamental tensions between the potential benefits of health care IoT technology and concerns about privacy, autonomy, and equity, having significant implications for individual adoption decisions and societal implementation policies. Privacy issues identified in 39% of research encompass not only technical aspects of data protection but also fundamental questions about health data ownership and control, rights to be forgotten in continuous monitoring eras, and surveillance potential that can damage therapeutic relationships and patient autonomy [29,39]. Research shows that traditional informed consent models may be inadequate for addressing health care IoT system complexity where data collection is continuous, purposes may evolve, and data-sharing consequences may not be fully predictable at initial consent time [27,55].

Equity considerations emerge as very important issues given health care IoT technology’s potential to reduce or worsen existing health disparities depending on how technology is designed, implemented, and accessed. The digital divide identified in various research creates risks that those most benefiting from health care IoT technology—including older adult patients, those with chronic conditions, and individuals from lower socioeconomic backgrounds—may have the lowest likelihood of accessing or successfully using this technology [37,38]. Cost barriers, literacy requirements, infrastructure dependence, and design assumptions favoring younger, more educated, and more affluent users can all contribute to widening rather than narrowing health disparities if not explicitly addressed in technology development and implementation strategies [34,64].

SLR results reveal concerning gaps in health care IoT accessibility and adoption based on socioeconomic status, geographic location, and digital literacy. Studies by Dutta et al and Wu et al [34,53] identify that costs and accessibility are significant barriers to adoption in developing economies and among lower socioeconomic groups. These structural factors often interact with individual determinants, creating inequality cycles where those most needing health care IoT technology benefits are also least likely to adopt them. Low digital literacy, for example, not only hinders initial adoption but also affects ease of use perceptions and ultimately usage sustainability [53,58].

Socioeconomic dimensions of digital inequality identified by Ben Arfi et al [43] about how financial costs negatively affect usage intention, with stronger effects among lower socioeconomic groups, and Wu et al [53] are about higher perceived risks among lower socioeconomic status groups, partly due to concerns about costs and accessibility, which show that digital inequality is not just a function of technology characteristics but also reflects and potentially amplifies broader socioeconomic inequalities.

### Research Limitations and Challenges

This SLR has several methodological limitations that should be considered when interpreting findings and applying results to specific contexts. Patient perspective exclusivity is both a strength and a limitation. By focusing on patient acceptance, we provide depth in understanding end-user perspectives but necessarily exclude health care provider, administrator, and policy perspectives that are also crucial for successful implementation. Comprehensive understanding of health care IoT adoption requires synthesis across all stakeholder perspectives, which our review does not attempt.

Language restrictions represent a primary limitation, as our search was limited to English and Indonesian language publications. This exclusion potentially missed relevant studies published in other languages, particularly Chinese, Spanish, Arabic, German, and Portuguese, which are prominent in health care IoT research from respective regions. This language bias may particularly affect the representation of findings from Latin America, the Middle East, and East Asia beyond English-language publications, potentially limiting cross-cultural generalizability of conclusions. Geographic representation bias is evident, with 71% (44/62) of studies from Asia (n=23, 37%) and Europe (n=21, 34%), 8% (5/62) of studies from North America, 13% (8/62) of studies from the Middle East, and minimal representation from Africa, Latin America, and Oceania (n=5, 8%). This distribution may reflect both actual research activity concentration and our English-language search limitation. Findings regarding acceptance factors and barriers may not generalize to underrepresented regions with different health care systems, cultural norms, technological infrastructure, and patient populations.

Database coverage limitations exist despite searching eight major databases. Some relevant studies may have been missed due to database indexing variations, search term limitations, or publication in journals not indexed in the searched databases. Our search strategy, while comprehensive, relied on specific keyword combinations that may not have captured all relevant papers using alternative terminology for IoT technologies or acceptance constructs. Gray literature exclusion means we did not systematically search conference proceedings, dissertations, technical reports, or unpublished studies beyond what was captured in Google Scholar. This decision, made to focus on peer-reviewed evidence, may introduce publication bias as studies with positive or significant findings are more likely to be published in peer-reviewed journals than those with null or negative results. The true extent of failed implementations or low acceptance may be underrepresented in our synthesis.

There was limited subgroup analysis due to available data and reporting heterogeneity. While we identified important moderating factors (digital literacy, age, and health conditions), many included studies did not report sufficient data for systematic subgroup analyses. Our moderator findings therefore rely on studies that explicitly examined these interactions rather than systematic subgroup meta-analyses across all studies. Cross-sectional design dominance among included studies limits our ability to assess causality or track acceptance patterns over time. Most studies examined acceptance at single time points, meaning our understanding of how factors influence initial adoption may differ from factors affecting continued use, but evidence on this distinction is limited. Longitudinal research is critically needed to understand the temporal dynamics of IoT health care acceptance.

Future research needs to address these limitations through more diverse geographic representation, standardized measurement approaches, longitudinal study designs, inclusive recruitment strategies, and systematic attention to negative cases and implementation failures as important learning sources about barriers and facilitators for sustainable health care IoT adoption. Despite these limitations, we believe our systematic approach, comprehensive database searching, rigorous quality assessment, and transparent synthesis methods provide a reliable synthesis of current evidence on patient acceptance of IoT in health care. The consistency of main findings across studies with different designs, populations, and contexts strengthens confidence in core conclusions regarding the importance of perceived usefulness, trust, digital literacy, and the need for UCD approaches.

### Practical Implications of Our Approach

This SLR provides significant contributions to diverse stakeholders in health care service ecosystems with comprehensive benefits. From theoretical perspectives, this SLR contributes to the body of knowledge about technology acceptance in health care service contexts by providing a comprehensive synthesis of factors affecting patient adoption, building and expanding existing theories.

For technology developers: implementation teams should prioritize UCD and onboarding that explicitly reduces perceived effort and cognitive burden, particularly for older adults and low-literacy users, because ease-of-use and support features repeatedly emerge as acceptance-enabling conditions in the included literature.

For health care organizations: implementation strategies must be informed by a comprehensive synthesis of facilitators, barriers, and proven enhancement approaches, enabling evidence-based deployment planning.

For policymakers: equity considerations and regulatory frameworks or guidelines need to be identified through systematic analysis of barriers and contextual variations across different populations and settings that facilitate adoption while ensuring patient safety. Policymakers should strengthen governance frameworks that increase patient trust—particularly regarding privacy, security, and accountability—because security and privacy concerns are among the most frequently reported acceptance barriers in this review.

For researchers: identification of methodological gaps, under-researched populations and contexts, and priority areas for future investigation to advance both theory and practice. Future studies should move beyond cross-sectional designs by using longitudinal and implementation-focused methods and by reporting standardized acceptance constructs and context variables to enable stronger synthesis and eventual meta-analytic work.

### Future Research Directions

Based on gaps identified in current literature and emerging trends in health care IoT development, several important research priorities emerge that can advance theoretical understanding and practical implementation of patient-centered health care IoT solutions. Longitudinal research represents a primary priority given the current lack of understanding about how adoption patterns, user experiences, and health outcomes evolve over extended periods with health care IoT technology. Multiyear cohort studies tracking users from initial adoption to long-term use can provide important insights into factors driving continued engagement, technology abandonment patterns, evolution in user needs and preferences, and actual health usage and health care impacts of long-term IoT use [30,35]. Comparative research across various cultural, economic, and health care system contexts emerges as a second major priority, given the current geographic bias in the literature and the need to understand how contextual factors affect adoption patterns and optimal implementation strategies. Cross-cultural studies can illuminate how factors such as collectivism vs individualism, power distance, uncertainty avoidance, and other cultural dimensions affect attitudes toward health monitoring, data sharing, technology adoption, and relationships with health care providers in health care IoT system contexts [22,48]. Economic comparative research is also needed to understand how different health care financing systems, insurance models, and economic development levels affect access and sustainability of health care IoT interventions.

Implementation science research represents the third critical priority, focusing on understanding how evidence-based health care IoT interventions can be successfully translated into real-world practice settings with diverse stakeholders, competing priorities, resource constraints, and complex organizational dynamics. Implementation research can answer questions about optimal training models for health care providers, effective strategies for patient orientation and support, sustainable financing models for long-term technology maintenance and updates, integration approaches minimizing workflow disruption, and scaling strategies maintaining fidelity while adapting to local contexts [60,69]. Methodological innovation represents the fourth priority area, with a need to develop new research approaches that can better capture the complexity of health care IoT adoption and use. This includes the development of standardized outcome measures for evaluating health care IoT interventions, the integration of objective usage data with subjective reports for comprehensive understanding of user experiences, participatory research methods involving patients as research partners in designing and evaluating health care IoT solutions, and mixed reality research approaches that can study health care IoT use in natural settings while maintaining experimental control [44,50]. Emerging technology research represents the fifth priority, given rapid innovation in fields such as AI integration, voice interfaces, augmented reality, blockchain applications, and edge computing that can significantly change health care IoT capability landscapes and user experiences. Research in these emerging areas needs to proactively address user acceptance factors, ethical implications, implementation challenges, and potential unintended consequences before technologies are widely deployed, rather than studying acceptance after technologies are already established in markets [27,62].

### Conclusions

This SLR of 62 studies published between 2016 and 2024 provides comprehensive evidence that patient acceptance of IoT technology in health care represents a complex, multidimensional phenomenon requiring holistic approaches that extend beyond technical sophistication to encompass human, organizational, and systemic considerations. With appropriate attention to patient perspectives, UCD, digital equity, and multilevel implementation strategies, IoT technology holds substantial potential to transform health care delivery toward more proactive, personalized, accessible, and effective services. However, realizing this potential requires sustained commitment to placing patients at the center of technology design, implementation, and evaluation processes, ensuring technological advancement serves human flourishing rather than merely demonstrating technical capabilities. The evidence synthesized in this review provides a foundation for such patient-centered approaches, but continued research, evaluation, and adaptation will be essential as both technologies and health care contexts continue to evolve.

## Supplementary material

10.2196/81260 Multimedia Appendix 1 Database search strategy for internet of things literature review.

10.2196/81260 Multimedia Appendix 2 Internet of things systematic literature review data extraction table (version 1.2).

10.2196/81260 Multimedia Appendix 3 Mixed Methods Appraisal Tool quality assessment for internet of things systematic literature review.

10.2196/81260 Multimedia Appendix 4 Summary of methodological quality appraisal (Mixed Methods Appraisal Tool Assessment).

10.2196/81260 Multimedia Appendix 5 Strategies for enhancing patient acceptance of internet of things in health care(version 1.2).

10.2196/81260 Multimedia Appendix 6 Table study characteristics (version 1.2).

10.2196/81260 Checklist 1 PRISMA 2020 27-Item Checklist V1.2.

## Abbreviations

IoMT: internet of medical things
IoT: internet of things
mHealth: mobile health
MMAT: Mixed Methods Appraisal Tool
PRISMA: Preferred Reporting Items for Systematic Reviews and Meta-Analyses
RQ: research question
SLR: systematic literature review
TAM: technology acceptance model
UCD: user-centered design
UTAUT: unified theory of acceptance and use of technology
