# Supplementary material for: Patient Acceptance and Barriers to IoT Usage in Health Care: Systematic Literature Review
Source: JMIR Mhealth Uhealth. 2026 Jul 31;14:e81260. doi: 10.2196/81260 (PMC13430414; doi:10.2196/81260)
Supplement: Multimedia Appendix 1 — Database search strategy for internet of things literature review. [file mhealth-v14-e81260-s001.pdf]

FULLY REPRODUCIBLE SEARCH STRATEGY

For Systematic Literature Review: Patient Acceptance of IoT in Healthcare

SEARCH PERIOD:
- Article search range: January 1, 2016 - December 31, 2024
- Date of search execution: [17 – 20 March 2025]

LANGUAGE LIMITS:
- English OR Indonesian

1. PUBMED/MEDLINE

Database URL: https://pubmed.ncbi.nlm.nih.gov/
Search date: [18 March 2025]
Platform: NLM PubMed

Search Strategy:

1 "Internet of Things"[Title/Abstract] OR "IoT"[Title/Abstract] OR "connected health"[Title/Abstract] OR "smart health"[Title/Abstract] OR "telemedicine"[Title/Abstract] OR "telehealth"[Title/Abstract] OR "mHealth"[Title/Abstract] OR "eHealth"[Title/Abstract] OR "wearable device*"[Title/Abstract]

2 "patient*"[Title/Abstract] OR "consumer*"[Title/Abstract] OR "customer*"[Title/Abstract] OR "user*"[Title/Abstract]

3 "acceptance"[Title/Abstract] OR "adoption"[Title/Abstract] OR "readiness"[Title/Abstract] OR "willingness"[Title/Abstract] OR "perception*"[Title/Abstract] OR "attitude*"[Title/Abstract] OR "experience*"[Title/Abstract] OR "engagement"[Title/Abstract] OR "trust"[Title/Abstract] OR "retention*"[Title/Abstract]

4 "health"[Title/Abstract] OR "healthcare"[Title/Abstract] OR "health services"[Title/Abstract] OR "medical"[Title/Abstract] OR "hospital*"[Title/Abstract] OR "clinic*"[Title/Abstract] OR "home care"[Title/Abstract]

5 1 AND 2 AND 3 AND 4

Filters applied:
- Publication date: 2016/01/01 - 2024/12/31
- Language: English OR Indonesian
- Article types: Journal Article, Review, Systematic Review

Results: [374] records

2. SCOPUS

Database URL: https://www.scopus.com/
Search date: [17 March 2025]

Search Strategy:

TITLE-ABS-KEY (("Internet of Things" OR "IoT" OR "connected health" OR "smart health" OR "telemedicine" OR "telehealth" OR "mHealth" OR "eHealth" OR "wearable device*") AND ("patient*" OR "consumer*" OR "customer*" OR "user*") AND ("acceptance" OR "adoption" OR "readiness" OR "willingness" OR "perception*" OR "attitude*" OR "experience*" OR "engagement" OR "trust" OR "retention*") AND ("health" OR "healthcare" OR "health services" OR "medical" OR "hospital*" OR "clinic*" OR "home care"))
AND PUBYEAR > 2015 AND PUBYEAR < 2025
AND (LIMIT-TO (LANGUAGE , "English") OR LIMIT-TO (LANGUAGE , "Indonesian"))
AND (LIMIT-TO (DOCTYPE , "ar") OR LIMIT-TO (DOCTYPE , "re"))

Results: [847] records

3. IEEE XPLORE
Database URL: https://ieeexplore.ieee.org/
Search date: [17 March 2025]

Search Strategy:

("Abstract":"Internet of Things" OR "Abstract":"IoT" OR "Abstract":"connected health" OR "Abstract":"smart health" OR "Abstract":"telemedicine" OR "Abstract":"telehealth" OR "Abstract":"mHealth" OR "Abstract":"eHealth" OR "Abstract":"wearable device*")
AND
("Abstract":"patient*" OR "Abstract":"consumer*" OR "Abstract":"user*")
AND
("Abstract":"acceptance" OR "Abstract":"adoption" OR "Abstract":"perception*" OR "Abstract":"attitude*" OR "Abstract":"experience*")
AND
("Abstract":"health" OR "Abstract":"healthcare" OR "Abstract":"medical" OR "Abstract":"hospital*")

Filters:
- Year: 2016-2024
- Content Type: Journals

Results: [528] records

4. WEB OF SCIENCE
Database URL: https://www.webofscience.com/
Search date: [19 March 2025]

Search Strategy:

TS=("Internet of Things" OR "IoT" OR "connected health" OR "smart health" OR "telemedicine" OR "telehealth" OR "mHealth" OR "eHealth" OR "wearable device*")
AND

TS=("patient*" OR "consumer*" OR "user*")
AND
TS=("acceptance" OR "adoption" OR "readiness" OR "willingness" OR "perception*" OR "attitude*" OR "experience*" OR "engagement" OR "trust")
AND
TS=("health" OR "healthcare" OR "health services" OR "medical" OR "hospital*" OR "clinic*")

Refined by:
- Publication Years: 2016-2024
- Languages: English OR Indonesian
- Document Types: Article OR Review Article

Results: [49] records

5. SCIENCEDIRECT
Database URL: https://www.sciencedirect.com/
Search date: [18 March 2025]

Search Strategy:

TITLE-ABSTR-KEY("Internet of Things" OR "IoT" OR "connected health" OR "smart health" OR "telemedicine" OR "telehealth" OR "mHealth" OR "eHealth" OR "wearable device*")
AND TITLE-ABSTR-KEY("patient*" OR "consumer*" OR "user*")
AND TITLE-ABSTR-KEY("acceptance" OR "adoption" OR "perception*" OR "attitude*" OR "experience*")
AND TITLE-ABSTR-KEY("health" OR "healthcare" OR "medical" OR "hospital*")

Filters:
- Years: 2016-2024
- Article type: Research articles, Review articles
- Journals

Results: [418] records

6. ACM DIGITAL LIBRARY
Database URL: https://dl.acm.org/
Search date: [19 March 2025]

Search Strategy:

[[Abstract: "internet of things"] OR [Abstract: iot] OR [Abstract: "connected health"] OR [Abstract: "smart health"] OR [Abstract: telemedicine] OR [Abstract: mhealth] OR [Abstract: ehealth] OR [Abstract: "wearable device"]]
AND [[Abstract: patient] OR [Abstract: consumer] OR [Abstract: user]]
AND [[Abstract: acceptance] OR [Abstract: adoption] OR [Abstract: perception] OR [Abstract: attitude] OR [Abstract: experience]]
AND [[Abstract: health] OR [Abstract: healthcare] OR [Abstract: medical] OR [Abstract: hospital]]

Filters:
- Published since: 2016
- Published until: 2024

Results: [32] records

7. PROQUEST
Database URL: https://www.proquest.com/
Search date: [20 March 2025]

Search Strategy:

ab("Internet of Things" OR "IoT" OR "connected health" OR "smart health" OR "telemedicine" OR "telehealth" OR "mHealth" OR "eHealth" OR "wearable device*")
AND ab("patient*" OR "consumer*" OR "user*")
AND ab("acceptance" OR "adoption" OR "perception*" OR "attitude*" OR "experience*")
AND ab("health" OR "healthcare" OR "medical" OR "hospital*")

Filters:
- Date: 2016-2024
- Language: English, Indonesian
- Document type: Peer-reviewed journals

Results: [289] records

8. GOOGLE SCHOLAR
Database URL: https://scholar.google.com/
Search date: [20 March 2025]

Search Strategy:

allintitle: ("Internet of Things" OR IoT OR "connected health") AND (patient OR user) AND (acceptance OR adoption) AND (healthcare OR medical)

Settings:
- Year range: 2016-2024
- First 200 results reviewed for relevance

Results: [14] records

Note: Google Scholar used for grey literature and supplementary searching only

DEDUPLICATION PROCESS:
1. Software used: Mendeley Desktop & Zotero
2. Automatic duplicate detection based on: DOI, Title, Author, Year
3. Manual verification of potential duplicates
4. Total duplicates removed: 805 records

SUPPLEMENTARY SEARCHES:
1. Reference list screening: All included studies' reference lists manually screened
2. Citation tracking: Forward citation searching using Google Scholar
3. Expert consultation: [If applicable, specify experts consulted]

NOTES:
- All searches were conducted by [Zaenal Arifin]
- Search strategy reviewed by [Prof. Dr. Putu Wuri Handayani]
- Data repository: [OSF]
