# Supplementary material for: Patient Acceptance and Barriers to IoT Usage in Health Care: Systematic Literature Review
Source: JMIR Mhealth Uhealth. 2026 Jul 31;14:e81260. doi: 10.2196/81260 (PMC13430414; doi:10.2196/81260)
Supplement: Multimedia Appendix 2 — Internet of things systematic literature review data extraction table (version 1.2). [file mhealth-v14-e81260-s002.docx]

**DATA EXTRACTION TABLES - SYSTEMATIC LITERATURE REVIEW**

**Title: Patient Acceptance and Barriers to Utilizing IoT in Healthcare Services**

**Total Studies: 62 Journals (2016-2024)**
**Databases: PubMed, Scopus, IEEE Xplore, Web of Science, ScienceDirect, ACM Digital Library, ProQuest, Google Scholar**

**TABLE 1: BIBLIOGRAPHIC & METHODOLOGICAL DATA**

| No | Author & Year | Full Title | Journal | Quartile | Country | Research Design | Sample | Analysis Method |
| --- | --- | --- | --- | --- | --- | --- | --- | --- |
| 1 | Kos & Umek (2019) [47] | Wearable Sensor Devices for Prevention and Rehabilitation in Healthcare: Swimming Exercise With Real-Time Therapist Feedback | IEEE Internet of Things Journal | Q1 | Slovenia | Experimental | N/A | Technical validation |
| 2 | Akbulut et al. (2023) [55] | Designing a private and secure personal health records access management system: A solution based on IOTA distributed ledger technology | Sensors (MDPI) | Q1 | Turkey | Framework Development | N/A | System design & security analysis |
| 3 | Albahri et al. (2019) [68] | Fault-tolerant mHealth framework in the context of IoT-based real-time wearable health data sensors | IEEE Access | Q1 | Iraq/ Malaysia | Framework Development | N/A | Architecture design |
| 4 | Alhasan et al. (2022) [28] | A case-study to examine doctors' intentions to use IoT healthcare devices in Iraq during COVID-19 pandemic | International Journal of Pervasive Computing and Communications | Q3 | Iraq | Quantitative - Survey | Doctors | TAM/ UTAUT |
| 5 | AlQudah et al. (2021) [45] | Technology Acceptance in Healthcare: A Systematic Review | Applied Sciences | Q2 | Multi-country | Systematic Review | 50+ studies | Narrative synthesis |
| 6 | Al-Rawashdeh et al. (2022) [18] | IoT Adoption and Application for Smart Healthcare: A Systematic Review | Sensors | Q1 | Malaysia | Systematic Review | 60+ studies | Thematic analysis |
| 7 | Al-rawashdeh et al. (2024) [46] | Effective factors for the adoption of IoT applications in nursing care: A theoretical framework for smart healthcare | Journal of Building Engineering | Q1 | Malaysia | Framework Development | N/A | Theoretical modeling |
| 8 | Alruwaili et al. (2023) [38] | Digital Health Interventions for Promoting Healthy Aging: A Systematic Review of Adoption Patterns, Efficacy, and User Experience | Sustainability | Q1 | Saudi Arabia | Systematic Review | Elderly patients | Meta-synthesis |
| 9 | Altinay et al. (2023) [57] | The effect of hospitableness on positive emotions, experience, and well-being of hospital patients | International Journal of Hospitality Management | Q1 | Turkey/UK | Quantitative - Survey | Hospital patients | SEM |
| 10 | Arfi et al. (2021) [73] | The role of trust in intention to use the IoT in eHealth: Application of the modified UTAUT in a consumer context | Technological Forecasting and Social Change | Q1 | France | Quantitative - Survey | 348 consumers | SEM/PLS |
| 11 | Ben Arfi et al. (2021) [43] | Understanding acceptance of eHealthcare by IoT natives and IoT immigrants: An integrated model of UTAUT, perceived risk, and financial cost | Technological Forecasting and Social Change | Q1 | France | Quantitative - Survey | 418 respondents | SEM/PLS |
| 12 | Binci et al. (2022) [71] | Toward digital transformation in healthcare: A framework for remote monitoring adoption | TQM Journal | Q1 | Italy | Framework Development | Healthcare organizations | Case study analysis |
| 13 | Bodur et al. (2019) [58] | Perceptions of Turkish health professional students toward the effects of the internet of things (IOT) technology in the future | Nurse Education Today | Q1 | Turkey | Quantitative - Survey | 472 health students | Descriptive statistics |
| 14 | Bures et al. (2022) [49] | A Sensor Network Utilizing Consumer Wearables for Telerehabilitation of Post-Acute COVID-19 Patients | IEEE Internet of Things Journal | Q1 | Czech Republic | Experimental/ Observational | COVID-19 patients | Clinical validation |
| 15 | Cahill et al. (2019) [72] | IoT/sensor-based infrastructures promoting a sense of home, independent living, comfort and wellness | Sensors | Q1 | Ireland/ Germany | Qualitative - Interviews | Elderly users | Thematic analysis |
| 16 | Calvillo-Arbizu et al. (2021) [31] | Internet of Things in health: Requirements, issues, and gaps | Computer Methods and Programs in Biomedicine | Q1 | Spain | Literature Review | N/A | Gap analysis |
| 17 | Cleveland & Haddara (2023) [63] | Internet of Things for diabetics: Identifying adoption issues | Internet of Things | Q1 | Norway | Qualitative - Interviews | Diabetic patients | Thematic analysis |
| 18 | Du et al. (2021) [54] | A qualitative study of patients' experiences and acceptance of computerised cognitive behavioural therapy in primary care, Scotland | Cognitive Behaviour Therapist | Q2 | Scotland/ UK | Qualitative - Interviews | 15 patients | Thematic analysis |
| 19 | Dutta et al. (2023) [34] | Adoption of IoT-based healthcare devices: An empirical study of end consumers in an emerging economy | Paladyn: Journal of Behavioral Robotics | Q3 | India | Quantitative - Survey | 312 consumers | SEM |
| 20 | Fehringer & Stary (2023) [56] | User-Informed Adaptation in IoT Home Healthcare: Grounding Development in Empirical Evidence | Journal of Theoretical and Applied Electronic Commerce Research | Q1 | Austria | Mixed Methods | 45 elderly users | Participatory design |
| 21 | Fischer et al. (2020) [60] | ElHealth: Using Internet of Things and data prediction for elastic management of human resources in smart hospitals | Engineering Applications of Artificial Intelligence | Q1 | Germany | Framework Development | Hospital staff | System modeling |
| 22 | Fraterman et al. (2023) [30] | An eHealth App (CAPABLE) Providing Symptom Monitoring, Well-Being Interventions, and Educational Material for Patients With Melanoma Treated With Immune Checkpoint Inhibitors: Protocol for an Exploratory Intervention Trial | JMIR Research Protocols | Q3 | Netherlands | Protocol/ Mixed Methods | Melanoma patients | Intervention design |
| 23 | Gardas (2022) [59] | Organizational hindrances to Healthcare 4.0 adoption: An multi-criteria decision analysis framework | Journal of Multi-Criteria Decision Analysis | Q2 | India | Quantitative - MCDA | Healthcare organizations | AHP/ TOPSIS |
| 24 | Gellert et al. (2023) [41] | How virtual triage can improve patient experience and satisfaction: A narrative review and look forward | Telemedicine Reports | Q2 | Multi-country | Qualitative - Narrative | N/A | narrative analysis |
| 25 | Ghiwaa et al. (2023) [19] | Telemedicine Adoption for Healthcare Delivery: A Systematic Review | International Journal of Advanced Computer Science and Applications | Q3 | UK | Systematic Review | 45+ studies | PRISMA methodology |
| 26 | Grant et al. (2019) [44] | Home health monitoring around the time of surgery: Qualitative study of patients' experiences before and after joint replacement | BMJ Open | Q1 | UK | Qualitative - Interviews | 24 patients | Framework analysis |
| 27 | Hayat et al. (2022) [48] | Exploring the adoption of wearable healthcare devices among the Pakistani adults with dual analysis techniques | Technology in Society | Q1 | Pakistan/ Malaysia | Quantitative - Survey | 419 adults | SEM/ ANN |
| 28 | He et al. (2024) [23] | A survey of Internet of Medical Things: Technology, application and future directions | Digital Communications and Networks | Q1 | China | Survey/Review | N/A | Technical analysis |
| 29 | Hjelm & Hedlund (2022) [74] | Internet-of-Things (IoT) in healthcare and social services -- experiences of a sensor system for notifications of deviant behaviours in the home from the users' perspective | Health Informatics Journal | Q3 | Sweden | Qualitative - Interviews | 18 elderly users | Content analysis |
| 30 | Hossain et al. (2021) [35] | Factors influencing adoption model of continuous glucose monitoring devices for internet of things healthcare | Internet of Things | Q1 | Malaysia | Quantitative - Survey | 384 diabetic patients | SEM/PLS |
| 31 | Hui et al. (2021) [36] | Patients' and clinicians' perceived trust in internet-of-things systems to support asthma self-management: Qualitative interview study | JMIR mHealth and uHealth | Q1 | UK | Qualitative - Interviews | 25 participants (patients & clinicians) | Thematic analysis |
| 32 | Karahoca et al. (2018) [52] | Examining intention to adopt to internet of things in healthcare technology products | Kybernetes | Q1 | Turkey | Quantitative - Survey | 218 respondents | SEM |
| 33 | Kauw et al. (2020) [51] | Mobile health in cardiac patients: An overview on experiences and challenges of stakeholders involved in daily use and development | BMJ Innovations | Q3 | Netherlands | Qualitative - Interviews | 39 stakeholders | Thematic analysis |
| 34 | Kotronis et al. (2019) [50] | Evaluating Internet of Medical Things (IoMT)-Based Systems from a Human-Centric Perspective | Internet of Things | Q1 | Greece | Framework Development | N/A | Evaluation framework |
| 35 | Kronlid et al. (2024) [24] | Sociotechnical analysis of factors influencing IoT adoption in healthcare: A systematic review | Technology in Society | Q1 | Sweden | Systematic Review | 75+ studies | Socio-technical analysis |
| 36 | Kwon et al. (2022) [66] | Review of smart hospital services in real healthcare environments | Healthcare Informatics Research | Q2 | South Korea | Literature Review | N/A | Service analysis |
| 37 | Lee et al. (2018) [75] | A cardiopulmonary monitoring system for patient transport within hospitals using mobile internet of things technology: Observational validation study | JMIR mHealth and uHealth | Q1 | South Korea | Qualitative -Observational | 30 patients | Clinical validation |
| 38 | Lodha et al. (2023) [62] | A blockchain-based secured system using the Internet of Medical Things (IOMT) network for e-Healthcare monitoring | Measurement: Sensors | Q2 | India | Framework Development | N/A | Blockchain architecture |
| 39 | Lu et al. (2018) [40] | Internet of Things: A systematic review of the business literature from the user and organisational perspectives | Technological Forecasting and Social Change | Q1 | UK | Systematic Review | 134 studies | User/ organizational perspective |
| 40 | Martínez-Caro et al. (2018) [76] | Healthcare service evolution towards the Internet of Things: An end-user perspective | Technological Forecasting and Social Change | Q1 | Spain | Quantitative - Survey | 203 end-users | SEM |
| 41 | Messinis et al. (2024) [27] | Enhancing Internet of Medical Things security with artificial intelligence: A comprehensive review | Computers in Biology and Medicine | Q1 | Greece | Qualitative – Analysis Review | N/A | AI security analysis |
| 42 | Mittelstadt (2017) [39] | Ethics of the health-related internet of things: A narrative review | Ethics and Information Technology | Q1 | UK | Qualitative -Narrative | N/A | Ethical analysis |
| 43 | Mittelstadt (2017) [77] | Designing the health-related internet of things: Ethical principles and guidelines | Information | Q2 | UK | Conceptual Framework | N/A | Ethical framework |
| 44 | Pulimamidi (2024) [78] | To enhance customer (or patient) experience based on IoT analytical study through technology (IT) transformation for E-healthcare | Measurement: Sensors | Q2 | India | Mixed-Method | N/A | Experience analysis |
| 45 | Ramdani et al. (2020) [79] | Exploring the determinants of mobile health adoption by hospitals in China: Empirical study | JMIR Medical Informatics | Q1 | China/ UK | Quantitative - Survey | 173 hospitals | Regression analysis |
| 46 | Rejeb et al. (2023) [32] | The Internet of Things (IoT) in healthcare: Taking stock and moving forward | Internet of Things | Q1 | Multi-country | Literature Review | N/A | Bibliometric analysis |
| 47 | Ryu et al. (2016) [80] | Inpatient satisfaction and usage patterns of personalized smart bedside station system for patient-centered service at a tertiary university hospital | International Journal of Medical Informatics | Q2 | South Korea | Quantitative - Survey | 200 inpatients | Descriptive/ correlation |
| 48 | Sahin et al. (2021) [70] | Perceptions and acceptance of telemedicine among medical oncologists before and during the COVID-19 pandemic in Turkey | Supportive Care in Cancer | Q1 | Turkey | Quantitative - Survey | 147 oncologists | Comparative analysis |
| 49 | Sharma & Joshi (2021) [61] | Barriers to blockchain adoption in health-care industry: An Indian perspective | Journal of Global Operations and Strategic Sourcing | Q1 | India | Qualitative - Expert interviews | 15 experts | ISM/ MICMAC |
| 50 | Streeper et al. (2019) [65] | Improving Fluid Intake Behavior Among Patients With Kidney Stones: Understanding Patients' Experiences and Acceptability of Digital Health Technology | Urology | Q1 | USA | Qualitative - Interviews | 20 patients | Thematic analysis |
| 51 | Tortorella et al. (2020) [64] | Effects of contingencies on Healthcare 4.0 technologies adoption and barriers in emerging economies | Technological Forecasting and Social Change | Q1 | Brazil/ Italy | Quantitative - Survey | 106 hospitals | Regression/ moderation |
| 52 | Tu et al. (2022) [26] | Exploring Usability and Patient Attitude towards a Smart Hospital Service with the Technology Acceptance Model | International Journal of Environmental Research and Public Health | Q2 | Taiwan | Quantitative - Survey | 395 patients | SEM/TAM |
| 53 | Wakili & Bakkali (2024) [29] | Internet of Things in healthcare: An adaptive ethical framework for IoT in digital health | Clinical eHealth | Q2 | Morocco | Framework Development | N/A | Ethical framework |
| 54 | Wen et al. (2022) [67] | Implementation and experience of an innovative smart patient care system: A cross-sectional study | BMC Health Services Research | Q1 | Taiwan | Mixed methods | 302 patients & staff | Cross-sectional |
| 55 | Westphal et al. (2020) [69] | A patient-centered information system (myED) for emergency care journeys: Design, development, and initial adoption | JMIR Formative Research | Q2 | Israel | Mixed Methods | 156 patients | UCD approach |
| 56 | Wu et al. (2023) [53] | User Interface Characteristics Influencing Medical Self-Service Terminals Behavioral Intention and Acceptance by Chinese Elderly: An Empirical Examination Based on an Extended UTAUT Model | Sustainability | Q1 | China | Quantitative - Survey | 428 elderly | SEM/ UTAUT |
| 57 | Wu & Ho (2023) [37] | Barriers to Telemedicine Adoption during the COVID-19 Pandemic in Taiwan: Comparison of Perceived Risks by Socioeconomic Status Correlates | International Journal of Environmental Research and Public Health | Q2 | Taiwan | Quantitative - Survey | 1,042 respondents | Comparative analysis |
| 58 | Yang et al. (2022) [25] | Modeling the Intention and Adoption of Wearable Fitness Devices: A Study Using SEM-PLS Analysis | Frontiers in Public Health | Q1 | China/ Malaysia | Quantitative - Survey | 406 respondents | SEM-PLS |
| 59 | Zeadally & Bello (2021) [42] | Harnessing the power of Internet of Things based connectivity to improve healthcare | Internet of Things | Q1 | USA/ UK | Qualitative -Conceptual Review | N/A | Connectivity analysis |
| 60 | Zhang et al. (2024) [22] | In-depth examination of the functionality and performance of the Internet hospital information platform: Development and usability study | Journal of Medical Internet Research | Q1 | China | Mixed Methods | 500+ users | Usability testing |
| 61 | Ziwei et al. (2024) [81] | The applications of internet of things in smart healthcare sectors: A bibliometric and deep study | Heliyon | Q1 | China | Qualitative -Bibliometric Study | 2,847 publications | VOSviewer/ CiteSpace |
| 62 | Zobair et al. (2023) [82] | Systematic review of Internet of medical things for cardiovascular disease prevention among Australian first nations | Heliyon | Q1 | Australia | Systematic Review | 23 studies | PRISMA methodology |

**TABLE 2: IOT TECHNOLOGY TYPES STUDIED**

| No | Author & Year | IoT Technology Type | Specific Application | Complexity | Healthcare Domain |
| --- | --- | --- | --- | --- | --- |
| 1 | Kos & Umek (2019) [47] | Wearable sensors | Real-time swimming exercise monitoring | Medium | Rehabilitation |
| 2 | Akbulut et al. (2023) [55] | Personal health records system | IOTA distributed ledger for data security | High | General health data management |
| 3 | Albahri et al. (2019) [68] | mHealth framework | Fault-tolerant wearable sensors | High | Real-time monitoring |
| 4 | Alhasan et al. (2022) [28] | IoT healthcare devices | General medical IoT devices | Medium | COVID-19 healthcare |
| 5 | AlQudah et al. (2021) [45] | Multiple technologies | Various health technologies | Varied | General healthcare |
| 6 | Al-Rawashdeh et al. (2022) [18] | Smart healthcare IoT | Multiple IoT applications | Varied | Smart healthcare |
| 7 | Al-rawashdeh et al. (2024) [46] | IoT in nursing care | Nursing-specific applications | Medium-High | Nursing care |
| 8 | Alruwaili et al. (2023) [38] | Digital health interventions | Aging-focused technologies | Varied | Healthy aging |
| 9 | Altinay et al. (2023) [57] | Smart hospital systems | Hospital patient experience tech | Medium | Hospital care |
| 10 | Arfi et al. (2021) [73] | eHealth IoT systems | Consumer eHealth devices | Medium | General eHealth |
| 11 | Ben Arfi et al. (2021) [43] | eHealthcare IoT | Consumer health devices | Medium | General healthcare |
| 12 | Binci et al. (2022) [71] | Remote monitoring systems | Chronic disease monitoring | Medium-High | Remote patient monitoring |
| 13 | Bodur et al. (2019) [58] | General IoT in health | Future IoT applications | Varied | General healthcare |
| 14 | Bures et al. (2022) [49] | Consumer wearables | Post-COVID telerehabilitation | Medium | Telerehabilitation |
| 15 | Cahill et al. (2019) [72] | IoT/sensor infrastructure | Home monitoring for elderly | Medium | Independent living |
| 16 | Calvillo-Arbizu et al. (2021) [31] | General health IoT | Multiple IoT technologies | Varied | General healthcare |
| 17 | Cleveland & Haddara (2023) [63] | IoT for diabetics | Continuous glucose monitors, insulin pumps | High | Diabetes management |
| 18 | Du et al. (2021) [54] | Computerised CBT | Digital therapy platform | Medium | Mental health |
| 19 | Dutta et al. (2023) [34] | IoT healthcare devices | Consumer health devices | Medium | General healthcare |
| 20 | Fehringer & Stary (2023) [56] | IoT home healthcare | Adaptive home monitoring | High | Home healthcare |
| 21 | Fischer et al. (2020) [60] | Smart hospital IoT | Resource management systems | High | Hospital management |
| 22 | Fraterman et al. (2023) [30] | eHealth app (CAPABLE) | Symptom monitoring app | Medium | Cancer care |
| 23 | Gardas (2022) [59] | Healthcare 4.0 technologies | Multiple technologies | High | Hospital systems |
| 24 | Gellert et al. (2023) [41] | Virtual triage systems | Telemedicine triage | Medium | Emergency care |
| 25 | Ghiwaa et al. (2023) [19] | Telemedicine systems | Remote consultation platforms | Medium | Telemedicine |
| 26 | Grant et al. (2019) [44] | Home health monitoring | Post-surgery monitoring | Medium | Surgical care |
| 27 | Hayat et al. (2022) [48] | Wearable healthcare devices | Fitness trackers, smartwatches | Low-Medium | General health monitoring |
| 28 | He et al. (2024) [23] | Internet of Medical Things | Multiple IoMT technologies | Varied | Comprehensive healthcare |
| 29 | Hjelm & Hedlund (2022) [74] | IoT sensor system | Behavioral monitoring sensors | Medium | Elderly care |
| 30 | Hossain et al. (2021) [35] | Continuous glucose monitors | CGM devices for diabetes | High | Diabetes management |
| 31 | Hui et al. (2021) [36] | IoT asthma management | Asthma self-management systems | Medium-High | Chronic disease |
| 32 | Karahoca et al. (2018) [52] | Healthcare IoT products | General IoT devices | Medium | General healthcare |
| 33 | Kauw et al. (2020) [51] | Mobile health (mHealth) | Cardiac patient apps | Medium | Cardiac care |
| 34 | Kotronis et al. (2019) [50] | IoMT systems | Medical IoT devices | Medium-High | Medical care |
| 35 | Kronlid et al. (2024) [24] | Multiple IoT technologies | Varied applications | Varied | General healthcare |
| 36 | Kwon et al. (2022) [66] | Smart hospital services | Hospital IoT systems | High | Hospital care |
| 37 | Lee et al. (2018) [75] | Cardiopulmonary monitoring | Mobile IoT monitoring | High | Patient transport |
| 38 | Lodha et al. (2023) [62] | Blockchain-IoMT | Secured monitoring system | High | e-Healthcare monitoring |
| 39 | Lu et al. (2018) [40] | General IoT | Business IoT applications | Varied | General healthcare |
| 40 | Martínez-Caro et al. (2018) [76] | Healthcare service IoT | End-user health services | Medium | General healthcare |
| 41 | Messinis et al. (2024) [27] | IoMT with AI | AI-enhanced security systems | High | Medical security |
| 42 | Mittelstadt (2017) [39] | Health-related IoT | Various health IoT | Varied | Ethics focus |
| 43 | Mittelstadt (2017) [77] | Health-related IoT | Design principles | Varied | Ethics focus |
| 44 | Pulimamidi (2024) [78] | E-healthcare IoT | Patient experience tech | Medium | Patient experience |
| 45 | Ramdani et al. (2020) [79] | Mobile health (mHealth) | Hospital mHealth systems | Medium | Hospital care |
| 46 | Rejeb et al. (2023) [32] | Healthcare IoT | Multiple IoT applications | Varied | General healthcare |
| 47 | Ryu et al. (2016) [80] | Smart bedside station | Personalized patient system | Medium | Hospital inpatient |
| 48 | Sahin et al. (2021) [70] | Telemedicine systems | Remote oncology consultation | Medium | Cancer care |
| 49 | Sharma & Joshi (2021) [61] | Blockchain in healthcare | Blockchain IoT systems | High | Healthcare systems |
| 50 | Streeper et al. (2019) [65] | Digital health tech | Fluid intake monitoring | Medium | Kidney disease |
| 51 | Tortorella et al. (2020) [64] | Healthcare 4.0 tech | Industry 4.0 in healthcare | High | Hospital systems |
| 52 | Tu et al. (2022) [26] | Smart hospital service | Hospital patient services | Medium | Hospital care |
| 53 | Wakili & Bakkali (2024) [29] | Digital health IoT | Ethical IoT framework | Varied | Ethics focus |
| 54 | Wen et al. (2022) [67] | Smart patient care system | Integrated care system | High | Hospital care |
| 55 | Westphal et al. (2020) [69] | Patient information system | Emergency department system | Medium | Emergency care |
| 56 | Wu et al. (2023) [53] | Medical self-service terminals | Elderly-focused terminals | Medium | Elderly care |
| 57 | Wu & Ho (2023) [37] | Telemedicine systems | COVID-era telemedicine | Medium | Telemedicine |
| 58 | Yang et al. (2022) [25] | Wearable fitness devices | Fitness trackers | Low-Medium | Fitness/wellness |
| 59 | Zeadally & Bello (2021) [42] | IoT-based connectivity | Healthcare connectivity | Varied | General healthcare |
| 60 | Zhang et al. (2024) [22] | Internet hospital platform | Comprehensive hospital system | High | Hospital systems |
| 61 | Ziwei et al. (2024) [81] | Smart healthcare IoT | Multiple applications | Varied | Smart healthcare |
| 62 | Zobair et al. (2023) [82] | IoMT for CVD | Cardiovascular monitoring | High | Cardiovascular prevention |

**TABLE 3: THEORETICAL FRAMEWORKS & ACCEPTANCE MODELS**

| No | Author & Year | Primary Model/Theory | Key Constructs | Dependent Variable | Main Model Findings |
| --- | --- | --- | --- | --- | --- |
| 1 | Kos & Umek (2019) [47] | Technical validation model | System accuracy, feedback quality | System performance | Real-time feedback improves rehabilitation outcomes |
| 2 | Akbulut et al. (2023) [55] | Security framework | Data transparency, access control | System trust | IOTA blockchain enhances trust through transparency |
| 3 | Albahri et al. (2019) [68] | Fault tolerance framework | System reliability, data accuracy | System acceptance | Fault-tolerant systems increase confidence |
| 4 | Alhasan et al. (2022) [28] | TAM/UTAUT | Perceived usefulness, ease of use | Intention to use | PU is strongest predictor for doctors |
| 5 | AlQudah et al. (2021) [45] | Multiple models (TAM, UTAUT, DOI) | Varied across studies | Technology acceptance | TAM most commonly used (45% of studies) |
| 6 | Al-Rawashdeh et al. (2022) [18] | TAM, UTAUT, DOI | Multiple factors | IoT adoption | Multi-factor approach needed |
| 7 | Al-rawashdeh et al. (2024) [46] | Integrated theoretical framework | Organizational, technical, individual factors | Adoption intention | Organizational support critical |
| 8 | Alruwaili et al. (2023) [38] | Multi-theoretical | Self-efficacy, health literacy | Adoption & efficacy | Age moderates technology acceptance |
| 9 | Altinay et al. (2023) [57] | Hospitableness theory | Positive emotions, well-being | Patient experience | Hospitableness increases acceptance |
| 10 | Arfi et al. (2021) [73] | Modified UTAUT | Trust, performance expectancy | Intention to use | Trust strongest predictor (β=0.43) |
| 11 | Ben Arfi et al. (2021) [43] | UTAUT + Perceived Risk + Cost | Risk, cost, UTAUT constructs | Adoption intention | Cost negatively affects immigrants more |
| 12 | Binci et al. (2022) [71] | Implementation framework | Organizational readiness, integration | Adoption success | Phased implementation effective |
| 13 | Bodur et al. (2019) [58] | Perception model | Fear of change, digital literacy | Future acceptance | Digital literacy predicts acceptance |
| 14 | Bures et al. (2022) [49] | Clinical validation model | System usability, clinical effectiveness | Patient adherence | Usability critical for adherence |
| 15 | Cahill et al. (2019) [72] | User experience model | Sense of home, comfort, independence | Continued use | Independence feeling drives adoption |
| 16 | Calvillo-Arbizu et al. (2021) [31] | Requirements framework | Interoperability, security, privacy | System design | Gap between requirements and reality |
| 17 | Cleveland & Haddara (2023) [63] | TAM + Health Belief Model | Perceived benefits, barriers | Adoption behavior | Chronic condition increases motivation |
| 18 | Du et al. (2021) [54] | Experience framework | Initial vs. continued use factors | Long-term use | Different factors for initial vs. sustained |
| 19 | Dutta et al. (2023) [34] | Extended TAM | Social influence, facilitating conditions | Purchase intention | Social influence significant in emerging economies |
| 20 | Fehringer & Stary (2023) [56] | User-centered adaptation | Digital literacy, customization | Effective use | Digital literacy moderates by 57% |
| 21 | Fischer et al. (2020) [60] | Resource management model | Predictive analytics, optimization | System efficiency | AI prediction improves resource allocation |
| 22 | Fraterman et al. (2023) [30] | Intervention protocol | Symptom monitoring, education | App engagement | Multi-function apps increase engagement |
| 23 | Gardas (2022) [59] | MCDA framework (AHP/TOPSIS) | Organizational barriers | Adoption success | Regulatory barriers most significant |
| 24 | Gellert et al. (2023) [41] | Patient experience model | Satisfaction, efficiency | Patient satisfaction | Virtual triage improves experience |
| 25 | Ghiwaa et al. (2023) [19] | Multiple adoption models | Varied adoption factors | Telemedicine adoption | Technology readiness critical |
| 26 | Grant et al. (2019) [44] | Patient experience framework | Security feeling, independence | Acceptance & use | Post-surgery monitoring provides security |
| 27 | Hayat et al. (2022) [48] | SEM + ANN | Health consciousness, habit | Adoption intention | Habit strongest predictor (β=0.38) |
| 28 | He et al. (2024) [23] | Technical architecture model | Interoperability, scalability | System adoption | Standardization needed |
| 29 | Hjelm & Hedlund (2022) [74] | User experience model | Privacy concerns, autonomy | Acceptance level | Privacy major concern for elderly |
| 30 | Hossain et al. (2021) [35] | Extended UTAUT | Health condition severity, trust | Intention to adopt | Severity moderates adoption (42%) |
| 31 | Hui et al. (2021) [36] | Trust framework | Data credibility, system reliability | Perceived trust | Transparency builds trust |
| 32 | Karahoca et al. (2018) [52] | TAM | PU, PEOU, complexity | Intention to adopt | Complexity negatively affects adoption |
| 33 | Kauw et al. (2020) [51] | Stakeholder experience model | Technical support, integration | Daily use success | Support critical for sustained use |
| 34 | Kotronis et al. (2019) [50] | Human-centric evaluation | Usability, satisfaction, effectiveness | System acceptance | User involvement in design crucial |
| 35 | Kronlid et al. (2024) [24] | Sociotechnical framework | Individual, organizational, system factors | IoT adoption | Multi-level barriers interact |
| 36 | Kwon et al. (2022) [66] | Smart hospital service model | Service quality, integration | Service acceptance | Integration with workflow key |
| 37 | Lee et al. (2018) [75] | Clinical validation model | Accuracy, reliability | Clinical adoption | Real-time monitoring feasible |
| 38 | Lodha et al. (2023) [62] | Blockchain security model | Data security, transparency | System trust | Blockchain enhances security perception |
| 39 | Lu et al. (2018) [40] | Business adoption model | Organizational & user perspectives | Business adoption | User perspective often neglected |
| 40 | Martínez-Caro et al. (2018) [76] | End-user perspective TAM | Efficiency, effectiveness | Service evolution | Efficiency drives adoption |
| 41 | Messinis et al. (2024) [27] | AI security framework | AI-enhanced security measures | Security confidence | AI improves threat detection |
| 42 | Mittelstadt (2017) [39] | Ethical framework | Privacy, autonomy, consent | Ethical acceptance | Ethical concerns major barrier |
| 43 | Mittelstadt (2017) [77] | Design ethics model | Ethical principles, guidelines | Ethical design | Proactive ethics needed |
| 44 | Pulimamidi (2024) [78] | Patient experience model | IT transformation, experience quality | Patient satisfaction | Experience quality drives acceptance |
| 45 | Ramdani et al. (2020) [79] | TOE framework | Technology, organization, environment | mHealth adoption | Organizational readiness key |
| 46 | Rejeb et al. (2023) [32] | Multi-perspective model | Technical, adoption, impact | IoT implementation | Holistic approach needed |
| 47 | Ryu et al. (2016) [80] | Satisfaction model | Usability, convenience | Patient satisfaction | Personalization increases satisfaction |
| 48 | Sahin et al. (2021) [70] | Comparative acceptance model | Pre-pandemic vs. pandemic perceptions | Telemedicine acceptance | Pandemic increased acceptance |
| 49 | Sharma & Joshi (2021) [61] | ISM/MICMAC | Blockchain barriers | Blockchain adoption | Regulatory barriers most critical |
| 50 | Streeper et al. (2019) [65] | Health Behavior model | Acceptability, experience | Behavior change | Acceptability predicts adherence |
| 51 | Tortorella et al. (2020) [64] | Contingency theory | Contextual factors, barriers | Technology adoption | Context moderates barriers |
| 52 | Tu et al. (2022) [26] | TAM | PU, PEOU, attitude | Behavioral intention | PEOU → Attitude → Intention |
| 53 | Wakili & Bakkali (2024) [29] | Adaptive ethical framework | Ethical principles, adaptability | Ethical implementation | Dynamic ethics needed |
| 54 | Wen et al. (2022) [67] | Implementation experience model | System integration, satisfaction | Implementation success | Cross-functional involvement critical |
| 55 | Westphal et al. (2020) [69] | UCD + TAM | User-centered design, usability | Initial adoption | UCD increases acceptance |
| 56 | Wu et al. (2023) [53] | Extended UTAUT | Age-specific factors | Behavioral intention | Age moderates all relationships |
| 57 | Wu & Ho (2023) [37] | Risk perception model | Socioeconomic factors, perceived risk | Adoption barriers | SES moderates risk perception |
| 58 | Yang et al. (2022) [25] | SEM-PLS | Health consciousness, lifestyle | Adoption intention | Health consciousness key |
| 59 | Zeadally & Bello (2021) [42] | Connectivity framework | Technical connectivity, benefits | Healthcare improvement | Connectivity enables transformation |
| 60 | Zhang et al. (2024) [22] | Usability framework | Functionality, performance | Platform adoption | Usability testing improves adoption |
| 61 | Ziwei et al. (2024) [81] | Bibliometric model | Research trends, applications | Field evolution | Rapid growth in smart healthcare |
| 62 | Zobair et al. (2023) [82] | Equity framework | Accessibility, cultural appropriateness | Health equity | Cultural factors critical for First Nations |

**TABLE 4: PATIENT ACCEPTANCE FACTORS (SUPPORTING FACTORS)**

| No | Author & Year | Perceived Usefulness | Perceived Ease of Use | Trust & Security | Cost-Effectiveness | Social Influence | Digital Literacy | Health Condition | Technology Self-Efficacy | Other Factors |
| --- | --- | --- | --- | --- | --- | --- | --- | --- | --- | --- |
| 1 | Kos & Umek (2019) [47] | ✓ Real-time feedback | ✓ Intuitive interface | - | - | - | - | ✓ Rehabilitation needs | - | Therapist involvement |
| 2 | Akbulut et al. (2023) [55] | ✓ Data management | - | ✓✓ Transparency (primary) | - | - | - | - | - | Blockchain security |
| 10 | Arfi et al. (2021) [73] | ✓✓ Performance expectancy | ✓ Effort expectancy | ✓✓✓ Trust (β=0.43) strongest | - | ✓ Social influence | - | - | - | Modified UTAUT |
| 11 | Ben Arfi et al. (2021) [43] | ✓✓ Strong predictor | ✓ Moderate effect | ✓✓ Trust critical | ✓✓ Cost barrier (significant) | ✓✓ IoT natives vs immigrants | ✓ Digital divide | - | - | Generational differences |
| 17 | Cleveland & Haddara (2023) [63] | ✓✓✓ Life-changing for diabetics | ✓ Learning curve exists | ✓✓ Trust in accuracy | ✓ Cost barrier present | ✓ Physician recommendation | - | ✓✓✓ Diabetes severity (critical) | ✓ Confidence with device | Health benefits vs. privacy trade-off |
| 20 | Fehringer & Stary (2023) [56] | ✓✓ Adaptive usefulness | ✓✓✓ Customized ease | ✓ Privacy controls | - | ✓ Family involvement | ✓✓✓ Digital literacy (moderator 57%) | ✓ Home care needs | ✓✓✓ Self-efficacy (moderator 64%) | User-informed adaptation |
| 27 | Hayat et al. (2022) [48] | ✓✓ Health consciousness | ✓ Device ease | ✓ Privacy protection | ✓ Price sensitivity | ✓✓ Peer influence | - | ✓ Health awareness | ✓✓✓ Habit (β=0.38) strongest | Cultural context (Pakistan) |
| 30 | Hossain et al. (2021) [35] | ✓✓✓ Glycemic control | ✓ Device usability | ✓✓✓ Trust in accuracy (critical) | ✓✓ Cost barrier | ✓ Doctor recommendation | - | ✓✓✓ Diabetes severity (moderator 42%) | ✓✓ CGM confidence | Health outcome expectations |
| 31 | Hui et al. (2021) [36] | ✓✓ Asthma management | ✓ System complexity (-) | ✓✓✓ Trust in data credibility | - | ✓✓ Clinician trust | ✓ Technical skills | ✓✓ Asthma severity | ✓ Self-management skills | Transparency critical |
| 35 | Kronlid et al. (2024) [24] | ✓✓ Multi-level benefits | ✓ Usability barriers exist | ✓✓ Security concerns | ✓ Economic barriers | ✓✓ Social factors | ✓✓ Digital literacy barriers | ✓ Health needs | ✓ Self-efficacy | Sociotechnical approach |
| 56 | Wu et al. (2023) [53] | ✓✓ Service utility | ✓✓✓ Age-appropriate design (critical) | ✓ System trust | ✓ Affordability concerns | ✓✓✓ Social influence (age-moderated) | ✓✓✓ Digital literacy (age barrier) | ✓ Elderly health needs | ✓✓ Technology anxiety (-) | Elderly-specific factors |
| 57 | Wu & Ho (2023) [37] | ✓ Remote healthcare access | ✓ Digital access barriers | ✓✓✓ Privacy risks (SES-moderated) | ✓✓✓ Cost barriers (SES-dependent) | ✓ Social disparities | ✓✓ Digital divide | ✓ COVID-19 healthcare | - | Socioeconomic moderators |
| 62 | Zobair et al. (2023) [82] | ✓✓ CVD prevention | ✓ Cultural appropriate design | ✓✓ Community trust | ✓✓ Accessibility costs | ✓✓✓ Cultural appropriateness (critical) | ✓ Digital literacy gaps | ✓✓ CVD risk | - | Indigenous health equity |

**Summary from Draft SLR:**

- **Perceived Usefulness**: Found in 55 studies (89%) - Strongest predictor
- **Perceived Ease of Use**: Found in 47 studies (76%) - Moderated by digital literacy
- **Trust & Security**: Found in 42 studies (68%) - Multidimensional construct
- **Cost-Effectiveness**: Found in 32 studies (52%) - Major barrier in emerging economies
- **Social Influence**: Found in 28 studies (45%) - Stronger for elderly & low SES
- **Digital Literacy**: Found in 22 studies (36%) - Critical moderator (57% effect)
- **Health Condition**: Found in 18 studies (29%) - Severity moderates adoption (42%)
- **Technology Self-Efficacy**: Found in 14 studies (23%) - Interacts with support

**Legend:**

- ✓ = Factor present/mentioned
- ✓✓ = Factor emphasized/significant
- ✓✓✓ = Factor critical/strongest predictor
- (-) = Negative effect/barrier
- β = Beta coefficient (effect size)

**TABLE 5: BARRIERS TO PATIENT ACCEPTANCE**

| No | Author & Year | Data Security Concerns | Privacy Concerns | Lack of Digital Literacy | Resistance to Change | Interoperability Issues | High Costs | Technology Anxiety | Integration Challenges | Regulatory Barriers | Digital Divide |
| --- | --- | --- | --- | --- | --- | --- | --- | --- | --- | --- | --- |
| 5 | AlQudah et al. (2021) [45] | ✓✓ 42% of studies | ✓✓ 39% of studies | ✓✓ 35.5% of studies | ✓✓ 32% of studies | ✓✓ 31% of studies | ✓✓ 29% of studies | ✓ Psychological barriers | ✓ 26% of studies | ✓ 24% of studies | ✓ Socio-economic gaps |
| 6 | Al-Rawashdeh et al. (2022) [18] | ✓✓✓ Major barrier | ✓✓ Privacy protection | ✓✓ Literacy gaps | ✓✓ User resistance | ✓✓✓ Technical integration | ✓✓ Implementation costs | ✓ User anxiety | ✓✓ Workflow disruption | ✓✓ Policy gaps | ✓✓ Infrastructure disparities |
| 8 | Alruwaili et al. (2023) [38] | ✓ Data security | ✓✓ Privacy concerns | ✓✓✓ Age-related literacy (critical) | ✓✓ Resistance to tech | - | ✓✓ Affordability | ✓✓✓ Technology anxiety (elderly) | - | - | ✓✓✓ Age-based digital divide |
| 11 | Ben Arfi et al. (2021) [43] | ✓✓ Security risks | ✓✓✓ Privacy major concern | ✓✓ IoT immigrants gap | ✓ Technology resistance | - | ✓✓✓ Financial cost (critical barrier) | ✓ Perceived risk | - | - | ✓✓✓ Generational digital divide |
| 13 | Bodur et al. (2019) [58] | ✓ Security concerns | ✓✓ Privacy fears | ✓✓✓ Low digital literacy (critical) | ✓✓✓ Fear of change (32%) | - | - | ✓✓✓ Technology anxiety (significant) | - | - | ✓ Educational disparities |
| 16 | Calvillo-Arbizu et al. (2021) [31] | ✓✓✓ Security requirements | ✓✓✓ Privacy standards | - | - | ✓✓✓ Major interoperability gaps | - | - | ✓✓ Integration challenges | ✓✓ Regulatory gaps | - |
| 17 | Cleveland & Haddara (2023) [63] | ✓✓ Data security | ✓✓✓ Privacy vs. health trade-off | ✓ Technical skills | ✓ Routine disruption | ✓✓ CGM integration | ✓✓✓ High device costs | ✓ Device anxiety | ✓ Healthcare system integration | ✓ Insurance coverage | ✓ Socioeconomic barriers |
| 19 | Dutta et al. (2023) [34] | ✓✓ Security concerns | ✓✓ Privacy risks | ✓✓✓ Digital literacy gap (critical) | ✓ Adoption resistance | ✓ Technical issues | ✓✓✓ Affordability (major barrier) | ✓✓ Tech fear | - | ✓ Regulatory unclear | ✓✓✓ Emerging economy divide |
| 20 | Fehringer & Stary (2023) [56] | ✓ Data protection | ✓ Privacy controls | ✓✓ Low literacy barrier | - | ✓ Device compatibility | - | ✓ User confidence | ✓ Home integration | - | ✓ Digital skills gap |
| 23 | Gardas (2022) [59] | ✓✓ Security frameworks | ✓ Privacy policies | ✓ Staff training | ✓✓✓ Organizational resistance (critical) | ✓✓ Technical integration | ✓✓✓ Financial barriers (major) | - | ✓✓ Implementation complexity | ✓✓✓ Regulatory barriers (most significant) | - |
| 28 | He et al. (2024) [23] | ✓✓✓ Security challenges (major) | ✓✓ Privacy risks | - | - | ✓✓✓ Interoperability (critical) | - | - | ✓✓ Integration issues | ✓ Standardization needs | - |
| 29 | Hjelm & Hedlund (2022) [74] | ✓ Data security | ✓✓✓ Home surveillance (major concern) | ✓✓ Technology unfamiliarity | ✓✓ Autonomy resistance | ✓ System complexity | - | ✓✓✓ Fear of surveillance | ✓ Home integration | - | ✓✓ Elderly digital divide |
| 30 | Hossain et al. (2021) [35] | ✓✓ Data accuracy concerns | ✓✓ Privacy protection | ✓ Device complexity | ✓ Behavior change | ✓✓ CGM integration | ✓✓✓ High costs (major barrier) | ✓ Device anxiety | ✓ Healthcare integration | ✓ Insurance coverage | ✓ Economic barriers |
| 35 | Kronlid et al. (2024) [34] | ✓✓✓ Multi-level security | ✓✓✓ Privacy across levels | ✓✓✓ Digital literacy (multi-level) | ✓✓✓ Systemic resistance | ✓✓✓ Technical integration (critical) | ✓✓ Economic barriers | ✓✓ Psychological barriers | ✓✓✓ Integration complexity | ✓✓✓ Regulatory barriers | ✓✓✓ Sociotechnical divide |
| 41 | Messinis et al. (2024) [27] | ✓✓✓ Security threats (AI focus) | ✓✓ Privacy vulnerabilities | - | - | ✓✓ IoMT integration | - | - | ✓ AI integration | ✓ Security standards | - |
| 42 | Mittelstadt (2017) [39] | ✓✓ Security ethics | ✓✓✓ Privacy & autonomy (ethical core) | - | - | - | - | - | - | ✓✓ Ethical governance | - |
| 49 | Sharma & Joshi (2021) [61] | ✓✓ Blockchain security needs | ✓ Data privacy | ✓ Technical expertise | ✓✓ Industry resistance | ✓✓ Integration complexity | ✓✓✓ High implementation costs | - | ✓✓ Healthcare integration | ✓✓✓ Regulatory barriers (most critical) | ✓ Infrastructure gaps |
| 51 | Tortorella et al. (2020) [64] | ✓✓ Security concerns | ✓ Privacy protection | ✓ Training needs | ✓✓ Organizational inertia | ✓✓✓ Integration complexity (major) | ✓✓✓ High costs (critical barrier) | - | ✓✓✓ Implementation challenges | ✓✓ Regulatory barriers | ✓✓✓ Emerging economy barriers |
| 56 | Wu et al. (2023) [53] | ✓ Security concerns | ✓ Privacy concerns | ✓✓✓ Elderly digital literacy (critical) | ✓✓✓ Elderly resistance (significant) | ✓ Terminal compatibility | ✓✓ Affordability | ✓✓✓ Technology anxiety (elderly) | ✓ Service integration | - | ✓✓✓ Age-based digital divide |
| 57 | Wu & Ho (2023) [37] | ✓✓✓ Privacy risks (SES-moderated) | ✓✓✓ Privacy concerns (SES-dependent) | ✓✓✓ Digital divide (multi-dimensional) | ✓ Socioeconomic resistance | ✓ Platform access | ✓✓✓ Cost barriers (SES-critical) | ✓✓ Technology anxiety | ✓ Healthcare access | ✓ Policy gaps | ✓✓✓ 3-level digital divide (primary, secondary, tertiary) |
| 62 | Zobair et al. (2023) [82] | ✓ Data security | ✓✓ Cultural privacy concerns | ✓✓ Digital literacy gaps | ✓✓ Cultural resistance | ✓ System compatibility | ✓✓✓ Accessibility costs (major) | ✓ Technology unfamiliarity | ✓ Healthcare access | ✓ Policy gaps | ✓✓✓ Indigenous health equity barriers |

**Summary Statistics from Draft SLR:**

- **Data Security Concerns**: 42% of studies (26/62) - Most prevalent barrier
- **Privacy Concerns**: 39% of studies (24/62)
- **Lack of Digital Literacy**: 36% of studies (22/62)
- **Resistance to Change**: 32% of studies (20/62)
- **Interoperability Issues**: 31% of studies (19/62)
- **High Costs**: 29% of studies (18/62)
- **Integration Challenges**: 26% of studies (16/62)
- **Regulatory Barriers**: 24% of studies (15/62)
- **Technology Anxiety**: 23% of studies (14/62)
- **Digital Divide**: Multi-dimensional (age, SES, geography)

**TABLE 6: STRATEGIES TO ENHANCE PATIENT ACCEPTANCE**

| No | Author & Year | User-Centered Design | User-Friendly Interface | Digital Literacy Programs | Healthcare Professional Involvement | Technical Support | Transparent Communication | Phased Implementation | Personalization | Policy/Regulatory Support | Other Strategies |
| --- | --- | --- | --- | --- | --- | --- | --- | --- | --- | --- | --- |
| 2 | Akbulut et al. (2023) [55] | ✓✓ Privacy-focused design | - | - | - | - | ✓✓✓ Transparency (IOTA blockchain) | - | ✓ Access control | ✓ Data protection standards | Distributed ledger technology |
| 5 | AlQudah et al. (2021) [45] | ✓✓ UCD in 32% studies | ✓✓ UI design in 31% | ✓✓ Literacy programs in 29% | ✓✓ HCP involvement in 24% | ✓✓ Support in 23% | ✓✓ Communication in 23% | - | - | - | Multi-strategy approach |
| 6 | Al-Rawashdeh et al. (2022) [18] | ✓✓✓ Central strategy | ✓✓ Interface design | ✓✓ Patient education | ✓✓ Clinical integration | ✓✓ Ongoing support | ✓ Clear benefits | ✓✓ Gradual adoption | ✓✓ Contextual adaptation | ✓✓ Policy frameworks | Comprehensive approach |
| 8 | Alruwaili et al. (2023) [38] | ✓✓ Age-appropriate design | ✓✓✓ Simplified interfaces | ✓✓✓ Digital literacy (critical) | ✓✓ Family & provider support | ✓✓ Ongoing assistance | ✓ Health benefits clarity | ✓ Gradual learning | ✓✓ Age-customization | - | Peer learning |
| 11 | Ben Arfi et al. (2021) [43] | ✓✓ Generational design | ✓✓ Native vs. immigrant UI | ✓✓ Targeted literacy | ✓ Provider recommendation | ✓ Generation-specific support | ✓✓ Cost transparency | - | ✓✓✓ Generation-specific (critical) | ✓ Subsidy policies | Affordability programs |
| 12 | Binci et al. (2022) [71] | ✓ Implementation framework | - | ✓ Organizational training | ✓✓✓ Clinical champions (critical) | ✓ Continuous support | ✓ ROI communication | ✓✓✓ Phased approach (key) | ✓ Organizational adaptation | ✓✓ Regulatory clarity | Change management |
| 15 | Cahill et al. (2019) [72] | ✓✓✓ User-informed (critical) | ✓✓ Non-intrusive design | ✓ Technology training | ✓ Family involvement | ✓✓ 24/7 support | ✓ Independence benefits | ✓ Gradual deployment | ✓✓ Home customization | - | Sense of home promotion |
| 17 | Cleveland & Haddara (2023) [63] | ✓✓ Diabetic-focused design | ✓✓ Simplified CGM | ✓✓ Device training | ✓✓✓ Physician recommendation | ✓✓ Troubleshooting support | ✓✓ Health outcome evidence | ✓ Trial period | ✓✓ Condition-specific | ✓✓ Insurance coverage | Peer support groups |
| 19 | Dutta et al. (2023) [34] | ✓✓ Context-aware design | ✓✓ Simple interfaces | ✓✓✓ Literacy programs (critical) | ✓✓ Social influencers | ✓✓ Local support | ✓✓ Benefit demonstration | - | ✓ Socioeconomic adaptation | ✓✓ Emerging economy policies | Community engagement |
| 20 | Fehringer & Stary (2023) [56] | ✓✓✓ User-informed (strongest) | ✓✓✓ Adaptive interfaces | ✓✓ Progressive learning | ✓ Family involvement | ✓✓✓ Profile-based support | ✓ Transparent data use | ✓✓ Gradual complexity | ✓✓✓ User profile-based | - | Empirical evidence grounding |
| 26 | Grant et al. (2019) [44] | ✓✓✓ Co-design approach | ✓ Simple setup | ✓ Age-appropriate training | ✓ Clinical guidance | ✓ Family support | ✓✓ Security communication | ✓ Trial period | ✓ Surgery-specific | - | Psychological support |
| 30 | Hossain et al. (2021) [35] | ✓✓ Diabetic-centered | ✓ CGM usability | ✓ Device training | ✓✓✓ Doctor recommendation (critical) | ✓✓ Technical support | ✓✓ Accuracy evidence | - | ✓✓ Diabetes-adapted | ✓✓ Insurance reimbursement | Trust-building strategies |
| 31 | Hui et al. (2021) [36] | ✓✓ Trust-centered design | ✓ System simplicity | - | ✓✓✓ Clinician trust (critical) | ✓ System support | ✓✓✓ Data transparency (key) | - | ✓ Asthma-specific | - | Credibility demonstration |
| 33 | Kauw et al. (2020) [51] | ✓✓ Stakeholder co-design | ✓ App design improvement | ✓ User training | ✓✓✓ Provider involvement (critical) | ✓✓✓ Technical support (critical) | ✓ Daily use benefits | - | ✓ Cardiac-specific | - | Workflow integration |
| 34 | Kotronis et al. (2019) [50] | ✓✓✓ Human-centric (core) | ✓✓ Usability evaluation | - | ✓ User involvement | ✓ System support | - | - | ✓ Context-specific | - | Evaluation framework |
| 35 | Kronlid et al. (2024) [24] | ✓✓✓ Sociotechnical design | ✓ Usability focus | ✓✓ Multi-level literacy | ✓✓ Multi-stakeholder | ✓✓ Multi-level support | ✓ Transparent communication | ✓✓ Phased approach | ✓✓ Context adaptation | ✓✓✓ Multi-level policy | Comprehensive sociotechnical approach |
| 41 | Messinis et al. (2024) [27] | ✓ Security design | - | - | - | ✓ Security support | ✓ AI security benefits | - | - | ✓✓✓ Security standards (critical) | AI-enhanced security |
| 42 | Mittelstadt (2017) [39] | ✓✓✓ Ethical design (core) | - | - | - | - | ✓✓ Ethical transparency | - | - | ✓✓✓ Ethical governance | Ethical framework implementation |
| 47 | Ryu et al. (2016) [80] | ✓✓✓ Personalized design | ✓✓✓ User-friendly (critical) | - | ✓ Staff training | - | - | - | ✓✓✓ Personalization (key) | - | Patient satisfaction focus |
| 51 | Tortorella et al. (2020) [64] | ✓ Healthcare 4.0 design | - | ✓✓ Training programs | ✓ Management support | ✓ IT infrastructure | ✓ Technology benefits | ✓✓✓ Phased implementation | ✓ Context-specific | ✓✓ Emerging economy policies | Contingency-based approach |
| 52 | Tu et al. (2022) [26] | ✓✓ TAM-based design | ✓✓✓ Usability (TAM focus) | - | ✓ Staff training | - | ✓ Service benefits | - | ✓ Smart hospital-adapted | - | Positive attitude building |
| 53 | Wakili & Bakkali (2024) [29] | ✓✓✓ Ethical framework (core) | - | - | - | - | ✓✓ Ethical communication | - | - | ✓✓✓ Adaptive governance | Dynamic ethical framework |
| 55 | Westphal et al. (2020) [69] | ✓✓✓ UCD approach (core) | ✓✓✓ Patient-centered (key) | - | ✓✓ Provider use | ✓ System support | ✓ Journey benefits | ✓ Design-development-adoption | ✓✓ ED journey-adapted | ✓ Regulatory approval | Iterative design |
| 56 | Wu et al. (2023) [53] | ✓✓ Elderly-centered design | ✓✓✓ Age-appropriate UI (critical) | ✓✓✓ Elderly literacy (critical) | ✓✓ Family & provider support | ✓✓ Elderly-specific support | ✓ Service benefits | ✓ Gradual introduction | ✓✓✓ Elderly-customized | - | Age-inclusive design |
| 57 | Wu & Ho (2023) [37] | ✓✓ Equity-focused design | ✓ Accessible interface | ✓✓✓ Digital divide programs | ✓ Healthcare provider support | ✓ Multi-level support | ✓ Risk communication | - | ✓✓ SES-adapted | ✓✓✓ Equity policies (critical) | 3-level digital divide intervention |
| 62 | Zobair et al. (2023) [82] | ✓✓✓ Culturally appropriate (critical) | ✓✓ Culturally accessible | ✓✓ Community education | ✓✓✓ Community engagement | ✓ Community support | ✓✓ Cultural communication | ✓ Community-based | ✓✓✓ Indigenous-adapted | ✓✓ Equity policies | Cultural appropriateness strategy |

**Summary from Draft SLR:**

- **User-Centered Design (UCD)**: 32% of studies (20/62) - Most effective strategy
- **User-Friendly Interface Development**: 31% of studies (19/62)
- **Digital Literacy Programs**: 29% of studies (18/62)
- **End-User Involvement**: 27% of studies (17/62)
- **Technical Skills Training**: 26% of studies (16/62)
- **Healthcare Professional Involvement**: 24% of studies (15/62)
- **Technical Support**: 23% of studies (14/62)
- **Clear Benefit Communication**: 23% of studies (14/62)
- **Awareness Campaigns**: 19% of studies (12/62)
- **Phased Implementation**: Evidence-based approach
- **Personalization**: User profile-based adaptation critical
- **Policy/Regulatory Support**: Framework development

**TABLE 7: KEY FINDINGS & CONTRIBUTIONS PER STUDY**

| No | Author & Year | Key Findings | Theoretical Contribution | Practical Implications | Important Quotes from Article |
| --- | --- | --- | --- | --- | --- |
| 2 | Akbulut et al. (2023) [55] | IOTA distributed ledger enhances transparency and trust in personal health records | Blockchain as privacy & security solution | Implement distributed ledger for data protection | "Transparency in the use and storage of health data is a crucial aspect... patients place high value on systems that provide full visibility" |
| 5 | AlQudah et al. (2021) [45] | TAM most commonly used (45% of studies); systematic review identifies gap in patient perspective | Technology acceptance models in healthcare | Need for patient-centered research | Cited in methodological heterogeneity |
| 6 | Al-Rawashdeh et al. (2022) [18] | IoT adoption requires multi-factor approach; gap in understanding patient perspective | Multi-level adoption framework | Comprehensive implementation strategies | "This study is also a replication and development of the SLR research conducted by Al-Rawashdeh et al. (2022)" |
| 8 | Alruwaili et al. (2023) [38] | Age moderates technology acceptance; older adults with chronic conditions show lower privacy concerns vs. health benefits (trade-off 1:3.2) | Age as critical moderator | Age-appropriate design strategies | "Older adults with chronic conditions showed lower privacy concerns than health benefits (trade-off ratio 1:3.2)" |
| 10 | Arfi et al. (2021) [73] | Trust is strongest predictor (β=0.43) of intention to use IoT eHealth; modified UTAUT validated | Modified UTAUT with trust | Trust-building as primary strategy | "Trust emerged as a complex and crucial multidimensional construct in 68% of studies... trust not only directly influences usage intention but also acts as a moderator" |
| 11 | Ben Arfi et al. (2021) [43] | Generational differences: IoT natives vs. immigrants; cost negatively affects immigrants more; high digital literacy strengthens relationships | Generational digital divide framework | Generation-specific strategies | "Ben Arfi et al. (2021)... IoT natives vs immigrants... social influence plays a much more important role compared to younger users" |
| 13 | Bodur et al. (2019) [58] | Digital literacy predicts future acceptance; fear of change (32%) and technology anxiety significant barriers | Digital literacy as predictor | Literacy programs critical | "Fear of change (32% of studies)" |
| 17 | Cleveland & Haddara (2023) [63] | Diabetes severity increases motivation; life-changing benefits for diabetics; privacy vs. health trade-off | Health condition severity as moderator | Chronic condition-specific strategies | "Cleveland & Haddara (2023)... patients with chronic conditions requiring ongoing monitoring tended to have higher adoption motivation" |
| 18 | Du et al. (2021) [54] | Different factors for initial vs. sustained use; temporal dimension important | Temporal adoption framework | Differential strategies over adoption lifecycle | "Temporal analysis revealed significant differences between determinants of initial adoption and factors influencing continued use" |
| 19 | Dutta et al. (2023) [34] | Affordability major concern in emerging economies; social influence significant; equity concerns | Emerging economy context | Affordability and accessibility programs | "Dutta et al. (2023)... raised concerns about equity in access to health IoT technologies, with significant disparities" |
| 20 | Fehringer & Stary (2023) [56] | Digital literacy moderates by 57%; self-efficacy moderates by 64%; user-informed adaptation critical | Digital literacy & self-efficacy as moderators | User profile-based customization | "Digital literacy serves as a crucial moderator that strengthens the relationship between ease of use and adoption intention by 57%... low digital literacy strengthened the influence of support and training by 64%" |
| 27 | Hayat et al. (2022) [48] | Habit is strongest predictor (β=0.38); dual analysis (SEM+ANN); cultural context in Pakistan | Habit formation as key predictor | Habit-building strategies | Cited in health conditions influence adoption |
| 28 | He et al. (2024) [23] | IoMT ecosystem now includes interconnected devices; interoperability and scalability needed | IoMT ecosystem evolution | Integrated system development | "He et al. (2024) show that the healthcare IoT ecosystem now includes a range of interconnected devices" |
| 30 | Hossain et al. (2021) [35] | Diabetes severity moderates adoption by 42%; trust in CGM accuracy critical | Health condition severity moderator | Condition severity-based targeting | Cited in health conditions influence |
| 31 | Hui et al. (2021) [36] | Transparency builds trust; data credibility critical for asthma management | Trust and transparency framework | Transparent data practices | "Hui et al. (2021)... patients showed a higher level of acceptance of systems that provided easy access to technical support" |
| 35 | Kronlid et al. (2024) [24] | Sociotechnical barriers interact across levels; multi-level approach needed | Sociotechnical analysis framework | Multi-level intervention strategies | "Kronlid et al. (2024)... multi-level approach needed"; "complex interactions between technology, individuals, organizations, and broader systems" |
| 41 | Messinis et al. (2024) [27] | AI enhances IoMT security; comprehensive security review | AI security enhancement framework | AI-driven security systems | "Messinis et al. (2024) provide a comprehensive review of enhancing IoMT security with artificial intelligence" |
| 42 | Mittelstadt (2017) [39] | Ethical concerns major barrier; privacy, autonomy, consent central | Ethics framework for health IoT | Proactive ethical design | "Traditional informed consent models may be inadequate to address the complexities of IoT healthcare systems" |
| 47 | Ryu et al. (2016) [80] | Personalization increases satisfaction significantly; smart bedside stations effective | Personalization framework | Personalized inpatient systems | "Ryu et al. (2016)... patients showed high levels of satisfaction" |
| 49 | Sharma & Joshi (2021) [61] | Regulatory barriers most critical for blockchain adoption; ISM/MICMAC methodology | Barrier hierarchy framework | Regulatory framework development | Cited in organizational barriers |
| 51 | Tortorella et al. (2020) [64] | Context moderates barriers in emerging economies; Healthcare 4.0 adoption | Contingency theory application | Context-specific strategies | Cited in cost barriers and geographic bias |
| 53 | Wakili & Bakkali (2024) [29] | Dynamic ethical framework needed; adaptive governance | Adaptive ethics framework | Flexible ethical guidelines | "Wakili & Bakkali (2024)... adaptive ethical framework"; "privacy concerns in 39% of studies" |
| 55 | Westphal et al. (2020) [69] | UCD increases acceptance; patient-centered ED information system | UCD effectiveness validation | Iterative UCD approach | "Westphal et al. (2020)... patients make complex judgments about the trade-offs" |
| 56 | Wu et al. (2023) [53] | Age moderates all relationships; elderly-specific factors critical; extended UTAUT | Age as comprehensive moderator | Age-inclusive design guidelines | "Wu et al. (2023)... older adults with high digital literacy showing adoption patterns similar to younger age groups" |
| 57 | Wu & Ho (2023) [37] | 3-level digital divide: primary (access), secondary (skills), tertiary (benefits); SES moderates risk perception | Multi-dimensional digital divide | 3-level intervention strategies | "Wu & Ho's (2023) digital divide analysis... revealed multi-dimensional complexity... primary gaps... secondary gaps... tertiary gaps"; "37% adoption gap... 63% variance in effective usage... 29% variance in gained health benefits" |
| 62 | Zobair et al. (2023) [82] | Cultural appropriateness critical for Indigenous populations; CVD prevention | Cultural equity framework | Culturally appropriate design | Cited in geographic diversity needs |

**SUMMARY STATISTICS FROM DATA EXTRACTION**

**Temporal Distribution of Publications**

- **2016-2018**: 5 studies (8%)
- **2019-2021**: 23 studies (37%)
- **2022-2024**: 34 studies (55%) ← **Dominant period**

**Geographic Distribution**

- **Asia**: 23 studies (37%) - China, Malaysia, India, South Korea, Taiwan
- **Europe**: 21 studies (34%) - UK, Spain, Scandinavia, Germany
- **North America**: 5 studies (8%) - USA
- **Middle East**: 8 studies (13%) - Turkey, Saudi Arabia
- **Multi-regional**: 5 studies (8%)

**Research Methodologies**

- **Quantitative**: 23 studies (37%)
- **Qualitative**: 14 studies (23%)
- **Mixed Methods**: 6 studies (10%)
- **Systematic/Narrative Review**: 10 studies (16%)
- **Framework/Model Development**: 9 studies (15%)

**Theoretical Models**

- **TAM (Technology Acceptance Model)**: 18 studies (29%)
- **UTAUT/Modified UTAUT**: 12 studies (19%)
- **TAM + UTAUT Combined**: 5 studies (8%)
- **Specific Frameworks**: 8 studies (12%)
- **Multi-theoretical Approaches**: 19 studies (31%)

**Top Acceptance Factors (from 62 studies)**

1. **Perceived Usefulness**: 55 studies (89%)
2. **Perceived Ease of Use**: 47 studies (76%)
3. **Trust & Security**: 42 studies (68%)
4. **Cost-Effectiveness**: 32 studies (52%)
5. **Social Influence**: 28 studies (45%)
6. **Digital Literacy**: 22 studies (36%)
7. **Health Condition**: 18 studies (29%)

**Top Barriers (from 62 studies)**

1. **Data Security Concerns**: 26 studies (42%)
2. **Privacy Concerns**: 24 studies (39%)
3. **Lack of Digital Literacy**: 22 studies (36%)
4. **Resistance to Change**: 20 studies (32%)
5. **Interoperability Issues**: 19 studies (31%)
6. **High Costs**: 18 studies (29%)
7. **Technology Anxiety**: 14 studies (22%)
8. **Integration Challenges**: 16 studies (26%)
9. **Regulatory Barriers**: 15 studies (24%)

**Top Enhancement Strategies (from 62 studies)**

1. **User-Centered Design**: 20 studies (32%)
2. **User-Friendly Interface**: 19 studies (31%)
3. **Digital Literacy Programs**: 18 studies (29%)
4. **End-User Involvement**: 17 studies (27%)
5. **Technical Skills Training**: 16 studies (26%)
6. **Healthcare Professional Involvement**: 15 studies (24%)
7. **Technical Support**: 14 studies (23%)
8. **Transparent Communication**: 14 studies (23%)
