# Supplementary material for: Patient Acceptance and Barriers to IoT Usage in Health Care: Systematic Literature Review
Source: JMIR Mhealth Uhealth. 2026 Jul 31;14:e81260. doi: 10.2196/81260 (PMC13430414; doi:10.2196/81260)
Supplement: Multimedia Appendix 4 — Summary of methodological quality appraisal (Mixed Methods Appraisal Tool Assessment). [file mhealth-v14-e81260-s004.pdf]

Summary of Methodological Quality (MMAT Assessment)

MMAT Screening Questions

All 62 included studies successfully passed the two MMAT screening questions, indicating that each study had a clear research question and appropriate data to address it. In other words, 100% of studies scored “Yes” on both screening items. None of the included studies failed the screening stage, as those not meeting these basic criteria would have been excluded. This uniform strength at the screening level means that every study sets a solid foundation with well-defined objectives and relevant data sources, an important precondition for further quality appraisal.

Qualitative Studies

Fourteen studies (approximately 23% of the sample) used qualitative designs. Common strengths in this domain were clarity of purpose and appropriate methodology. **All 14 qualitative studies (100%) had a clearly stated research aim** and aligned qualitative approach (MMAT criterion 1: 14/14 “Yes”). Almost all also used suitable qualitative methods for data collection and analysis (criterion 2 was “Yes” in 13 studies, 93%). Over half (8 studies, ~57%) adequately described their sampling or data sources (criterion 3 “Yes”), though the remaining had only unclear information on this point.

However, notable weaknesses emerged in later criteria. Only **3 out of 14 qualitative studies (21%) fully met criterion 4**, which typically assesses the coherence of data analysis and whether findings are supported by the data. The majority (10 studies) were rated “Can’t tell” on this aspect, indicating insufficient detail or transparency in how interpretations were derived. **None of the qualitative studies explicitly addressed researcher bias or reflexivity (criterion 5: 0% “Yes”)**, with 6 studies even marked “No” for this item. This suggests that discussions of the researchers’ influence on data or critical reflexivity were largely absent. As a result of these gaps, no qualitative study achieved all five MMAT criteria, and the best-performing qualitative papers still fell short on at least one criterion (in fact, the top three qualitative studies met 4 out of 5 criteria, with criterion 5 remaining unfulfilled). Overall, qualitative studies tended to be strong in articulating their aims and methods, but weak in reporting rigor of analysis and reflexivity.

Quantitative Studies

Twenty-three studies (~37% of the sample) were quantitative in design (e.g. surveys or experiments). These studies showed robust performance on early appraisal criteria. **All 23 quantitative studies (100%) had a clear research question and appropriate quantitative design** (criteria 1 and 2: 23/23 “Yes” for each). Most also defined their sampling, measurements, or intervention procedures well – **18 studies (78%) satisfied criterion 3**, while the remaining 5 had some ambiguity (marked “Can’t tell”). This indicates that the majority of quantitative studies had an appropriate and well-described methodology up to the point of data collection.

On the other hand, many quantitative studies did not fully address later quality criteria regarding data completeness and bias. Only **8 of 23 (35%) clearly met criterion 4**, which often relates to whether outcome data are complete or whether follow-up/nonresponse issues are handled. The majority (15 studies) were rated “Can’t tell” on criterion 4, suggesting that information on attrition, missing data, or outcome completeness was usually not reported in enough detail. **Criterion 5 was the weakest area** – only 2 quantitative studies (9%) received a “Yes” on this final item, whereas 16 (70%) were “Can’t tell” and 5 (22%) were “No” on criterion 5. This last criterion typically concerns whether the study accounted for confounding factors or potential biases (such as nonresponse bias in surveys); the fact that 91% of quantitative studies did not convincingly meet this suggests that many did not adequately discuss or mitigate bias. Notably, **only two quantitative studies (Bodur et al., 2019; Ziwei et al., 2024) achieved “Yes” on all five MMAT criteria for their design**. This represents just 9% of the quantitative subset (and about 3% of all included studies). Most quantitative studies, therefore, had one or more methodological limitations (usually related to incomplete outcome/bias reporting), but generally they were strong in fundamental design quality.

Mixed Methods Studies

Six studies (~10% of the sample) employed mixed methods designs. In this domain, a clear strength was that all mixed-methods studies were well-justified in their basic design. **All 6 had a clear research question and rationale for using mixed methods (criterion 1: 100% “Yes”)**. Five of six (83%) also used an appropriate mixed-methods design and data collection procedure (criterion 2 “Yes” in 5 studies), indicating that most combined qualitative and quantitative

components in a reasonable way. Additionally, **four studies (67%) adequately described each component's sampling or measurements (criterion 3 "Yes")**, while two had some unclear details.

The major challenges appeared in how well the studies integrated and interpreted the mixed data. **Only 4 out of 6 (67%) clearly met criterion 4**, which for mixed methods often evaluates the extent of integration or whether the qualitative and quantitative results are effectively brought together to answer the research question. One study failed this outright ("No"), and another was unclear, suggesting that a minority did not effectively merge their two strands of data. **None of the mixed methods studies fully satisfied criterion 5 (0% "Yes")**, with 4 marked "Can't tell" and 2 marked "No" on this item. In mixed-method appraisals, the final criterion typically examines whether conclusions appropriately incorporate both qualitative and quantitative findings and consider limitations. The lack of any "Yes" here indicates that **integration and reflexive consideration of limitations were consistently weak** in these studies. No mixed-methods study achieved all five criteria; the best attained 4 out of 5. In summary, mixed methods studies were generally sound in design and execution of each component, but **the integration of findings and thorough discussion of biases/limitations were lacking** across the board.

Other Study Designs (Frameworks and Systematic Reviews)

In addition to primary studies, the review included 9 papers proposing conceptual frameworks and 10 systematic review articles (together ~31% of the included works). These did not fall neatly into standard MMAT design categories, but they were appraised on relevant criteria. **All nine framework papers had well-defined objectives and methodology (criteria 1–3 all "Yes")**. However, since these were not empirical studies with data, **every framework study was marked "Can't tell" for criteria 4 and 5**. This reflects that aspects like data-derived results or discussions of bias were not applicable or not clearly reported in these conceptual works. In other words, while the conceptual studies were strong in rationale and design, their lack of empirical data meant certain quality criteria could not be fully evaluated (a consistent limitation).

The ten **included systematic reviews** were generally assessed favorably on initial criteria but showed mixed quality in later criteria. All 10 had clearly formulated research questions and inclusion methods (criterion 1: 100% "Yes"). **Nine of ten (90%) employed appropriate and**

systematic methodologies (criterion 2 "Yes"), with one review unclear in its method reporting. About **60% of the systematic reviews (6 out of 10) provided a thorough data synthesis or meta-analysis (criterion 3 "Yes")**, while the rest lacked clarity on how findings were derived (marked "Can't tell"). Similarly, **6 reviews (60%) adequately addressed the quality of included studies or heterogeneity (criterion 4 "Yes")**, but 3 were unclear and 1 failed to do so. **Notably, none of the systematic reviews fully met the final criterion (criterion 5: 0% "Yes")**, which likely pertains to consideration of biases or limitations in the review – in fact 4 reviews did not meet this and the other 6 were unclear on it. This indicates that **even well-conducted reviews often did not explicitly discuss limitations or the impact of study quality on their conclusions**. Overall, the **systematic reviews tended to be methodologically sound (many followed PRISMA guidelines, as noted in their reports)**, but **all lacked a completely comprehensive discussion of bias/limitations**, keeping any one review from scoring a perfect five "Yes" on MMAT.

Implications for Confidence and Interpretability

The MMAT assessment of these 62 studies reveals a mixed methodological picture that directly influences how confident we can be in the review's findings. On one hand, **every included study had a solid fundamental design and purpose**, as evidenced by universal passing of screening questions and high adherence to initial quality criteria across qualitative, quantitative, and mixed methods designs. In fact, the majority of studies (73%) were rated by the reviewers as having at least "good" overall quality. These strengths suggest that most evidence in the review is based on studies with reasonably sound methods, lending a baseline of credibility to the findings.

On the other hand, **nearly all studies exhibited some methodological limitations**, often related to incomplete reporting or risk of bias. Very few studies were flawless **– only 2 out of 62 (3%) met all five MMAT criteria** for their design. Around one-third of studies (34%) came close, meeting four criteria, but fully **two-thirds had three or fewer criteria rated "Yes," indicating at least two domains of concern per study**. Common weak points (e.g., lack of reflexivity in qualitative research, unaddressed nonresponse or confounding in quantitative studies, and insufficient integration in mixed methods) mean that for most individual studies, certain findings must be interpreted with caution. The fact that **criterion 5 (addressing biases/limitations) was**

rarely fulfilled across all designs is particularly noteworthy – it implies that many studies did not openly account for potential biases, which could overestimate the strength of evidence.

For the systematic review as a whole, these quality issues temper our confidence in the conclusions. Because many included studies had unclear or low ratings on some criteria, the evidence base includes uncertainties. For example, if qualitative studies did not consider researcher bias, their themes might be less trustworthy; if surveys did not address nonresponse bias, their results might not generalize well. However, since most studies were still of at least moderate quality (with 45 studies judged "good" or "excellent" by the authors), the review's overall findings remain useful – they are drawn from generally robust studies, albeit not perfect ones. The implication is that the review's conclusions are **likely valid in broad strokes**, but any specific result should be interpreted considering the noted methodological gaps. The presence of consistent weaknesses (e.g., lack of thorough bias consideration) suggests that **there may be an over-optimism in some findings**, and the review authors wisely conducted sensitivity analyses focusing on higher-quality studies to ensure key results were robust. In summary, the MMAT assessment indicates a need for caution: the evidence supports the review's insights, but the **interpretability and certainty of those insights are moderate** rather than absolute. The systematic review's strength is bolstered by many good-quality studies, yet critical appraisal reminds us to interpret its outcomes with an understanding of the prevalent methodological limitations.
