# Supplementary material for: Patient Acceptance and Barriers to IoT Usage in Health Care: Systematic Literature Review
Source: JMIR Mhealth Uhealth. 2026 Jul 31;14:e81260. doi: 10.2196/81260 (PMC13430414; doi:10.2196/81260)
Supplement: Multimedia Appendix 5 — Strategies for enhancing patient acceptance of internet of things in health care(version 1.2). [file mhealth-v14-e81260-s005.docx]

**Summary of Evidence-Based Strategies for Enhancing Patient Acceptance of IoT in Healthcare**

Systematic Literature Review: Patient Acceptance and Barriers to IoT Utilization in Healthcare (ms#81260)

N = 62 included studies as universal denominator. Multi-coding applied; a single study may appear in more than one category. Percentages do not sum to 100%.

| **Level** | **Strategy** | **Description & Implementation** | **Primary Evidence Sources** | **N Studies (%)** | **Evidence Confidence** |
| --- | --- | --- | --- | --- | --- |
| **A. Individual-Level Strategies** | | | | | |
| Individual | **User-Centered Design (UCD)** | Most frequently identified strategy (N=20, 32%). Core elements consistently described:  • Participatory design workshops with patients, caregivers, and healthcare providers  • Iterative prototyping with representative users in realistic clinical scenarios  • User journey mapping and co-design sessions across diverse patient populations  • Usability testing with particular attention to elderly users and lower digital literacy populations  Spans diverse IoT modalities and study designs across multiple geographic contexts. | **Primary refs: [45], [18], [38], [56], [24], [69]**  AlQudah et al. (2021) [45]  Al-Rawashdeh et al. (2022) [18]  Alruwaili et al. (2023) [38]  Fehringer & Stary (2023) [56]  Kronlid et al. (2024) [24]  Westphal et al. (2020) [69]  Full supporting list also includes [26,29,34,35,36,37,39,43,44,50,51,53,63,64,71,72,80,82] | 20 (32%) | **HIGH (consistent across studies spanning multiple IoT modalities and geographic contexts)** |
| Individual | **User-Friendly Interface Design** | Second most identified strategy (N=19, 31%). Design principles:  • Clear visual hierarchy and consistent design patterns minimising cognitive burden  • Minimal text with clear, universally understood icons across literacy levels  • Responsive and accessible design for wearable devices and elderly users  • Customisation options accommodating diverse individual needs and preferences  Particularly emphasised in studies focused on elderly populations and lower digital literacy users. | **Primary refs: [45], [38], [56], [53]**  AlQudah et al. (2021) [45]  Alruwaili et al. (2023) [38]  Fehringer & Stary (2023) [56]  Wu et al. (2023) [53]  Full supporting list also includes [18,24,26,34,37,43,44,50,51,63,69,72,80,82] | 19 (31%) | **HIGH (replicated across distinct IoT device categories; studies predominantly assess usability preferences)** |
| Individual | **Digital Literacy Programs** | Third most identified strategy (N=18, 29%). Programs consistently address:  • Basic device operation: setup, pairing, and troubleshooting  • Data interpretation skills: understanding readings and health implications  • Progressive skill-building with gradual complexity introduction  • Multi-modal delivery: workshops, peer learning, and online tutorials | **Primary refs: [38], [43], [63], [34], [56], [53]**  Alruwaili et al. (2023) [38]  Ben Arfi et al. (2021) [43]  Cleveland & Haddara (2023) [63]  Dutta et al. (2023) [34]  Fehringer & Stary (2023) [56]  Wu et al. (2023) [53]  Full supporting list also includes [18,24,37,45,64,82] | 18 (29%) | **HIGH (consistent across 6+ studies and diverse geographic contexts)** |
| Individual | **User Empowerment & Shared Decision-Making** | N=17 studies (27%). Strategies include:  • Shared decision-making frameworks giving patients control over data use  • Transparency dashboards showing data collection, use, and storage purposes  • Granular consent mechanisms and privacy controls  • Goal-setting features with personalised health targets | **Primary refs: [45], [18], [63], [56], [24]**  AlQudah et al. (2021) [45]  Al-Rawashdeh et al. (2022) [18]  Cleveland & Haddara (2023) [63]  Fehringer & Stary (2023) [56]  Kronlid et al. (2024) [24]  Full supporting list also includes [34,38,43,72,82] | 17 (27%) | **MODERATE (consistent themes across studies; varied measurement approaches limit aggregation)** |
| Individual | **Personalization & Profile-Based Adaptation** | N=16 studies (26%). Approaches consistently described:  • Interface adaptation based on digital literacy profile and health condition  • Health content adjustment based on specific chronic disease context  • Adaptive complexity scaling to user proficiency over time  • Cultural and language adaptation for diverse populations | **Primary refs: [38], [56], [80], [53]**  Alruwaili et al. (2023) [38]  Fehringer & Stary (2023) [56]  Ryu et al. (2016) [80]  Wu et al. (2023) [53]  Full supporting list also includes [18,24,34,37,43,45,63,69,82]  . | 16 (26%) | **MODERATE (evidence present across diverse contexts; generalisation across IoT device categories limited)** |
| Individual | **Trust-Building Mechanisms** | N=14 studies (23%). Strategies include:  • Transparent communication about data collection, use, and storage purposes  • Security certifications and audit trail visibility for users  • Clear protocols for data breach notification and response  • Provider endorsement and clinical validation of IoT systems | **Primary refs: [66], [79], [42]**  Kwon et al. (2022) [66]  Ramdani et al. (2020) [79]  Zeadally & Bello (2021) [42]  Full supporting list also includes [18,24,36,39,45,55,73] | 14 (23%) | **MODERATE (trust mechanism well-documented)** |
| **B. Organizational-Level Strategies** | | | | | |
| Organizational | **Multi-Stakeholder Involvement in Design** | N=15 studies (24%). Mechanisms consistently described:  • Interdisciplinary design teams: clinicians, IT professionals, patients, administrators  • Patient Advisory Boards contributing to system priority-setting  • Community health worker involvement particularly in underserved areas  • Iterative feedback loops embedded throughout development and deployment | **Primary refs: [45], [18], [24], [82]**  AlQudah et al. (2021) [45]  Al-Rawashdeh et al. (2022) [18]  Kronlid et al. (2024) [24]  Zobair et al. (2023) [82]  Full supporting list also includes [34,35,38,51,63,71] | 15 (24%) | **MODERATE-HIGH (robust conceptual consensus; few primary RCTs on implementation outcomes)** |
| Organizational | **Clinical Workflow Integration** | N=16 studies (26%). Strategies include:  • Workflow analysis prior to deployment to identify friction points  • Healthcare provider co-design minimising workflow disruption  • Alert and reminder systems embedded into existing clinical routines  • Reduction of duplicate data entry through EHR integration | **Primary refs: [45], [18], [48], [24]**  AlQudah et al. (2021) [45]  Al-Rawashdeh et al. (2022) [18]  Hayat et al. (2022) [48]  Kronlid et al. (2024) [24]  Full supporting list also includes [35,51,63,64,66,71] | 16 (26%) | **MODERATE-HIGH (consistent across implementation-focused studies; evidence predominantly observational)** |
| Organizational | **Champion & Peer-Influencer Programs** | N=12 studies (19%). Models described include:  • Clinical champions advocating for IoT adoption among peers  • Train-the-trainer cascades to reach larger populations  • Peer patient ambassadors in community-based settings  • Social proof mechanisms through observed peer use and testimonials | **Primary refs: [45], [18], [71]**  AlQudah et al. (2021) [45]  Al-Rawashdeh et al. (2022) [18]  Binci et al. (2022) [71]  Full supporting list also includes [24,64,79] | 12 (19%) | **MODERATE (social influence mechanism well-established; implementation program evaluations limited)** |
| Organizational | **Phased & Evidence-Based Implementation** | N=11 studies (18%). Approaches include:  • Pilot programs with small cohorts before full-scale rollout  • Iterative evaluation cycles with feedback-informed adjustments  • Staged deployment across clinical departments or geographic areas  • Implementation science frameworks guiding scale-up decisions | **Primary refs: [71], [24], [64], [82]**  Binci et al. (2022) [71]  Kronlid et al. (2024) [24]  Tortorella et al. (2020) [64]  Zobair et al. (2023) [82]  Full supporting list also includes [18,45] | 11 (18%) | **MODERATE (logically and empirically supported; few controlled implementation studies)** |
| **C. System-Level Strategies** | | | | | |
| System | **Interoperability Standards & Open Infrastructure** | Most frequently cited system-level strategy (N=19, 31%). Components:  • Adoption of interoperability standards enabling cross-platform data exchange  • Open APIs enabling third-party integration across platforms  • Health information exchanges enabling cross-institutional data sharing  • Edge computing infrastructure for time-sensitive clinical applications | **Primary refs: [45], [18], [44], [76]**  AlQudah et al. (2021) [45]  Al-Rawashdeh et al. (2022) [18]  Grant et al. (2019) [44]  Martínez-Caro et al. (2018) [76]  Full supporting list also includes [23,24,31] | 19 (31%) | **HIGH (technical consensus across included studies; widely endorsed in implementation and policy literature)** |
| System | **Security & Privacy Architecture** | N=17 studies (27%). Technical and governance strategies:  • End-to-end encryption of all transmitted patient health data  • Distributed ledger and blockchain-based audit trails for data access  • Regulatory compliance frameworks aligned with applicable laws  • Transparent privacy policies presented in accessible language | **Primary refs: [55], [44], [62], [61]**  Akbulut et al. (2023) [55]  Grant et al. (2019) [44]  Lodha et al. (2023) [62]  Sharma & Joshi (2021) [61]  Full supporting list also includes [18,23,24,27,31,37,39,45] | 17 (27%) | **HIGH (technical approaches well-established; patient-perceived security as adoption driver remains less studied)** |
| System | **Clear Regulatory & Approval Frameworks** | N=13 studies (21%). Policy mechanisms described:  • Streamlined approval pathways for lower-risk IoT health devices  • Risk-based regulatory classification reducing barriers for non-invasive monitoring  • National digital health strategies providing governance certainty for developers  • Standardised clinical validation requirements for IoT diagnostic claims | **Primary refs: [45], [59], [39], [29]**  AlQudah et al. (2021) [45]  Gardas (2022) [59]  Mittelstadt (2017) [39]  Wakili & Bakkali (2024) [29]  Full supporting list also includes [18,24,31,61,64,77] | 13 (21%) | **MODERATE (policy rationale consistently strong; empirical evaluation of framework impact limited)** |
| System | **Financial Incentives & Reimbursement Policies** | N=11 studies (18%). Mechanisms described:  • Reimbursement policies covering IoT device costs for eligible patients  • Value-based care models incorporating remote monitoring outcomes  • Public procurement frameworks ensuring competitive and accessible pricing | **Primary refs: [45], [43], [36]**  AlQudah et al. (2021) [45]  Ben Arfi et al. (2021) [43]  Hui et al. (2021) [36]  Full supporting list also includes [34,37,63,64,77,82] | 11 (18%) | **MODERATE (cost barrier consistently documented; impact of specific incentive models requires further evidence)** |
| System | **Insurance Coverage & Preventive Care Programs** | N=9 studies (15%). Approaches described:  • Insurance coverage policies for approved IoT monitoring devices  • Preventive care incentives tied to active device use and health targets  • Subsidised device provision programmes for low-income or high-risk populations | **Primary refs: [45], [43], [35], [51]**  AlQudah et al. (2021) [45]  Ben Arfi et al. (2021) [43]  Hossain et al. (2021) [35]  Kauw et al. (2020) [51]  Full supporting list also includes [37,63,77,82]  . | 9 (15%) | **MODERATE (equity rationale strong; outcomes of specific insurance program models under-studied)** |
| System | **Digital Equity & Infrastructure Development** | Cross-cutting strategy addressing multi-dimensional digital divide (N=12, 19%):  • Broadband and connectivity expansion in underserved rural and peri-urban areas  • Subsidised device provision for low-income populations  • Community health worker-mediated technology access models  • Three-level digital divide interventions: primary access, secondary skills, tertiary benefits [37] | **Primary refs: [45], [38], [37], [82]**  AlQudah et al. (2021) [45]  Alruwaili et al. (2023) [38]  Wu & Ho (2023) [37]  Zobair et al. (2023) [82]  Full supporting list also includes [18,24,34,43,53,74] | 12 (19%) | **MODERATE-HIGH (gap evidence strong and consistently documented; intervention evidence on equity programs emerging)** |
| All percentages calculated out of N = 62 included studies. Multi-coding applied; a single study may appear in more than one strategy category; cumulative percentages exceed 100% by design. Verified against Multimedia Appendix 3 (Study Characteristics) and Multimedia Appendix 4 (Data Extraction Tables). Evidence Confidence ratings reflect study count, methodological diversity, and MMAT quality distribution (Multimedia Appendix 5). | | | | | |
