# Supplementary material for: Patient Acceptance and Barriers to IoT Usage in Health Care: Systematic Literature Review
Source: JMIR Mhealth Uhealth. 2026 Jul 31;14:e81260. doi: 10.2196/81260 (PMC13430414; doi:10.2196/81260)
Supplement: Checklist 1 — PRISMA 2020 27-Item Checklist V1.2. [file mhealth-v14-e81260-s007.pdf]

Checklist PRISMA 2020 27-Item Updated
Systematic Literature Review: *"Patient Acceptance and Barriers to IoT Utilization in Healthcare: A Systematic Literature Review"*
Manuscript ID: 81260
Journal: JMIR mHealth and uHealth

TITLE
Item 1: Title
Requirement: Identify the report as a systematic review
Compliance: ✓ COMPLIANT
Response: The title clearly states this is a *Systematic Literature Review*. Title format structure addressing intervention, population, and study design explicitly.
Evidence:

- **Revised Title:** "Patient Acceptance and Barriers to IoT Utilization in Healthcare: A Systematic Literature Review"
- Format follows JMIR guidelines: "Issue/Intervention in Population: Method/Study Design"
- Clearly identifies: Population (patients), Intervention (IoT in healthcare), Method (systematic literature review)
- Document type explicitly stated as "Systematic Literature Review"

ABSTRACT
Item 2: Abstract
Requirement: Provide a structured summary
Compliance: ✓ COMPLIANT
Response: The abstract has been rewritten as a structured abstract (Background, Objectives, Methods, Results, Conclusions). Abstract expanded, fully structured, aligned across all subsections with absolute numbers provided for all percentages.
Evidence:

- **Structured Abstract** with required subsections:
    - o **Background:** IoT transformation paradigm, implementation gaps, research rationale
    - o **Objective:** Clear statement of aims to identify and synthesize acceptance factors, barriers, and enhancement strategies
    - o **Methods:** PRISMA 2020 guidelines, 8 databases searched (PubMed/MEDLINE, Scopus, IEEE Xplore, Web of Science, ScienceDirect, ACM Digital Library, ProQuest, Google Scholar), January 2016-December 2024, inclusion/exclusion criteria, quality assessment with MMAT, thematic analysis
    - o **Results:** Complete quantitative reporting: 2,537 initially identified → 62 included studies; geographic distribution (Asia 37%, Europe 34%, North

America 8%, Middle East 13%); methodological approaches (quantitative 37%, qualitative 24%, mixed methods 11%, systematic reviews 19%); quality assessment (45 studies/73% good-to-excellent); key findings with absolute numbers (perceived usefulness 55/62 studies [89%], ease of use 47/62 [76%], trust 42/62 [68%], security concerns 26/62 [42%], privacy issues 24/62 [39%], digital literacy gaps 22/62 [36%])
- **Conclusions:** Evidence-based implications for multi-level interventions, equity considerations, future research priorities

INTRODUCTION
Item 3: Rationale
Requirement: Describe the rationale for the review in the context of existing knowledge
Compliance: ✓ COMPLIANT
Response: The introduction explains why a patient-focused IoT acceptance review is important and identifies key research gaps. Added comparison subsection clarifying specific contributions beyond prior systematic reviews.
Evidence:

- Clear research gap identified: "Most healthcare IoT research remains dominated by focus on technical aspects... while patient perspectives as end users and factors influencing their acceptance still receive inadequate attention"
- High abandonment rates cited: "Up to 50% of wearable health device users discontinue use within first six months" (Attig & Franke, 2022)
- Gap between technical advancement and sustainable adoption documented
- Limited comprehensive synthesis of patient perspectives acknowledged

New Addition: "This Review's Added Value" subsection explicitly contrasts this work with three closest prior reviews:

- Al-Rawashdeh et al. (2022): 60 studies, multiple stakeholders vs. our exclusive patient focus
- Shanbehzadeh et al. (2022): 45 studies, clinical decision support only vs. our IoT healthcare scope
- Brar et al. (2022): 38 studies, wearables only vs. our full IoT spectrum

Distinctive Contributions: Exclusive patient perspective focus, IoT healthcare scope, barrier and strategy synthesis, rigorous quality assessment integration, larger and more recent evidence base (62 studies, 2016-2024.

Item 4: Objectives
Requirement: Provide an explicit statement of the objective(s) or question(s) the review addresses
Compliance: ✓ COMPLIANT

Response: The review objectives are explicitly stated and framed using a PICO-like structure. Research questions explicitly stated and mapped to results structure, with answer summaries provided at end of each relevant results subsection.
Evidence:

- **Three Explicit Research Questions stated at end of Introduction:**

RQ1: What factors facilitate patient acceptance of IoT technology in healthcare?
RQ2: What barriers hinder patient acceptance of IoT technology in healthcare?
RQ3: What strategies enhance patient acceptance of IoT technology in healthcare?
PICO Framework Alignment:

- **Population:** Patients using or potentially using IoT-based healthcare services
- **Intervention:** IoT technology integration in healthcare services
- **Comparison:** Various acceptance determinants and contexts
- **Outcome:** Patient acceptance levels, adoption patterns, continued use

METHODS
Item 5: Eligibility Criteria
Requirement: Specify the inclusion and exclusion criteria for the review
Compliance: ✓ COMPLIANT
Response: Eligibility criteria are reported and justified with rationale for each component, including timeframe, language, study types, and topic scope.
Evidence:
Inclusion Criteria:

1. **Publication Period:** January 2016 – December 2024 (rationale: captures period when IoT healthcare technology matured and became widely adopted)
2. **Language:** English or Indonesian
3. **Research Focus:** Empirical studies explicitly investigating IoT technology integration in healthcare services with primary/ significant focus on patient perspectives, experiences, or attitudes
4. **Study Design:** Quantitative, qualitative, mixed methods
5. **Publication Type:** Peer-reviewed journal articles
6. **Outcome:** Studies analyzing/ exploring factors influencing acceptance, adoption, or continued use of healthcare IoT technology by patients

Exclusion Criteria:

1. Studies primarily focusing on IoT technical aspects (architecture, protocols, algorithms) without patient perspectives
2. Studies focusing only on healthcare provider, administrator, or staff perspectives
3. Opinion articles, editorials, comments, non-systematic reviews
4. Conference abstracts without full text
5. Research protocols without empirical results

Item 6: Information Sources
Requirement: Specify all databases, registers, websites, organizations, reference lists and other sources searched or consulted
Compliance: ✓ COMPLIANT
Response: The manuscript lists the main electronic databases and search timeframe.
Evidence:
Electronic Databases (8 total):
PubMed/MEDLINE, Scopus, IEEE Xplore, Web of Science, ScienceDirect, ACM Digital Library, ProQuest, Google Scholar
Search Timeframe: January 2016 to December 2024

Item 7: Search Strategy
Requirement: Present the full search strategies for all databases, registers and websites, including any filters and limits used
Compliance: ✓ COMPLIANT
Response: The manuscript reports the search approach and keyword logic, full database-specific strings are provided with example and full documentation in Multimedia Appendix to ensure reproducibility.
Evidence:
Search Strategy Development: Search strategy developed iteratively combining three concept groups:

1. **IoT/Technology Terms:** "Internet of Things," "IoT," "connected health," "smart health," "telemedicine," "telehealth," "mHealth," "eHealth," "wearable devices"
2. **Healthcare Terms:** "healthcare," "patient," "medical," "clinical," "health services," "hospital," "clinic," "home care"
3. **Acceptance Terms:** "acceptance," "adoption," "readiness," "willingness," "perception," "attitude," "experience," "engagement," "trust," "retention," "barriers," "facilitators"

Boolean Operators: AND, OR combined concept groups with database-specific adaptations for syntax and field tags.
Filters: 2016-2024, English OR Indonesian, Journal Article

Item 8: Selection Process
Requirement: State the process for selecting studies (screening, eligibility)
Compliance: ✓ COMPLIANT
Response to Reviewers: Complete selection process described with screening roles, and categorized exclusion reasons.
Evidence: Three-Stage Selection Process:
Stage 1: Deduplication
Stage 2: Title/Abstract Screening
Stage 3: Full-Text Screening

Item 9: Data Collection Process
Requirement: Describe the methods used to collect data and to extract or code data
Compliance: ✓ COMPLIANT
Response: The manuscript describes standardized extraction steps and extracted domains.
Evidence:
Standardized Extraction Process:
Extraction Form Development:

- Piloted on diverse studies (quantitative, qualitative, mixed methods)
- Finalized extraction template with 8 categories

Data Extraction Categories:

1. **Bibliographic Information:** Authors, year, country, journal, DOI
2. **Study Design & Methodology:** Research design, sample size, data collection methods, analysis approaches
3. **Participant Characteristics:** Demographics, health conditions, settings
4. **IoT Technology Types:** Wearables, remote monitoring, IoMT, mHealth/eHealth
5. **Theoretical Frameworks:** TAM, UTAUT, HBM, integrated models
6. **Facilitating Factors:** Identified with prevalence across studies
7. **Barriers:** Security, privacy, literacy, resistance, interoperability, costs
8. **Enhancement Strategies:** Design, education, support, policy interventions
9. **Quality Assessment Criteria:** MMAT scores and quality ratings

Extraction Procedure:

- **Primary Extraction:** First author extracted all data using standardized form
- **Verification:** All extracted quantitative data (prevalence percentages, sample sizes, effect sizes) systematically verified against original source documents
- **Team Review:** Extraction tables reviewed by research team for completeness, consistency, accuracy
- **Ambiguity Resolution:** Flagged unclear information discussed among authors to reach consensus on categorization

Data Organization:

- Extraction tables created for all 62 studies
- Organized by research question themes
- Available as **Multimedia Appendix 3** (Study Characteristics Table) and **Appendix 4** (Complete Extraction Data)

Item 10: Data Items
Requirement: List and define all outcomes for which data were sought, and any assumptions made about data not reported
Compliance: ✓ COMPLIANT
Response: The data items include study characteristics, IoT context, acceptance factors, barriers, and reported outcomes.

Evidence:

Primary Outcomes Extracted:

1. **Facilitating Factors (RQ1):**
   - Perceived usefulness (operational definition: patient perception that technology improves health outcomes/quality of life)
   - Perceived ease of use (complexity, learning curve, interface usability)
   - Trust (in technology reliability, data security, healthcare providers)
   - Cost-effectiveness (value perception, affordability)
   - Social support (family, healthcare provider endorsement)
2. **Barriers (RQ2):**
   - Data security concerns (unauthorized access, cyber attacks)
   - Privacy issues (data collection, sharing, surveillance)
   - Digital literacy gaps (technical skills, understanding)
   - Resistance to change (psychological, behavioral inertia)
   - Interoperability issues (device/system compatibility)
   - High costs (purchase, subscription, maintenance)
3. **Enhancement Strategies (RQ3):**
   - User-centered design approaches
   - User-friendly interface development
   - Digital literacy programs
   - Healthcare professional involvement
   - Technical support provision
   - Clear benefit communication

Item 11: Study Risk of Bias Assessment

Requirement: Specify the methods used to assess risk of bias in the included studies

Compliance: ✓ COMPLIANT

Response: The manuscript reports quality appraisal tools (MMAT instrument explicitly named) suitable for different study designs; appraisal was performed consistently across included studies.

Evidence:

Quality Assessment Instrument:

- **Mixed Methods Appraisal Tool (MMAT)** used for all studies
- MMAT chosen because it provides design-appropriate modules for quantitative, qualitative, and mixed methods research

MMAT Assessment Domains:

Quality Rating Categories:

- **Excellent (100%):** All criteria met
- **Good (75-99%):** Most criteria met with minor limitations
- **Fair (50-74%):** Some criteria met with moderate limitations
- **Poor (<50%):** Few criteria met with major limitations

Assessment Results:

- **Good-to-Excellent Quality:** 45 studies (73%)
- **Fair Quality:** 14 studies (23%)
- **Poor Quality:** 3 studies (5%)

Assessment Procedure:

- **Documentation:** MMAT ratings for all studies in **Multimedia Appendix 5**

Integration into Synthesis:

- Quality considerations integrated into narrative synthesis
- **Sensitivity Analysis:** Findings remained robust when limited to good-to-excellent quality studies (n=45)
- Quality limitations explicitly discussed in "Limitations" section

Item 12: Effect Measures

Requirement: Specify for each outcome the effect measure(s) used in the synthesis

Compliance: ✓ COMPLIANT

Response: Because included studies are heterogeneous, effect measures were summarized mainly using thematic/narrative synthesis and structured counting of reported factors when applicable. Effect measures clearly specified for each outcome type with explanation of how heterogeneous data were standardized for synthesis.

Evidence:

Effect Measures by Data Type:

1. Prevalence/Frequency Measures:

- **Primary Metric:** Percentage of studies identifying each factor as significant
- **Calculation:** (Number of studies identifying factor / 62 total studies) × 100
- **Example:** Perceived usefulness identified in 55/62 studies (89%)

2. Standardization Approach:

- **Heterogeneity Acknowledged:** Different studies used different scales and measures
- **Common Metric:** Prevalence percentages for comparability
- **Narrative Synthesis:** For findings not amenable to quantitative pooling

Item 13: Synthesis Methods

Requirement: Describe the processes used to decide which studies were eligible for each synthesis

Compliance: ✓ COMPLIANT

Response: The review uses narrative synthesis and thematic grouping to aggregate acceptance factors, barriers, and strategies. SWiM-compliant narrative synthesis described, vote-counting limitations acknowledged, subgroup and sensitivity analyses specified, visual synthesis tools employed.

Evidence:

Synthesis Approach:

- **Primary Method:** Structured narrative synthesis following SWiM (Synthesis Without Meta-analysis) guidelines
- **Rationale:** Meta-analysis not feasible due to heterogeneity in outcome measures, study designs, IoT technologies, and populations

Synthesis Structure:

1. By Research Question:

- **RQ1 Synthesis:** Facilitating factors grouped thematically (technology perceptions, trust, support, context)
- **RQ2 Synthesis:** Barriers categorized by level (individual/micro, organizational/meso, system/macro)
- **RQ3 Synthesis:** Strategies organized by intervention type (design, education, support, policy)

2. Thematic Analysis Process:

- **Step 1:** Initial coding of factors identified in each study
- **Step 2:** Grouping codes into preliminary themes
- **Step 3:** Refining themes through team discussion
- **Step 4:** Mapping themes to research questions
- **Step 5:** Identifying patterns, relationships, and variations

3. Quantitative Synthesis Elements:

- **Vote Counting:** Prevalence percentages with absolute numbers
- **Effect Direction:** Consistently positive/negative/mixed findings
- **Magnitude Ranking:** Ordering factors by frequency and reported strength

4. Subgroup Analyses:

- **Geographic:** Asia vs. Europe vs. North America vs. Middle East
- **Technology Type:** Wearables vs. remote monitoring vs. IoMT vs. mHealth
- **Clinical Domain:** Chronic disease vs. general health vs. acute care
- **Age Group:** Elderly-focused vs. general adult vs. mixed populations

5. Sensitivity Analyses:

- **Quality:** Limiting to good-to-excellent quality studies (n=45)
- **Design:** Separating quantitative from qualitative findings
- **Recency:** 2022-2024 studies vs. 2016-2021 studies

6. Integration Across Studies:

- **Convergence:** Identified consistent findings across multiple studies
- **Divergence:** Highlighted contextual variations and contradictions

Synthesis Limitations Acknowledged:

- Vote counting does not account for study sample size or effect magnitude
- Studies weighted equally regardless of quality in frequency counts
- Construct definitions varied across studies (carefully documented)

Visual Synthesis Tools:

- **Figure 5:** Theoretical framework distribution over time
- **Figure 7:** Barriers heat map by clinical domain

- **Figure 8:** Multi-level enhancement strategies framework

Software:

- **Analysis:** Microsoft Excel 365 for frequency tabulation
- **Visualization:** Microsoft Visio 365 and Microsoft Excel 365

Item 14: Reporting Bias Assessment

Requirement: Describe any methods used to assess risk of bias due to missing results in a synthesis

Compliance: ✓ COMPLIANT

Response: The manuscript discusses risks such as publication bias and heterogeneity and their implications, language and geographic biases acknowledged, impact on synthesis interpreted cautiously, mitigation strategies employed.

Evidence:

Publication Bias Assessment:

1. Conceptual Assessment:

- **Positive Results Bias:** Studies with significant positive findings more likely to be published than null findings
- **Language Bias:** English/Indonesian-only search may miss studies in other languages
- **Database Indexing Bias:** Some relevant studies may not be indexed in searched databases
- **Gray Literature Exclusion:** Conference proceedings, dissertations, technical reports not systematically searched beyond Google Scholar

2. Methodological Patterns Examined:

- **Temporal Trends:** Examined publication patterns over time (55% of studies from 2022-2024)
- **Geographic Distribution:** Assessed regional representation (71% from Asia and Europe, underrepresentation of Africa and Latin America)
- **Design Distribution:** Analyzed methodological approaches

3. Quality and Heterogeneity Impact:

- **Methodological Heterogeneity:** Variations in construct definitions and measurement approaches across studies
- **Measurement Variability:** Different scales and instruments used to assess constructs
- **Population Selection:** Many studies recruited from engaged populations, potentially overestimating acceptance

4. Evidence Certainty Assessment:

- **Main Findings:** Moderate certainty given consistency across multiple studies but limited by observational designs
- **Subgroup Findings:** Low-to-moderate certainty due to smaller sample sizes and greater heterogeneity

- **Temporal Patterns:** Low certainty due to limited longitudinal evidence

5. Mitigation Strategies:

- **Comprehensive Search:** Eight databases plus supplementary searches
- **No Results Filter:** Included studies regardless of findings direction
- **Quality Integration:** Sensitivity analyses excluding lower-quality studies
- **Transparent Reporting:** All limitations explicitly discussed

Impact on Synthesis:

- Findings may be optimistic bias toward acceptance
- Barriers may be underreported relative to true prevalence
- Implementation challenges likely underrepresented
- Negative experiences and failures may be missing

Item 15: Certainty Assessment

Requirement: Describe any methods used to assess certainty in the body of evidence

Compliance: ✓ COMPLIANT

Response: The manuscript describes additional analyses only where applicable and supported; if not conducted, this is explicitly stated. Certainty assessment conducted across multiple domains, overall ratings provided for key findings, factors affecting certainty explicitly discussed.

Evidence:

Certainty Assessment Framework:

Domains Evaluated:

1. Consistency:

- **High Consistency:** Perceived usefulness (89% of studies), ease of use (76%)
- **Moderate Consistency:** Trust (68%), cost considerations (52%)
- **Low Consistency:** Specific enhancement strategies (high variability)

2. Directness:

- **Direct Evidence:** 62 studies directly addressing patient acceptance of IoT healthcare
- **Indirect Elements:** Limited intervention studies, mostly observational
- **Population Relevance:** Studies generally representative of intended patient populations

3. Study Quality:

- **Good-Excellent:** 45/62 studies (73%)
- **Fair-Poor:** 17/62 studies (27%)
- **Sensitivity Analysis:** Findings robust when limited to high-quality studies

Overall Certainty Ratings:

High Certainty Findings:

- Perceived usefulness is primary acceptance determinant (consistent across 89% of studies with strong effect sizes)

- Digital literacy significantly moderates acceptance (consistent evidence from multiple high-quality studies)

Moderate Certainty Findings:

- Ease of use influences adoption (consistent but effect size varies by population)
- Privacy/security concerns are barriers (present but importance varies by context)
- User-centered design enhances acceptance (limited intervention evidence)

Low Certainty Findings:

- Specific threshold values for factors (high heterogeneity)
- Comparative effectiveness of strategies (limited head-to-head comparisons)
- Long-term sustainability patterns (limited longitudinal evidence)

Factors Reducing Certainty:

- Predominance of cross-sectional designs (causality uncertain)
- Measurement heterogeneity (construct drift)
- Geographic bias (generalizability limited)
- Selection bias (engaged populations overrepresented)

Factors Increasing Certainty:

- Large number of studies (62 total)
- Consistent main findings across contexts
- Multiple theoretical frameworks converging
- Qualitative-quantitative triangulation

RESULTS

Item 16: Study Selection

Requirement: Describe the results of the search and selection process

Compliance: ✓ COMPLIANT

Response: Study selection is reported using a PRISMA flow diagram and counts at each stage, categorized exclusion reasons, deduplication process described.

Evidence:

PRISMA 2020 Flow Diagram (Figure 2): Complete with all required elements

Temporal Distribution:

- 2016-2018: 5 studies (8%)
- 2019-2021: 23 studies (37%)
- 2022-2024: 34 studies (55%)

Item 17: Study Characteristics

Requirement: Present characteristics of each study including citation, study design, and population

Compliance: ✓ COMPLIANT

Response: Complete characteristics table provided in appendix with narrative summary covering all key dimensions including geographic, methodological, technological, and quality distributions.

Evidence:

Study Characteristics Table (Multimedia Appendix 3) includes for all 62 studies:

- Citation (authors, year, journal, DOI)
- Country and geographic region
- Study design (quantitative/qualitative/mixed/review)
- Sample size and characteristics
- Clinical domain and health conditions
- IoT technology type
- Theoretical framework employed
- Key outcomes measured
- Quality rating (MMAT score)

Narrative Summary Characteristics:

1. Geographic Distribution (n=62):

- Asia: 23 studies (37%) - China, Malaysia, India, South Korea, Taiwan
- Europe: 21 studies (34%) - UK, France, Spain, Germany, Scandinavia
- North America: 5 studies (8%) - United States, Canada
- Middle East: 8 studies (13%) - Turkey, Saudi Arabia, UAE
- Multi-regional/Other: 5 studies (8%)

2. IoT Technology Types:

- Wearable sensors/personal monitoring: 12 studies (19%)
- Remote health monitoring systems: 10 studies (16%)
- Internet of Medical Things (IoMT): 8 studies (13%)
- mHealth/eHealth applications: 7 studies (11%)
- Smart hospital systems: 6 studies (10%)
- Telemedicine platforms: 5 studies (8%)
- Multiple/integrated systems: 14 studies (23%)

3. Clinical Domains:

- Chronic disease management: 18 studies (29%)
- General healthcare: 15 studies (24%)
- Elderly care: 10 studies (16%)
- Cardiovascular conditions: 7 studies (11%)
- Diabetes management: 6 studies (10%)
- Multiple conditions: 6 studies (10%)

4. Theoretical Frameworks:

- Technology Acceptance Model (TAM): 18 studies (29%)
- Unified Theory of Acceptance and Use of Technology (UTAUT): 12 studies (19%)
- TAM + UTAUT combinations: 5 studies (8%)
- Integrated/hybrid models: 8 studies (13%)
- Health Belief Model (HBM): 4 studies (7%)
- Other/no explicit framework: 15 studies (24%)

Item 18: Risk of Bias in Studies

Requirement: Present assessments of risk of bias for each included study
Compliance: ✓ COMPLIANT
Response: Quality appraisal outcomes are reported, and their implications for interpretation are described. MMAT ratings for all 62 studies in appendix, bias patterns identified, impact on synthesis explicitly addressed through sensitivity analyses.
Evidence:
Quality Assessment Summary:
Overall Quality Distribution (using MMAT):

- **Good-to-Excellent Quality:** 45 studies (73%)
    - Excellent (100% criteria met): 12 studies (19%)
    - Good (75-99% criteria met): 33 studies (53%)
- **Fair Quality (50-74% criteria met):** 14 studies (23%)
- **Poor Quality (<50% criteria met):** 3 studies (5%)

Cross-Study Bias Concerns:

- **Publication Bias:** Likely overrepresentation of positive findings
- **Language Bias:** English/Indonesian only, potentially missing other language studies
- **Geographic Bias:** Underrepresentation of developing countries and non-Western contexts
- **Population Selection Bias:** Many studies recruited engaged/technology-accepting participants
- **Measurement Heterogeneity:** Diverse definitions and measures of same constructs

Impact on Synthesis:

- **Sensitivity Analysis:** Main findings remained consistent when limited to 45 good-to-excellent quality studies
- **Certainty Ratings:** Adjusted based on quality and bias concerns
- **Interpretations:** Qualified with acknowledgment of bias patterns

Complete Individual Study Ratings: Available in **Multimedia Appendix 5** (Quality Assessment Table) with domain-specific MMAT ratings for each study

Item 19: Results of Individual Studies
Requirement: For all outcomes, present for each study: summary statistics and effect estimate with confidence interval
Compliance: ✓ COMPLIANT (with limitations acknowledged)
Response: Individual study results are summarized within thematic categories with representative examples including effect sizes, confidence intervals where available, and sample sizes for all studies. Full detailed extraction is provided in supplementary material.
Evidence:
Synthesis of Individual Study Findings:

Note on Meta-Analysis: Individual effect estimates with confidence intervals not pooled quantitatively due to heterogeneity in measures, scales, and populations. Findings synthesized narratively with prevalence reporting.

RQ1: Facilitating Factors - Key Individual Study Results:

- **Perceived Usefulness (55/62 studies, 89%):**
- **Perceived Ease of Use (47/62 studies, 76%):**
- **Trust and Security (42/62 studies, 68%):**

RQ2: Barriers - Key Individual Study Results:

- **Data Security Concerns (26/62 studies, 42%):**
- **Privacy Issues (24/62 studies, 39%):**
- **Digital Literacy Gaps (22/62 studies, 36%):**

RQ3: Enhancement Strategies - Key Individual Study Results:

- **User-Centered Design (20/62 studies, 32%):**
- **Digital Literacy Programs (18/62 studies, 29%):**

Complete Individual Study Data: Organized in thematic tables in **Multimedia Appendix 3** with all available effect estimates, sample sizes, and key findings

Item 20: Results of Syntheses

Requirement: For each synthesis, briefly summarize the characteristics and risk of bias among contributing studies

Compliance: ✓ COMPLIANT

Response: The review summarizes key facilitators (eg, usefulness, ease of use, trust) and barriers (eg, privacy/security concerns) across included studies. Synthesis presented for each RQ with study characteristics, bias summaries, key findings, heterogeneity assessment, and certainty ratings. Cross-synthesis patterns identified.

Evidence:

Synthesis 1: Facilitating Factors (RQ1)

Contributing Studies: 62 studies total, all contributed to facilitating factors analysis

Synthesis 2: Barriers (RQ2)

Contributing Studies: 62 studies total, 48 explicitly examined barriers (77%)

Synthesis 3: Enhancement Strategies (RQ3)

Contributing Studies: 42 studies (68%) explicitly examined or recommended enhancement strategies

Overall Evidence Base:

- **Strengths:** Large number of studies (n=62), consistent main findings, diverse contexts
- **Limitations:** Cross-sectional designs dominate, measurement heterogeneity, geographic bias, limited intervention evidence

Item 21: Reporting Biases

Requirement: Present assessments of risk of bias due to missing results for each synthesis
Compliance: ✓ COMPLIANT
Response: Publication bias assessment presented, multiple bias sources identified with quantitative indicators where possible, impact on synthesis interpreted, mitigation strategies documented.
Evidence:
Publication Bias Assessment:
Observed Patterns Suggesting Potential Bias:

1. **Predominance of Positive Findings:**
    - **Facilitating Factors:** All 62 studies identified at least one facilitator
    - **Successful Implementations:** 47/62 studies (76%) reported generally positive attitudes
    - **Failed Implementations:** Only 3 studies (5%) explicitly examined technology abandonment
    - **Implication:** Potential underrepresentation of failed implementations and negative experiences
2. **Language Bias:**
    - **Search Limitation:** English and Indonesian only
    - **Missing:** Potential studies in Chinese (major IoT research country), Spanish, Arabic, German, Portuguese
    - **Impact:** May miss cultural variations and region-specific findings, particularly from Latin America, Middle East (non-English), and East Asia
3. **Database Coverage Bias:**
    - **Indexed Literature:** Focus on peer-reviewed journals
    - **Gray Literature:** Limited systematic search (Google Scholar only)
    - **Missing:** Conference proceedings, dissertations, technical reports, implementation case studies
    - **Implication:** Industry implementations and practical failures may be underrepresented
4. **Geographic Publication Patterns:**
    - **Well-Represented:** Asia (37%), Europe (34%)
    - **Underrepresented:** Africa (0%), Latin America (0%), Oceania (0%)
    - **Implication:** Findings may not generalize to low-resource settings, different healthcare systems, diverse cultural contexts
5. **Outcome Reporting Bias:**
    - **Frequently Reported:** Perceived usefulness, ease of use (standard TAM/UTAUT constructs)
    - **Less Reported:** Long-term outcomes, actual usage data, implementation costs, adverse events

- **Implication:** May overemphasize initial attitudes over sustained adoption and real-world effectiveness

Impact on Synthesis Findings:

Likely Overestimated:

- Overall acceptance levels (selection of engaged populations)
- Effectiveness of interventions (successful implementations more likely published)
- Ease of implementation (challenges underreported)

Likely Underestimated:

- Prevalence and severity of barriers (failures underreported)
- Technology abandonment rates (few longitudinal studies)
- Implementation costs and resource requirements (rarely reported)
- Equity gaps and digital divide impacts (underrepresented populations)

Mitigation Strategies Employed:

1. **Comprehensive Search:** Eight databases plus supplementary searches
2. **No Results Filter:** Included studies regardless of findings direction
3. **Quality Assessment:** MMAT applied to all studies, sensitivity analyses conducted
4. **Critical Interpretation:** Findings interpreted with caution, limitations explicitly discussed
5. **Balanced Reporting:** Both facilitators and barriers systematically synthesized

Transparency Statement: All limitations and potential biases explicitly acknowledged in manuscript. Readers cautioned that findings may represent "best-case scenarios" and real-world implementation challenges may be greater than literature suggests.

Item 22: Certainty of Evidence

Requirement: Present assessments of certainty for each main outcome

Compliance: ✓ COMPLIANT

Response: The manuscript discusses certainty limitations due to heterogeneity, dominance of observational designs, and geographic coverage imbalance. Certainty assessment using adapted GRADE framework, ratings provided for all main outcomes, supporting evidence and reasoning documented, implications for interpretation specified.

Evidence:

Certainty Assessment by Main Outcome:

Framework: Adapted GRADE approach considering study design, risk of bias, inconsistency, indirectness, imprecision, and publication bias

Overall Evidence Base Certainty: MODERATE for main findings (facilitators and barriers), **LOW** for intervention effectiveness

DISCUSSION

Item 23: Discussion

Requirement: Provide a general interpretation of the results in the context of other evidence

Compliance: ✓ COMPLIANT

Response: The discussion summarizes the main findings, compares them with prior literature, and highlights practical implications for IoT implementation and patient-centered design. Discussion integrating findings with existing theory and evidence, novel contributions highlighted, implications explicitly linked to supporting evidence, research gaps identified.

Evidence:

Main Findings Integration with Existing Evidence:

Confirmation and Extension of Technology Acceptance Theory:

Our findings confirm core TAM constructs (Davis, 1989) while revealing healthcare-specific nuances:

- **Perceived usefulness** consistently strongest predictor (89% of studies) - aligns with TAM but stronger than typical consumer technology (usually 60-70%)
- **Healthcare-specific trust** emerged as additional critical construct (68% of studies) - extends basic TAM to address unique privacy/safety concerns
- **Digital literacy moderating effects** (34% of studies) - extends understanding beyond original TAM assumptions of homogeneous users

Integration with UTAUT: Our synthesis supports Venkatesh et al.'s (2003) expanded model:

- Performance expectancy (usefulness): ✓ Confirmed
- Effort expectancy (ease of use): ✓ Confirmed, especially for elderly
- Social influence: Present but less emphasized than expected (only 23% of studies)
- Facilitating conditions: ✓ Confirmed through technical support findings (23% of studies)

Theoretical Evolution Documented:

Our synthesis reveals **progression in theoretical sophistication**:

- **2016-2018:** Simple TAM applications (perceived usefulness + ease of use)
- **2019-2021:** UTAUT extensions adding context (social influence, facilitating conditions)
- **2022-2024: Integrated socio-technical frameworks** recognizing multi-level influences (individual, organizational, system)

Implication: Field maturing from individual psychological models toward **implementation science frameworks** addressing real-world complexity

Item 24: Limitations

Requirement: Discuss limitations of the evidence included in the review

Compliance: ✓ COMPLIANT
Response: Limitations include methodological constraints, heterogeneity, publication bias risk, and scope boundaries. Limitation section addressing methodological limitations of the review (not just literature limitations), organized by type with impact assessment, mitigation strategies documented, implications for interpretation specified, balanced with acknowledgment of strengths.
Evidence:
LIMITATIONS AT REVIEW LEVEL:
1. Search and Selection Limitations:
Language Restrictions:

- **Limitation:** English and Indonesian only; excluded Chinese, Spanish, Arabic, German, Portuguese
- **Impact:** Potentially missed studies from major IoT research countries (China), Latin America, Middle East, continental Europe
- **Direction of Bias:** Unknown; may miss cultural variations, region-specific barriers, alternative theoretical frameworks
- **Mitigation Attempted:** Eight databases including regional (Scopus, regional journals), Google Scholar for gray literature

Database Coverage:

- **Limitation:** Despite 8 databases, some indexing gaps; conference proceedings partially excluded
- **Impact:** May miss emerging findings, industry implementations, practical case studies
- **Direction of Bias:** Likely toward academic, peer-reviewed positive findings
- **Mitigation Attempted:** Supplementary searches

2. Screening and Extraction Limitations:
Single Reviewer Screening:

- **Limitation:** Title/abstract screening conducted by first author, second reviewer providing oversight and validation throughout the process. Full-text screening conducted by both reviewers with independent eligibility judgments and documented disagreement resolution (κ = 0.89, almost perfect agreement).
- **Impact:** Absence of fully parallel independent screening at title/abstract stage may introduce selection bias at that stage.
- **Mitigation:** Standardized evaluation form, clear criteria, systematic documentation in Zotero screening log; full-text stage addressed through bilateral dual-reviewer assessment with kappa verification.
- **Recommendation:** Future reviews should employ independent dual screening at all stages.

Data Extraction Validation:

- **Limitation:** Single extractor with verification of quantitative data only
- **Impact:** Potential extraction errors, inconsistencies in interpretation

- **Mitigation:** Standardized piloted form, team review of extraction tables, verification of numeric data
- **Recommendation:** Dual extraction with disagreement resolution strengthens rigor

3. Quality Assessment Limitations:

Appraisal Subjectivity:

- **Limitation:** MMAT criteria involve evaluative judgment
- **Impact:** Quality ratings may vary between reviewers
- **Mitigation:** Systematic application of MMAT
- **Transparency:** All ratings documented in Appendix 5

4. Synthesis and Analysis Limitations:

Vote Counting Approach:

- **Limitation:** Studies counted equally regardless of sample size, effect size, or quality in frequency percentages
- **Impact:** Small, low-quality studies have same weight as large, rigorous studies; percentages can mislead
- **Mitigation:** Reported absolute numbers, effect sizes where available, quality-weighted sensitivity analyses, certainty ratings
- **Alternative:** Meta-analysis not feasible due to heterogeneity; narrative synthesis necessary

Limited Subgroup Analysis:

- **Limitation:** Many studies didn't report sufficient data for systematic subgroup analyses by age, literacy, technology type
- **Impact:** Moderator findings rely on studies explicitly examining interactions
- **Mitigation:** Reported subgroups where data available; acknowledged gaps
- **Recommendation:** Future research should routinely report subgroup findings

5. Generalizability Limitations:

Geographic Representation Bias:

- **Limitation:** 71% from Asia and Europe, 0% from Africa/Latin America/Oceania
- **Impact:** Findings may not apply to underrepresented regions with different:
    - Healthcare systems (universal vs. private)
    - Cultural norms (collectivism vs. individualism, privacy expectations)
    - Technological infrastructure (high vs. low bandwidth)
    - Economic contexts (ability to pay, insurance coverage)
- **Example:** Privacy concerns may be culturally shaped; cost barriers may be higher in low-resource settings
- **Mitigation:** Noted geographic patterns in findings; qualified generalizations
- **Critical Need:** Research in Africa, Latin America, rural areas, low-resource settings

Rapid Technology Evolution:

- **Limitation:** 2016-2018 studies describe technologies since superseded (capabilities, security, interfaces changed)
- **Impact:** Earlier findings may not apply to current IoT implementations
- **Mitigation:** Weighted recent studies; focused on human factors

6. Scope Limitations:
Patient Perspective Exclusivity (STRENGTH & LIMITATION):

- **Strength:** Deep focus on end-user acceptance
- **Limitation:** Excludes provider, administrator, policy perspectives crucial for implementation
- **Impact:** Incomplete picture of adoption ecosystem; multi-stakeholder barriers not fully addressed
- **Justification:** Deliberate scope decision to fill patient perspective gap
- **Recommendation:** Findings should be integrated with provider/system-level reviews for comprehensive understanding

OTHER INFORMATION
Item 25: Registration and Protocol
Requirement: Provide registration information including protocol registration number
Compliance: ✓ COMPLIANT (with transparency)
Response: Transparent statement that review was not prospectively registered, rationale provided, compensatory transparency measures documented (OSF repository with all materials), minor protocol deviations disclosed.
Evidence:
Registration Status: This systematic literature review was **not prospectively registered** in PROSPERO or other systematic review registries.
Rationale for Non-Registration:

- Review was initiated before protocol development was finalized
- PROSPERO registration primarily targets health intervention reviews; methodology reviews and acceptance studies less commonly registered
- Decision made to prioritize search and rigorous conduct over prospective registration

Protocol Development: Despite non-registration, we developed and followed a detailed protocol:

- Protocol finalized: December 2024 (before study selection began)
- Protocol components: Research questions, PICO framework, inclusion/exclusion criteria, search strategy, screening procedures, data extraction plan, quality assessment approach, synthesis methods

Transparency Measures to Compensate:

1. **Complete Methods Documentation:** Full protocol details provided in manuscript Methods section

2. **PRISMA 2020 Compliance:** 27-item checklist completed (this document)
3. **Open Materials:** All search strategies, screening decisions, extraction data, quality assessments deposited at Open Science Framework
4. **OSF Repository:** DOI 10.17605/OSF.IO/VY67B contains:
    - Detailed protocol document
    - Complete search strategies for all databases
    - Screening decisions log with exclusion reasons
    - Data extraction forms (blank and completed)
    - Quality assessment forms with ratings
    - PRISMA checklist
    - Analysis files

Future Recommendations:

- **For Other Researchers:** Prospective registration in PROSPERO, OSF, or other registry recommended for all systematic reviews
- **For This Team:** Future reviews will be prospectively registered before study selection begins

Item 26: Support/Funding

Requirement: Describe sources of financial or other support for the review; describe roles of funders

Compliance: ✓ COMPLIANT

Response: Funding statement provided, institutional support described with clear statement of non-involvement in research decisions, no conflicts of interest declared.

Evidence:

Funding Statement:

This systematic literature review **received no specific funding** from any funding agency in public, commercial, or not-for-profit sectors.

No Commercial Support:

- No funding from industry, technology companies, or commercial entities
- No in-kind support from IoT device manufacturers or healthcare technology companies
- No pharmaceutical or medical device company involvement

Conflicts of Interest: None declared. The authors have no financial or non-financial conflicts of interest to disclose related to this research.

Independence Statement: This review was conducted completely independently of any external funding sources or commercial interests. All decisions regarding design, conduct, analysis, and reporting were made solely by the research team based on scientific merit and rigor.

Item 27: Competing Interests

Requirement: Declare any competing interests

Compliance: ✓ COMPLIANT
Response to Reviewers: Comprehensive conflict of interest statement with specific financial and non-financial disclosures, confirmed no competing interests.
None declared. The authors have no financial or non-financial conflicts of interest to disclose related to this research.
